# Quantitative Proteome Profiling of *Coxiella burnetii* Reveals Major Metabolic and Stress Differences Under Axenic and Cell Culture Cultivation

**DOI:** 10.3389/fmicb.2019.02022

**Published:** 2019-09-18

**Authors:** Jiri Dresler, Jana Klimentova, Petr Pajer, Barbora Salovska, Alena Myslivcova Fucikova, Martin Chmel, Gernot Schmoock, Heinrich Neubauer, Katja Mertens-Scholz

**Affiliations:** ^1^Military Health Institute, Prague, Czechia; ^2^Faculty of Military Health Sciences, University of Defence, Hradec Kralove, Czechia; ^3^Department of Genome Integrity, Institute of Molecular Genetics of the Czech Academy of Sciences, Prague, Czechia; ^4^Department of Biology, Faculty of Science, University of Hradec Kralove, Hradec Kralove, Czechia; ^5^Department of Infectious Diseases, First Faculty of Medicine, Charles University and Military University Hospital Prague, Prague, Czechia; ^6^Institute of Bacterial Infections and Zoonoses, Friedrich-Loeffler-Institut, Jena, Germany

**Keywords:** *Coxiella burnetii*, axenic culture, semi-quantitative proteomics, oxidative stress response, type IV B secretion system, *dot/icm*, Sec translocon, proteome

## Abstract

*Coxiella burnetii* is the causative agent of the zoonotic disease Q fever. To date, the lipopolysaccharide (LPS) is the only defined and characterized virulence determinant of *C. burnetii*. In this study, proteome profiles of *C. burnetii* Nine Mile phase I (RSA 493, NMI) and its isogenic Nine Mile phase II (RSA 439 NMII) isolate with a deep rough LPS were compared on L-929 mouse fibroblasts and in complex (ACCM-2), and defined (ACCM-D) media. Whole proteome extracts were analyzed using a label-free quantification approach. Between 659 and 1,046 *C. burnetii* proteins of the 2,132 annotated coding sequences (CDS) were identified in any particular experiment. Proteome profiles clustered according to the cultivation conditions used, indicating different regulation patterns. NMI proteome profiles compared to NMII in ACCM-D indicate transition from an exponential to a stationary phase. The levels of regulatory proteins such as RpoS, CsrA2, UspA1, and UspA2 were increased. Comparison of the oxidative stress response of NMI and NMII indicated that ACCM-2 represents a high oxidative stress environment. Expression of peroxidases, superoxide dismutases, as well as thioredoxins was increased for NMI. In contrast, in ACCM-D, only osmoregulation seems to be necessary. Proteome profiles of NMII do not differ and indicate that both axenic media represent similar oxidative stress environments. Deep rough LPS causes changes of the outer membrane stability and fluidity. This might be one reason for the observed differences. Proteins associated with the T4SS and Sec translocon as well as several effector proteins were detectable under all three conditions. Interestingly, none of these putatively secreted proteins are upregulated in ACCM-2 compared to ACCM-D, and L-929 mouse fibroblasts. Curiously, a higher similarity of proteomic patterns (overlapping up- and downregulated proteins) of ACCM-D and bacteria grown in cell culture was observed. Particularly, the proteins involved in a better adaptation or homeostasis in response to the harsh environment of the parasitophorous vacuole were demonstrated for NMI. This semi-quantitative proteomic analysis of *C. burnetii* compared axenically grown bacteria to those propagated in cell culture.

## Introduction

*Coxiella burnetii*, a Gram-negative and obligate intracellular bacterium, is the etiological agent of query (Q) fever. This almost worldwide-distributed zoonotic disease manifests as an acute, flu-like illness or as a potentially life-threatening chronic disease in humans (Maurin and Raoult, 1999). *C. burnetii* has a broad host spectrum (Angelakis and Raoult, 2010) and the bacteria are transmitted via inhalation of contaminated aerosols.

*Coxiella burnetii* passively enters its primary target cells, alveolar macrophages, and traffics through the endocytic cascade (Graham et al., 2013). The intracellular niche, named Coxiella-containing vacuole (CCV), shows all characteristics of a terminal phagolysosome, but is actually a highly specific, and pathogen-directed niche. *C. burnetii* employs a type IV B secretion system (T4SS), also known as Dot/Icm (defect in organelle trafficking/intracellular multiplication) apparatus, to secrete effector proteins into the cytosol of the host (Zamboni et al., 2003; Chen et al., 2010). To date, more than 100 effectors have been described, but only few of them are known to subvert the host cell-signaling pathways, including vesicle trafficking, apoptosis, autophagy, and immune responses, to ensure intracellular survival of *C. burnetii* (Larson et al., 2013; Moffatt et al., 2015; Mansilla Pareja et al., 2017; Schäfer et al., 2017; Weber et al., 2018). Within the CCV, bacteria are exposed to a variety of anti-microbial agents such as oxidative stress, low pH, defensins, and proteases (Voth and Heinzen, 2007; van Schaik et al., 2013). *C. burnetii* is highly adapted to this unique niche and employs several antioxidative stress strategies, such as expression of DNA repair genes and detoxification of reactive oxygen species (ROS) and reactive nitrogen species (RNS) released by the host (Briggs et al., 2008; Mertens et al., 2008; Siemsen et al., 2009; Hill and Samuel, 2011; Brennan et al., 2015).

Upon frequent passaging in immune incompetent hosts or axenic cultivation, *C. burnetii* undergoes a phase variation (Hackstadt et al., 1985). This variation is characterized by a decreasing length of the O-antigen of the lipopolysaccharide (LPS) accompanied by a loss in virulence (Ftácek et al., 2000; Andoh et al., 2005; Beare et al., 2018). From the *C. burnetii* Nine Mile strain, two clonal isolates exist: virulent phase I (RSA 493, clone 7, NMI) with a full-length LPS, and avirulent phase II (RSA 439, clone 4, NMII) with a deep rough LPS. The major difference between these clones is a deletion of ~26 kb within a genome region associated with LPS biosynthesis (Hoover et al., 2002; Millar et al., 2017).

*C. burnetii* isolates display genetic heterogeneity and circulating predominant genotypes have been described (Hendrix et al., 1991; Chmielewski et al., 2009; Russell-Lodrigue et al., 2009; Roest et al., 2011; Boarbi et al., 2014; Frangoulidis et al., 2014). Observed differences in the pathogenic potential of isolates may be the result of either a variable repertoire of virulence factors or due to predisposed immunological properties of the host. Thus, genetic differences have to be validated by classical genetic approaches and on the proteomic level. With the development of an axenic cultivation method, genetic modification, growth behavior, and production of whole-cell antigen free from host material for genome sequencing or proteome analyses became feasible (Omsland et al., 2009; Sandoz et al., 2016a).

A genome-wide transcriptional study comparing *in vivo* model (mice) with *in vitro* cell-based and cell-free cultivation revealed major differences in expression, but the corresponding proteomic study is still missing (Kuley et al., 2015a). Previous proteomic studies based on gel electrophoresis compared NMI and NMII grown in embryonated hen eggs (Skultety et al., 2005, 2011; Samoilis et al., 2007) and identified tetracycline resistance mechanisms or virulence-associated proteins (Samoilis et al., 2010; Flores-Ramirez et al., 2014).

Major drawbacks of the gel-based approaches are that relatively high amount of protein sample is needed and also their labor-intensiveness. Moreover, labeling-based techniques are limited by the need of expensive consumables, an inability to add further samples into the experiment, and a limited number of comparable groups (Megger et al., 2013). On the contrary, label-free quantification (LFQ) does not require any labeling step, and relies only on spectral counting or MS intensity of peptide signal of the quantified feature.

The aim of the presented study is to determine differences in proteome profiles of *C. burnetii* under axenic cultivation and cell culture propagation by quantitative proteomics. We choose the isogenic strains NMI and NMII for comparison. These differ only in length of the LPS, replicate in indistinguishable CCVs, and show similar growth rates in primary monocyte-derived macrophages (Howe et al., 2010) and THP-1 cells. However, NMII fails to replicate in immunocompetent mice (Andoh et al., 2005) and NMII LPS does not confer protection in challenge experiments (Zhang et al., 2007). The LPS plays an important physiological role, besides barrier against antibiotics or host defense factors. It stabilizes the membrane due to the interaction of the O-polysaccharide portion and binding of divalent cations. Deep rough LPS leads to the accumulation of phospholipid patches, higher membrane fluidity, and higher permeability for lipophilic compounds. Further, truncation of the LPS affects correct folding and insertion of outer membrane proteins (OMPs) (Nikaido, 2003; Wang et al., 2015). Based on quantitative comparison of NMI and NMII *in vitro*, we want to establish a method to connect genomic data with expression profiles for identification of isolate-specific traits that will support the described isolate-specific virulence pattern. Therefore, we performed semi-quantitative analysis of NMI and NMII propagated in two different axenic media and in L-929 mouse fibroblasts employing an LFQ approach.

## Materials and Methods

### Bacterial Strains and Growth Conditions

*C. burnetii* Nine Mile phase I (RSA 493, NMI) and phase II (RSA 439, NMII) propagated in L-929 mouse fibroblasts were used for inoculation of axenic acidified citrate cysteine medium 2 (ACCM-2) and defined ACCM (ACCM-D) as described previously (Omsland et al., 2011; Sandoz et al., 2016a; Sanchez et al., 2018). Briefly, 1-l cultures were inoculated with 1E+05 GE/ml (genome equivalents) and incubated until they reached a density of 1E+07 to 1E+08 GE/ml at 37°C with 5% CO2 and 2.5% O2. This was archived after 7 or 10 days in ACCM-2 and ACCM-D. Cultures were harvested by centrifugation (21,200 × *g*; 15 min; 4°C) and resuspended in 270 mM sucrose solution containing 10% (v/v) glycerol for storage at −80°C. For propagation of *C. burnetii* in L-929 mouse fibroblasts (DMEM medium, 5% heat inactivated fetal bovine serum), cells were inoculated with a multiplicity of infection (MOI) of 100 and incubated at 37°C and 5% CO2. Infection was visually assessed and cultures were harvested when nearly 80% of cells were infected. Briefly, cell layers were scraped, centrifuged (1,000 × *g*, 5 min, 4°C), and resuspended in 1 ml of 270 mM sucrose solution containing 10% (v/v) glycerol for storage at −80°C. All laboratory procedures involving the handling of live bacteria of NMI were carried out in a Biosafety Level 3 laboratory.

### DNA Isolation and Quantification by Real-Time PCR (qPCR)

Bacterial quantification was carried out as previously described by real-time PCR (qPCR) using the isocitrate dehydrogenase encoding gene (*icd*) as target (Klee et al., 2006). Detailed description of the procedure is described in the Supplementary Methods section.

### Proteomic Sample Preparation

The bacterial cells from axenic media and L-929 cell-based cultures (Table S1) were pelleted by centrifugation (18,000 × *g*; 20 min; 4°C) and washed with 300 μl of phosphate-buffered saline (PBS, pH 7.4). The resulting pellets were resuspended in 0.1% RapiGestTM SF (Yu et al., 2003) (Waters, Manchester, UK) in 100 μl of 50 mM Tris, pH 7.5, and heated for 10 min at 95°C. After cooling down, 200 μl of 0.1% RapiGestTM SF in 8 M guanidinium chloride (Sigma-Aldrich) was added an incubated for 20 min. The protein sample(s) were stored at −80°C until samples were proven free of viable bacteria. Because of the potential of interfering substances in the supernatant, the following sample preparation workflow was applied and based on Filter aided sample preparation (FASP) (Wiśniewski et al., 2009). Briefly, inactivated samples were transferred onto Amicon® Ultra 10-kDa filters (Millipore) and washed twice with 100 mM ammonium bicarbonate (Sigma-Aldrich). Subsequently, proteins were quantified by bicinchoninic acid assay (QuantiPro™ BCA Assay Kit, Sigma-Aldrich) (Smith et al., 1985; Table S1). The samples were then reduced with 100 mM Tris (2-carboxyethyl) phosphine hydrochloride (TCEP, Sigma-Aldrich) and alkylated with 300 mM iodoacetamide (Sigma-Aldrich). Finally, the samples were digested with 2 μg of sequencing grade trypsin (Promega) overnight at 37°C. Empore™ SPE Cartridges, C18, standard density, bed I.D. 4 mm (Sigma-Aldrich) were used to desalt peptide mixtures before drying to completion in a speed-vac. Before mass spectrometry analysis, the samples were resuspended in 30 μL of 2% acetonitrile (ACN)/0.1% trifluoroacteic acid. The samples were further analyzed by LC-MS/MS techniques involving targeted mass spectrometry and LFQ. Detailed description of procedure of proteomic sample preparation and data processing is described in the Supplementary Methods section.

### Evaluation of Up- and Downregulated Proteins Under Axenic Cultivation and Under Cell Culture Propagation

The selection was based on a combination of functional annotation enrichment analysis and further statistical techniques as described below. The imputation of missing values from a normal distribution (Gaussian distribution width 0.3 SD and down-shift 1.8 SD of the original data) was performed, and proteins were annotated by Gene Ontology (GO) terms and UniProt (www.uniprot.org) keywords for *C. burnetii* (strain RSA 493/Nine Mile phase I, downloaded on April 2, 2016, 1,816 sequences). For comparison of axenic media replicates, ANOVA (permutation-based FDR 5%, S0 = 0) was used to identify significant differences in protein expression between individual groups. For comparison of cell-based cultures, Student's *t*-test was applied to identify proteins differentially expressed between NMI- and NMII-infected L-929 sample groups at a 5% threshold, and a permutation-based FDR was applied at a 5% threshold. Only ANOVA significant/Student's *t*-test significant hits were included for subsequent hierarchical clustering using Euclidean distances to group proteins with similar expression profiles.

## Results

Using the LFQ approach described in this study, between 659 and 1,046 *C. burnetii* proteins of 2,132 annotated CDS (coding sequences) were identified in each sample (Table S1). For NMI and NMII propagated in both axenic media (ACCM-2 and ACCM-D), a total of 1,075 quantifiable proteins specific for *C. burnetii* were detected and analyzed (Table S2). In L-929 cell-based cultures, a total of 906 *C. burnetii*-specific proteins were quantifiable and were identified (Table S3). The observed quantities of the proteins are depicted as log2 transformation of LFQ intensities and include the values imputed by Perseus-type value imputation (Cox et al., 2011, 2014). The values ranging from 21 to 32 reflect the dynamic range of the mass spectrometry-based workflow. Among these proteins, 772 were significantly up- or downregulated (ANOVA test, *q* ≤ 0.05) in axenic media experiments and 119 were significantly different (Student's *t*-test, *q* ≤ 0.05) in cell-based experiments (Tables S2, S3).

**Figure 1:**
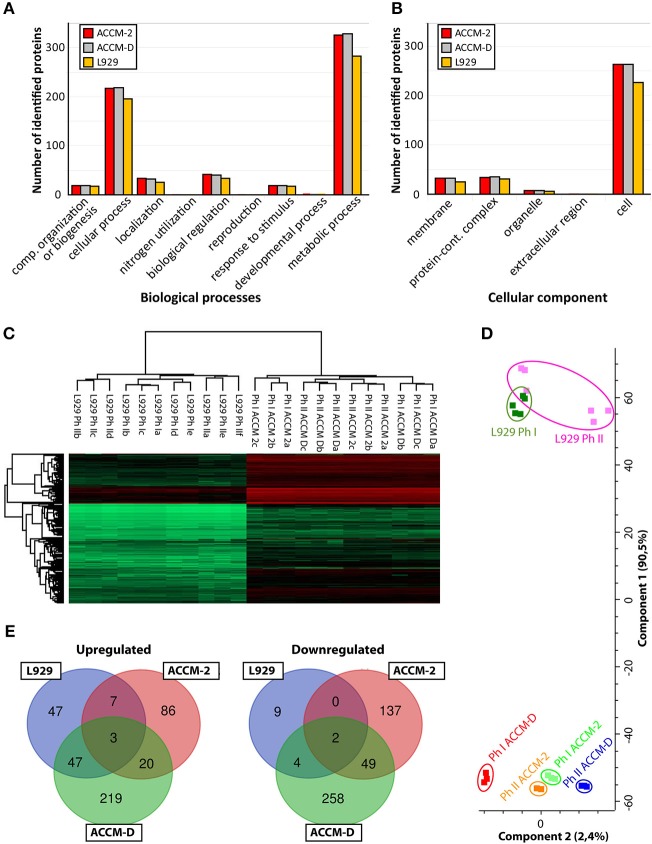
Overview of *C. burnetii* proteome profiles under three different culture conditions. **(A)** Mapping of biological pathways utilized by *C. burnetii* during ACCM-2, ACCM-D, or L-929 propagation and **(B)** mapping. **(B)** Mapping of cellular component assignment utilized by *C. burnetii* during ACCM-2, ACCM-D, or L-929 propagation using the PANTHER classification tool. **(C)** Hierarchical clustering of protein expression values based on label-free proteome quantification. LFQ values vs. biological replicates (designated as a, b, and c, or up to f for L929-based experiments) from particular samples. The color code indicates the LFQ values abundance of the quantifiable proteins (red, most abundant; green, least abundant). **(D)** Principal component analysis (PCA) of the LFQ intensities obtained from biological replicates of each representative group. **(E)** The number of statistically significant upregulated (left) and downregulated (right) proteins of *C. burnetii* Nine Mile phase I compared to *C. burnetii* Nine Mile phase II. Overlapping regions or identifications indicate up- or downregulated proteins under more than one culture condition.

Pathway mapping of proteins identified under different culture conditions (ACCM-2, ACCM -D, cell culture) was done with PANTHER classification tool (Thomas, 2003; Mi et al., 2013) (ver. 14.1). For each culturing condition, roughly 50% of the identified proteins could be assigned to a biological pathway, as shown in Figure 1A. For ACCM-2 cultivation, 660 of 1,055 quantifiable proteins were assigned to various biological processes. Similar results were obtained for ACCM-D with 661 of 1,047 quantifiable proteins and for cell culture samples with 578 of 906 quantifiable proteins with functional assignment. Cellular component analysis showed that 337 proteins from bacteria cultivated in ACCM-2, 339 from ACCM-D, and 290 from cell culture were assigned. Most other proteins are without any annotated function or without cellular component assignment (Figure 1A).

### Comparison of Proteomes of Individual Experiments

To assess the applicability of the LFQ approach, we examined the similarity of individual proteomes using hierarchical clustering and principal component analysis (PCA). Unsupervised cluster analysis of protein expression profiles was performed using Euclidean distances. Statistical procedures were performed using the computational platform Perseus (Cox et al., 2014). Both hierarchical clustering (Figure 1B) and PCA (Figure 1C) generated six distinctive groups encompassing each biological triplicate of analyzed *C. burnetii* phase variant under different conditions. Differences in proteome profiles are predominant between axenic grown and tissue culture propagated bacteria. Proteomes of NMI and NMII grown in ACCM-2 and ACCM-D clustered more closely together than proteomes of L-929 propagated bacteria (Figure 1D). Only proteins whose expression is significantly different (ANOVA, *q* ≤ 0.05) in axenic media conditions and significantly different in cell-based experiment (Student's *t*-test, *q* ≤ 0.05) were further evaluated. As demonstrated in Figure 1D, cultivation in ACCM-D and L-929 revealed 50 common upregulated proteins, whereas only 10 were common between ACCM-2, and L-929. A similar trend was observed for the downregulated group of proteins: under ACCM-D and L-929 culture conditions, the same six proteins were downregulated, whereas only two were common for ACCM-2 and L-929 (Figure 1E). These data imply the applicability of the established workflow for the analysis of total protein expression.

### *C. burnetii* Proteome Profiles Differ Accordingly to Their Growth Condition and Growth State

Proteome analyses were performed with *C. burnetii* NMI and NMII cultures, propagated in ACCM-2 and ACCM-D until a density of 1E+07 to 1+E08 GE/ml was observed, respectively (Table S1). Infected L-929 mouse fibroblasts were cultured until nearly 80% of cells were visually positive for *C. burnetii*-containing vacuoles (CCV). Under these three groups, NMI, and NMII showed different proteome profiles accordingly to the growth media used and thus imply different growth states or stress conditions.

### Proteome Profiles of NMI Compared to NMII in ACCM-D Resemble a Stationary Phase Growth Pattern

For NMI compared to NMII propagated in ACCM-D or ACCM-2, a total of 607, and 305 up- or downregulated proteins were detected, respectively. In ACCM-D, 292 proteins were upregulated and 315 were downregulated in NMI compared to NMII. In ACCM-2, only 115 upregulated and 190 downregulated proteins were detected in NMI compared to NMII.

The comparison of NMI with NMII in ACCM-D indicates a stationary phase growth pattern for NMI. Many proteins of the translation machinery, such as 50S and 30S ribosomal subunit proteins, translation initiation factors (IF-2, IF-3), translation elongation factors (EF-G, EF-Ts), and ribosomal maturation factor RbfA as well as the ribosome-associated inhibitor A (RaiA, CBU_0020) are downregulated. CsrA2 (CBU_1050), a stress response regulator, is upregulated. In addition, caseinolytic proteases (Clp, chaperon), such as ClpA and ClpP-like proteins CBU_0353, CBU_1483, and CBU_1538, were upregulated for NMI in ACCM-D.

Exponential phase transcriptional factors Fis and RpoD are downregulated. RpoS, the primary sigma factor during stationary phase, and SpoT, which positively regulates *rpoS* expression, are upregulated in NMI compared to NMII grown in ACCM-D.

Several proteins associated with cell wall remodeling are upregulated in NMI compared to NMII in ACCM-D. CopB (CBU_0092), which coordinates peptidoglycan synthesis and outer membrane constriction during cell division, was upregulated. Further, several peptidoglycan and cell wall biogenesis proteins, including the previously described CBU_0419, CBU_0925, EnhB.1, EnhC, EnhA.4, and EnhA.5, were upregulated in NMI compared to NMII in ACCM-D (Sandoz et al., 2016b).

Three out of 16 LCV-associated proteins, CBU_0658 (unknown function), Bcp, and UspA1, stress associated proteins, were over 2-fold upregulated in NMI compared to NMII in ACCM-D (McCaul et al., 1991; Seshadri et al., 1999; Varghees et al., 2002; Coleman et al., 2007; Hicks et al., 2010; Sandoz et al., 2016b). Similar results were obtained for SCV-associated proteins. Two proteins of unknown function, CBU_0961 and CBU_1561, as well as the SCV-specific protein ScvA were >2-fold upregulated in ACCM-D for NMI compared to NMII (McCaul et al., 1991; Heinzen et al., 1992; Varghees et al., 2002; Coleman et al., 2007).

An overview of selected proteins indicating a stationary growth pattern for NMI compared to NMII in ACCM-D, including their fold changes, are listed in Table 1. Taken together, these data lead to the hypothesis that some proteins of NMI compared to NMII propagated in ACCM-D seem to mimic transition from an exponential to a stationary phase.

**Table 1:** Overview of selected *C. burnetii* proteins involved in entering of NMI into the stationary phase in ACCM-D.

	**Locus**	**Protein**	**Function**	**NMI to NMII**			**NMI**	**NMII**
				**ACCM-D**	**ACCM-2**	**L-929**	**ACCM-D to ACCM-2**	**ACCM-D to ACCM-2**
Regulation	CBU_0303	SpoT	GTP pyrophosphokinase	3.59	1.46	NaN	1.59	0.65
	CBU_1050	CsrA2	Translational regulator	0.10	0.13	1.53	2.89	3.72
	CBU_1596	RpoD	Sigma factor	0.60	0.63	0.97	1.01	1.06
	CBU_1669	RpoS	Sigma factor	0.62	0.36	0.75	659.15	382.27
	CBU_1916	UspA2	Universal stress protein	3.25	0.17	0.61	5.82	0.30
	CBU_1983	UspA1	Universal stress protein	2.14	0.49	3.13	2.24	0.51
Chaperon	CBU_0353		ClpP-like protein	21.28	0.43	2.54	24.49	0.49
	CBU_1196	ClpA	ATP-dependent clp protease ATP-binding subunit	1972.42	0.00	0.83	912.01	0.00
	CBU_1483		ClpP-like protein	5.73	0.09	13.40	6.62	0.10
	CBU_1538		ClpP-like protein	1.44	0.48	0.76	1.29	0.43
Cell envelope	CBU_0419		Polysaccharide deacetylase family	629.51	0.07	0.19	81.28	0.01
	CBU_0915	EnhB.1	Enhanced entry protein	19.05	0.17	6.76	26.48	0.23
	CBU_0925		Membrane-bound lytic murein transglycosylase B	2133.04	0.06	8.83	521.19	0.01
	CBU_1136	EnhC	Enhanced entry protein	492039.54	0.00	10.69	2157744.41	0.00
	CBU_1138	EnhA.4	Enhanced entry protein	13997.48	0.02	8.57	4324.11	0.01
	CBU_1394	EnhA.5	Enhanced entry protein	1277.54	0.15	35.67	1045.55	0.13
SCV	CBU_0961		Unknown	0.41	0.25	NaN	2.99	1.84
	CBU_1267.1	ScvA	SCV-specific protein ScvA	346928.52	0.00	13.00	328256.50	0.00
	CBU_1561		Unknown	225.94	0.37	NaN	706.32	1.15

*Fold-increase in protein LFQ intensities for each analysis in axenic media with a corresponding color code is depicted. The ACCM-2 column depicts ratio of LFQ values for NMI to NMII cultivated in ACCM-2. The same formula was used for next columns in ACCM-D and L929. Values in the column ACCM-D to ACCM-2 depict the ratio between NMI in ACCM-D to ACCM-2, similarly as in the next column for NMII. A value of 1 indicates no changes. A value of 2 indicates two fold change upregulation of NMI compared to NMII—tones of red color, a value of 0.01 indicates 100-fold change downregulation of NMI compared to NMII—tones of blue color. NaN—values not detected. A value of 0 means more than 5,000-fold change downregulation of NMI compared to NMII (the values are rounded)*.

Proteome Profiles of NMI Compared to NMII in ACCM-2 Reveals No Major Differences.

Overall, proteome analysis of ACCM-2-grown bacteria identified a smaller number of regulated proteins when comparing NMI to NMII. The 115 upregulated or 190 downregulated proteins in NMI compared to NMII indicated no specific growth phase. Regulations of proteins associated with the translational machinery, post-translational modification (chaperons), as well as co-factor synthesis were partly up- and downregulated. Interestingly, the ATP synthase (AtpA, AtpD, AtpG, AtpF) was upregulated in NMI compared to NMII in ACCM-2. These data imply the absence of major metabolic differences of NMI and NMII grown in ACCM-2.

### NMI and NMII Respond Differently to Stress in Axenic Media and Cell Culture

Comparison of the proteome profiles of NMI with NMII, propagated in either ACCM-2 or ACCM-D, shows major differences in response to oxidative stress. In ACCM-2, the alkyl hydroperoxide reductases AhpC1 and AhpC2, cytoplasmic superoxide dismutase B (SodB), and the periplasmic SodC are over 2-fold upregulated for NMI compared to NMII. In addition, glutathione (GshAB, glutathione synthesis) and a glutaredoxin-like protein (CBU_0583) are upregulated in NMI compared to NMII when propagated in ACCM-2. On the contrary, both AhpCs are downregulated in NMI compared to NMII when propagated in ACCM-D.

Upregulation of a glycine betaine transport system, CBU_0177/CBU_0178, in NMI compared to NMII propagated in ACCM-D implies an active osmoregulation. Interestingly, the same upregulation was detected for NhaP.1, a Na2+/H+ antiporter that extrudes sodium in exchange for external protons. During propagation in L-929 mouse fibroblasts, most oxidative stress response proteins are upregulated as well as the glycine betaine transport system when comparing NMI to NMII. An overview of selected *C. burnetii* proteins involved in stress response of NMI and NMII in axenic media and cell culture, including their fold changes, is provided in Table 2.

**Table 2:** Overview of selected *C. burnetii* proteins involved in different responses of NMI and NMII to stress in axenic media and cell culture.

	**Locus**	**Protein**	**Function**	**NMI to NMII**			**NMI**	**NMII**
				**ACCM-D**	**ACCM-2**	**L-929**	**ACCM-D to ACCM-2**	**ACCM-D to ACCM-2**
Oxidative stress	CBU_0583		Glutaredoxin	0.30	8.26	0.74	0.04	1.03
	CBU_0963	Bcp	Putative peroxiredoxin bcp	1.81	0.57	3.88	2.30	0.72
	CBU_1193	TrxB	Thioredoxin reductase	0.61	1.72	1.97	0.53	1.50
	CBU_1476	OxyR	Hydrogen peroxide-inducible genes activator	0.53	1.66	2.99	0.35	1.10
	CBU_1477	AhpC2	Hydroperoxide reductase	0.37	17.89	NaN	0.04	1.73
	CBU_1706	AhpC1	Hydroperoxide reductase	0.53	2.48	1.55	0.25	1.20
	CBU_1708	SodB	Superoxide dismutase B	4.64	2.19	0.71	2.09	0.98
	CBU_1822	SodC	Superoxide dismutase C	3.95	11.32	2.56	0.00	0.00
	CBU_1874	GshA	Glutamate–cysteine ligase	1.74	5.46	3.07	0.27	0.83
	CBU_1875	GshB	Glutathione synthetase	6.46	5.18	855.89	0.60	0.48
	CBU_2087	Trx	Thioredoxin	1.57	0.54	1.77	2.70	0.93
Osmoregulation	CBU_1259	NhaP.1	Na+/H+ antiporter	77.91	0.12	NaN	60.25	0.09
	CBU_0177		Glycine betaine transport system permease protein	5.60	1.95	5.77	1.37	0.48
	CBU_0178		Glycine betaine transport ATP-binding protein	4.35	2.54	6.63	0.85	0.50

*Fold-increase in protein LFQ intensities for each analysis in axenic media with a corresponding color code is depicted. The ACCM-2 column depicts ratio of LFQ values for NMI to NMII cultivated under ACCM-2. The same formula was used for next columns in ACCM-D and L929. Values in the column ACCM-D to ACCM-2 depict the ratio between NMI in ACCM-D to ACCM-2, similarly as in the next column for NMII. A value of 1 indicates no changes. A value of 2 indicates twofold change upregulation of NMI compared to NMII—tones of red color, a value of 0.01 indicates 100-fold change downregulation of NMI compared to NMII—tones of blue color. NaN—values not detected. A value of 0 means more than 5,000-fold change downregulation of NMI compared to NMII (the values are rounded)*.

### Proteins Potentially Involved in Sec Translocation Are Upregulated in NMI

Components of the Sec–TolC pathway (SecABDFY, YajC, YidC, Fth, FtsY, LepB-1, and LepB-2, TolC) and previously described 24 Sec-secreted proteins were detected in samples harvested from axenic medium and cell culture. Additionally, two putatively secreted proteins, CBU_0110 and CBU_1764a, were only detectable in samples from cell culture but not from axenic media. Interestingly, potentially Sec-secreted proteins were upregulated in NMI compared to NMII in ACCM-D, but not in ACCM-2.

Other transporters, such as several ABC transporters, RND efflux pumps, AcrB-like efflux systems, and MFS superfamily transporters were upregulated during propagation in L-929 and ACCM-D for NMI compared to NMII. An overview of selected *C. burnetii* proteins involved in secretion systems in axenic media and cell culture is provided in Table 3.

**Table 3:** Overview of selected *C. burnetii* proteins involved in secretion systems in axenic media and cell culture.

	**Locus**	**Protein**	**Function**	**NMI to NMII**			**NMI**	**NMII**
				**ACCM-D**	**ACCM-2**	**L-929**	**ACCM-D to ACCM-2**	**ACCM-D to ACCM-2**
Sec translocon	CBU_0147	SecA	Protein translocase subunit	1.22	1.15	1.42	0.86	0.81
	CBU_0258	SecY	Protein translocase subunit	0.45	0.62	0.51	1.60	2.22
	CBU_0450	Ffh	Signal recognition particle (SRP) subunit FFH/SRP54	0.20	2.28	0.89	0.19	2.19
	CBU_1099	LepB-2	Signal peptidase I	1.95	1.24	13.09	1.22	0.78
	CBU_1141	SecF	Protein translocase subunit	2.14	1.57	1.48	2.21	1.62
	CBU_1142	SecD	Protein translocase subunit	2.12	1.04	1.38	3.11	1.53
	CBU_1143	YajC	Protein translocase subunit	4.73	4.80	2.12	0.80	0.81
	CBU_1504	LepB-1	Signal peptidase I	0.83	0.84	2.07	2.09	2.10
	CBU_1519	SecB	Protein export chaperone	0.74	0.93	2.53	0.62	0.79
	CBU_1903	FtsY	Cell division protein, SRP receptor	1.60	2.06	0.98	0.63	0.82
	CBU_1920	YidC	60 kDa inner membrane protein	0.48	0.75	1.58	1.14	1.79
TolC	CBU_0056	TolC	Type I secretion outer membrane protein	2.59	0.81	3.36	2.08	0.65
Secreted proteins	CBU_0378		Hypothetical membrane associated protein	24.73	2.77	0.55	0.86	0.10
	CBU_0482		Arginine-binding protein	25.40	0.35	1.08	9.77	0.14
	CBU_0562a		Unknown protein	14474.49	0.02	0.89	421.07	0.00
	CBU_0630	Mip	Macrophage infectivity potentiator	11.65	0.63	1.67	3.86	0.21
	CBU_0915	EnhB.1	Enhanced entry protein	19.05	0.17	6.76	26.48	0.23
	CBU_1095		Hypothetical exported protein	151.52	1.32	0.60	18.27	0.16
	CBU_1137	EnhB.2	Enhanced entry protein	41080.90	0.04	0.69	3973.96	0.00
	CBU_1138	EnhA.4	Enhanced entry protein	13997.48	0.02	8.57	4324.11	0.01
	CBU_1173		Unknown protein	36.47	1.99	3.51	1.66	0.09
	CBU_1404		Hypothetical exported protein	15851.24	2.13	7.03	35.90	0.00

*Fold-increase in protein LFQ intensities for each analysis in axenic media with a corresponding color code is depicted. The ACCM-2 column depicts ratio of LFQ values for NMI to NMII cultivated under ACCM-2. The same formula was used for next columns in ACCM-D and L929. Values in the column ACCM-D to ACCM-2 depict the ratio between NMI in ACCM-D to ACCM-2, similarly as in the next column for NMII. A value of 1 indicates no changes. A value of 2 indicates twofold change upregulation of NMI compared to NMII—tones of red color, a value of 0.01 indicates 100-fold change downregulation of NMI compared to NMII—tones of blue color. NaN—values not detected. A value of 0 means more than 5000-fold change downregulation of NMI compared to NMII (the values are rounded)*.

### Type IV B Secretion System and Effectors Are Expressed in Axenic Media and Cell Culture

Most components of the T4BSS were expressed under axenic and cell culture conditions. Only two structural components, IcmD and IcmH, were not detected in axenically grown bacteria. Additionally, four components, IcmD, IcmH, IcmT, and IcmC/DotE, were not detected in L-929 propagated bacteria. IcmB (DotP), IcmE (DotG), IcmG (DotF), IcmH, IcmK (DotH), IcmL.1, IcmL.2, IcmN (DotK), IcmO (DotL), and IcmX were found upregulated in NMI during L-929 infection when compared to NMII. IcmX, a secreted/released T4BSS protein (Luedtke et al., 2017), was upregulated exclusively in NMI under all cultivation conditions.

In our study, PmrA was expressed nearly two orders of magnitude higher in NMI compared to NMII when propagated in L-929. A similar upregulation of PmrA was found for NMI in ACCM-2, but not in ACCM-D. PmrB was also detected 1.5 times higher in NMI compared to NMII during propagation in L-929.

The detected amount of DotA (Luedtke et al., 2017) was not significantly changed during infection of L-929 and cultivation in ACCM-D for both isolates. On the contrary, the level of DotA was decreased in NMI compared to NMII in ACCM-2. Regarding some other T4BSS proteins, DotB, DotC, and DotD, no significant differences between NMI and NMII in axenic media were detectable.

Among other Dot/Icm substrates with predicted effector functions (van Schaik et al., 2013), only three proteins were found to be differentially regulated in this study. Two plasmid encoded proteins, CBUA0013 (Voth et al., 2011), and CBUA0023 (Voth et al., 2011), were observed upregulated in NMI compared to NMII during propagation in L-929 as well as in ACCM-D. In contrast, CBU_2078 (Chen et al., 2010) was observed in higher amounts in NMI compared to NMII during growth in L-929 cells and in ACCM-D. Out of over a 100 described T4BSS effector proteins (McDonough et al., 2012; Qiu and Luo, 2017), only 9 (CBU_0021, CBU_0175, CBU_0447, CBU_0794, CBU_0937, CBU_1379a, CBU_1425, CBU_1751, and CBU_2078) were detected, but with no fold change of semi-quantitative expression. Of these, seven were identified in a large-scale screen for translocation in *Legionella pneumophila*, and two, CvpB and Cig57, have a functional assignment (Chen et al., 2010; Latomanski et al., 2016; Martinez et al., 2016).

### LPS-Associated Proteins

The LPS is an amphiphilic OM structure with a hydrophobic lipid A membrane anchor, a core oligosaccharide, and highly variable O-specific polysaccharide chain (OPS). The exact structure and biosynthetic pathway of the LPS was not fully elucidated yet. Under all three conditions tested, several proteins associated with biosynthesis of the lipid A (LpxABCDH) and core oligosaccharide residues 3-deoxy-D-manno-oct-2-ulosonic acid (Kdo, KdsAB) and D-glycero-D-manno-heptose (Hep, GmhA, HldE, CBU_1996, CBU_0678) were detected. However, we did not detect any proteins from the OPS-associated genomic region CBU_0679 to CBU_0698 (deleted region in NMII), as well as in axenic media or in the cell-based cultures in NMI proteomes. This negative observation is especially interesting as these genes and their protein products are supposed to be a key genetic difference of NMI and NMII.

## Discussion

We used a combination of MS-based shotgun proteomics and LFQ approach for semi-quantitative analysis of proteomes of two closely related *C. burnetii* isolates, NMI, and NMII, under three different growth conditions. Namely, cell-free propagation in a complex (ACCM-2) and/or defined (ACCM-D) acidified citrate cysteine medium was compared to growth in L-929 mouse fibroblasts. The dominant difference between these two isolates is the severe truncation of the LPS in NMII associated with a large chromosomal deletion (Hoover et al., 2002). The published genome sequences of both isolates indicate small changes in the genome (partial deletions of homologs of CBU_0678, CBU_0698, and CBU_0918 and several SNPs) (Seshadri et al., 2003; Millar et al., 2017), which may lead to gross changes in the proteome. Together, this may cause pleiotropic phenotypes. The severe truncation of the LPS in NMII and increased amount of phospholipids (Frimmelová et al., 2016) in the outer membrane could lead to increased membrane fluidity. This increases the permeability for lipophilic compounds and affects correct insertion of OMPs into the membrane (Nikaido, 2003).

Both isolates were grown to a cell density of ~1E+07 to 1E+08 GE/ml, which was reached on day 7 in ACCM-2 and day 10 in ACCM-D. In addition, persistently infected L-929 mouse fibroblasts were used for comparison. The unlimited number of compared groups with the LFQ technique enables a semi-quantitative investigation of the proteomes. The applicability of this workflow was shown by hierarchical clustering (Figure 1C) and PCA (Figure 1D). Both methods generated six distinctive groups encompassing each biological triplicate of axenic or cell culture propagated bacteria. Previously published comparative studies either were based on gel electrophoresis (Skultety et al., 2011) or did not involve virulent phase I bacteria (Papadioti et al., 2012). The only proteomic study comparing NMI and NMII in embryonated hen eggs was qualitative (Skultety et al., 2011). Our semi-quantitative comparative analysis fills the gap between the previously published genome-wide transcriptional study and proteomic data (Kuley et al., 2015b).

Changes in the proteome profiles of NMI and NMII, under the conditions applied here, became only observable after application of stringent statistics (ANOVA significance for axenic and Student's *t*-test significance for infected L-929 samples). Overall, the highest amount of semi-quantifiable proteins was obtained from ACCM-D grown bacteria, followed by ACCM-2 and cell culture propagated bacteria. This clearly reveals the feasibility of the applied approach for a comparative analysis of closely related isolates. The involvement of two different time points (7 and 10 days for ACCM-2 and ACCM-D, respectively) and a persistently infected cell culture can introduce artificial conditions and potentially results in altered proteome profiles between the 7th and 10th day of infection, but comparable cell densities were reached at the selected time points. In order to follow only the most prominent proteomic changes, this part of study was performed in pentaplicates/hexaplicates for NMI and NMII, respectively. Furthermore, a stringent statistical workflow was applied. In summary, the L929 infection model showed substantial numbers of semi-quantifiable proteins and confirmed the higher relevancy of the ACCM-D medium. However, harvesting the cells at different time points, improved protocols for isolation of the bacteria and optimizing the sample preparation protocol could lead to a better comparability and a higher number of identifiable and semi-quantifiable proteins.

Comparing global differences between axenic media and cell-based cultures, the type of the media (ACCM-2 or ACCM-D) had a great impact on the difference of the proteome as demonstrated in a previously published transcriptomic study (Kuley et al., 2015b). A bigger overlap of regulated proteins was observed for ACCM-D and cell culture grown bacteria in contrast to ACCM-2, as shown in Figure 1E. This supports the relevance of ACCM-D not only as a growth medium but also as a CCV-like environment without host stress. Process mapping showed that most proteins were assigned to cellular and metabolic pathways for all three conditions tested (Figure 1A).

From the biological processes identified, proteins involved in stress response, Sec-dependent and T4BSS-dependent secretion, and LPS biosynthesis are of prominent interest. In ACCM-2 and ACCM-D, bacteria reach the late logarithmic or early stationary phase after 7 or 10 days, respectively (Omsland et al., 2011; Sandoz et al., 2016a). This was indicated for NMI in comparison to NMII in ACCM-D by downregulation of the translational machinery, including ribosomal proteins and translational initiation/elongation factors. Indeed, the ribosome-associated inhibitor A (RaiA, CBU_0020), which maintains translation accuracy during stress or upon entry into the stationary phase, was upregulated (Ueta et al., 2005). Of the translational regulators, CsrA1 (CBU0024) and CsrA2 (CBU_1050), which play an important role during shifting from exponential growth to stress survival, only CsrA2 is upregulated in NMI propagated in ACCM-D. The same upregulation of CsrA2 was observed by Sandoz et al. (2016b), for NMII propagated for up to 21 days in Vero cells (Romeo et al., 2013; Sandoz et al., 2016b). In addition, caseinolytic proteases (Clp, chaperon), such as ClpA and ClpP-like proteins CBU_0353, CBU_1483, and CBU_1538, were upregulated. They play an important role during stress response by assisting in proteolysis and disaggregation of proteins. For example, *Salmonella typhimurium clpA* and *clpB* mutants are highly sensitive against high temperature and oxidative stress (Sangpuii et al., 2018). Downregulation of Fis and RpoD, two exponential phase transcription factors, and upregulation of RpoS and its regulator SpoT point to stationary growth of NMI in ACCM-D. These data correlate with gene expression data during morphological differentiation from LCV to SCV (Sandoz et al., 2016b). In addition, the ScvA, a typical SCV-associated protein, was strongly upregulated in NMI after 10 days in ACCM-D (Coleman et al., 2004, 2007). These data imply the entry into the stationary phase and may indicate morphological differentiation (Moormeier et al., 2019). In contrast, the amount of semi-quantifiable proteins in ACCM-2 and L-929 propagated bacteria was much lower and not sufficient to identify a specific growth phase.

Interestingly, NMI, and NMII respond differently with regard to oxidative stress in ACCM-2 and ACCM-D. Previously, it was shown on the transcriptional level that first-generation ACCM-1 is associated with significant upregulation of genes involved in defenses against oxidative stress in NMII (Sandoz et al., 2014). Subsequent substitution of fetal bovine serum for methyl-β-cyclodextrin in ACCM-2 reduced this response, promoted developmental transition, and increased viability (Sandoz et al., 2014). Similar to ACCM-1, we observed upregulation of both superoxide dismutases (SodB, SodC), both reductases (AhpC1, AhpC2), as well as OxyR as stress response regulator in NMI compared to NMII in ACCM-2. These proteins are necessary for the direct detoxification of oxygen radicals (Mertens and Samuel, 2012; Brennan et al., 2015). In addition, repair of oxidized proteins by glutathione (radical sink) and the peroxiredoxin Bcp are upregulated in NMI.

In ACCM-D, direct detoxification and the repair of oxidized proteins are not upregulated in NMI. Instead, upregulation of a glycine betaine transport system, CBU_0177/CBU_0178 and a Na2+/H+ antiporter (NhaP.1) was observed. Enhanced import of glycine betaine protects against hyperosmotic stress (Pham et al., 2018). NhaP.1 is a Na2+/H+ antiporter that extrudes sodium in exchange for external protons. This implies that osmoregulation is important for NMI in ACCM-D, whereas direct detoxification is more relevant in ACCM-2. *C. burnetii* has a highly basic theoretical proteome with an average pI value for all predicted CDSs of 8.25. It was suggested that this basic proteome counterbalances the acidic environment of the CCV (Seshadri et al., 2003; Ihnatko et al., 2012). This implies that ACCM-D is not a harsh environment, which only requires osmoregulation.

For NMII, these data lead to the conclusion that both media represent a similar high oxidative stress environment. There are no major differences visible, when comparing NMII grown in ACCM-2 with ACCM-D (Table 3). The main difference between both *C. burnetii* isolates is the severe truncation of the LPS in NMII (Toman and Skultéty, 1996). The LPS is essential for Gram-negative bacteria. It functions as a permeability barrier for small hydrophobic compounds, salts, and detergents. Thus, it allows bacteria to survive in harsh environments and might explain the observed different response of NMI and NMII (Zhang et al., 2013).

The control mechanism of T4BSS is executed at the transcriptional level by the two-component regulatory system PmrAB (CBU_1277 and CBU_1278). It directly regulates the Dot/Icm secretion system in *L. pneumophila* (McPhee et al., 2003) and *C. burnetii* (van Schaik et al., 2013; Beare et al., 2014). The here observed upregulation of PmrAB in NMI in cell-based experiments corresponds well with a previously published transcriptomic study (Kuley et al., 2015a). Also, the upregulation determined in this study of many T4BSS components in NMI in cell-based experiments fits in with the high PmrAB activity. Seven previously identified T4BSS effector proteins were detected in this study. Only two of them, CvpB and Cig57, have a functional assignment (Chen et al., 2010; Latomanski et al., 2016; Martinez et al., 2016).

RND efflux pumps are associated with excretion of poisonous metal ions and AcrB-like efflux pumps (acrAB) with protection against hydrophobic inhibitors in *E. coli* (Ma et al., 1995). The observed upregulation of RND efflux pumps and uptake of osmolytes (TamAB) during propagation in L-929 and ACCM-D might represent a novel survival mechanism for *C. burnetii* within the CCV.

Contrary to the proposed prominent role of genes in the OPS-associated genomic region, CBU_0679 to CBU_0698 (deleted region in NMII), none of these proteins were observed under axenic or cell-based cultivation in NMI. The apparent absence of these proteins in NMI could be due to a very low expression level under the limit of detection of mass spectrometry, rather than by the absence of expression. These proteomic findings are in concordance with previous studies—none of the proteins were detected after cultivation in VERO cells for NMI and *C. burnetii* Q212 (Papadioti et al., 2011, 2012), respectively (Sandoz et al., 2016b). However, some proteins of the OPS-associated genomic region were observable on the proteomic level during propagation in embryonated hen eggs: CBU_0681, CBU_0682, CBU_0683 (dihydroxystreptose), CBU_0690, and CBU_0691 (virenose) (Flores-Ramirez et al., 2014). This might be due to massive replication of *C. burnetii* during egg propagation and a higher amount of bacterial proteins in the according samples. An improvement of bacterial protein isolation and implementation of more sensitive proteomic methods will enhance the analytical sensitivity. This might allow one to follow regulation of protein expression of OPS-associated genes.

Overall, the data presented here show the successful application of an MS-based LFQ approach for semi-quantitative analysis of *C. burnetii* proteomes under different growth conditions. It was demonstrated that the proteome profiles differ according to growth conditions and growth phase. Furthermore, differences in the response to oxidative stress were shown for *C. burnetii* Nine Mile isolates. Major traits necessary for the intracellular lifestyle, such as proteins putatively involved in the Sec translocation and T4BSS, were detected during axenic cultivation. The approach established in this study allows for complex and sensitive analyses of protein expression profiles in various *C. burnetii* isolates and preparations. It might allow for a future analysis of isolate-specific virulence traits. Although the data presented in this pilot study are promising, their confirmations via other proteomic-based techniques like targeted mass spectrometry or Western blot are needed.

## Data Availability

The mass spectrometry proteomics data have been deposited to the ProteomeXchange Consortium via the PRIDE (Perez-Riverol et al., 2019) partner repository with the dataset identifier PXD013224 (https://www.ebi.ac.uk/pride/archive/projects/PXD013224).

## Author Contributions

JD and KM-S designed the experiments. JD, KM-S, JK, and PP wrote the manuscript. GS participated in the genome quantification of prepared samples. JD, KM-S, and PP prepared the samples. JK conducted the mass spectrometry analyses. BS, AF, JD, MC, and KM-S performed analysis of data. All authors discussed the results and reviewed the manuscript.

### Conflict of Interest Statement

The authors declare that the research was conducted in the absence of any commercial or financial relationships that could be construed as a potential conflict of interest.
