# Supplementary material for: Quantitative Proteome Profiling of Coxiella burnetii Reveals Major Metabolic and Stress Differences Under Axenic and Cell Culture Cultivation
Source: Front Microbiol. 2019 Sep 18;10:2022. doi: 10.3389/fmicb.2019.02022 (PMC6759588; doi:10.3389/fmicb.2019.02022)
Supplement: Supplementary file 1 [file Data_Sheet_1.doc]

Supplementary Material

## S.1.1 DNA isolation and quantification by realtime PCR (qPCR).

Bacterial quantification was carried out as previously described by realtime PCR (qPCR) using the isocitrate dehydrogenase encoding gene (*icd*) as target ^49^.

Briefly, 20 µl bacterial aliquots were lysed in 180 µl of lysis buffer (250 mM Tris, pH 7.5; 10 mM EDTA, 0.1% (w/v) glucose, 0.4% (w/v) lysozyme) with 20 µl of Proteinase K (20 mg/ml) for at least 1 h at 56°C ^50^. DNA was then isolated using the High Pure PCR Template Preparation Kit (Roche) following the recommendations of the supplier. For quantification a plasmid standard was generated by amplification of a 302 bp fragment of the *icd* gene from NMII genomic DNA with the following primer: CoxICD_F1 5`-ATGGATGGAGATCTACGCCG-3` and CoxICD_R1 5`ATTGAATTCCGGCGTAAATATCT-3`. Introduced restrictions sites for *Bgl*II and *Eco*RI are underlined. The obtained PCR product was cloned into pSP72 (Promega) and adjusted to 10^9^ copies/µl based on size of the vector and DNA concentration. Serial dilutions, ranging from 10^6^ to 10^1^ copies/µl, were assessed in duplicate in five independent qPCR runs for standard curve evaluation and compared to a genomic DNA standard (data not shown). All qPCR reactions were performed in a 25 μl format using Maxima Probe/ROX qPCR Master Mix (ThermoFisher Scientific), 300 nM each primer (icd-439F and icd-514R), 100 nM probe (icd-464TM) and 2 µl of plasmid standard or genomic DNA standard as described previously.^49^ The qPCR was carried out on a Mx3000P QPCR Instrument (Agilent Technologies) using the following cycling conditions: 2 min at 50°C, 10 min at 95°C, followed by 40 cycles of 15 s at 95°C and 30 s at 60°C. Data collection and analysis were carried out using the Mx Pro4 software.

## S 1.2C-MS/MS analysis

An UltiMate 3000 RSLCnano system controlled by Chromeleon software (Dionex, USA) was used for chromatography separation. Each sample was loaded onto a PepMap100 C18, 3 µm, 100 Å, 0.075 × 20 mm trap column (Dionex) at 5 µL/min for 5 min. Peptides were separated on a PepMap RSLC C18, 2 µm, 100 Å, 0.075 × 150 mm analytical column (Dionex) by a gradient formed by mobile phase A (0.1% formic acid, FA) and mobile phase B (80% ACN, 0.1% FA), running from 4 to 34% in 68 min, and from 34 to 55% of mobile phase B in 21 min at a flow rate of 0.3 µL/min at 40 °C. Eluted peptides were on-line electrosprayed into Q-Exactive mass spectrometer using a Nanospray Flex ion source (Thermo Scientific, Bremen, Germany). Positive ion full scan MS spectra (*m/z* 350-1650) were acquired using a 1×10^6^ AGC target in the Orbitrap at 70 000 resolution. Top 12 precursors of charge state ≥2 and threshold intensity of 5×10^4^ counts were selected for HCD fragmentation, with a dynamic exclusion window of 60 s. The isolation window of 1.6 Da and normalized CE of 27 was used. Each MS/MS spectrum was acquired at resolution of 17,500, with a 10^5^ AGC target and a maximum 100 ms injection time.

## S 1.3 LFQ - Raw data processing of *C. burnetii* proteins

Raw files acquired in S 1.1 were further analyzed in MaxQuant(Cox and Mann, 2008) (version 1.5.3.30) and the Andromeda was used as search engine (Cox et al., 2011) to search the detected features against the *Coxiella* burnetii (strain RSA 493 / Nine Mile phase I), databases downloaded from Uniprot (April 2^nd^ 2016). Only tryptic peptides that were at least seven amino acids in length with up to two missed cleavages were considered. The initial allowed mass tolerance was set to 4.5 ppm at the MS level and 0.5 Da at the MS/MS level. The oxidation of methionine was set as variable modification and carbamidomethylation of cysteine was set as a fixed modification. A false discovery rate (FDR) of 1 % was imposed for peptide-spectrum matches (PSMs) and protein identification using a target–decoy approach. Relative quantification was performed using the default parameters of the MaxLFQ algorithm (Cox et al., 2014) with the minimum ratio count set to 2.

## S 1.4 LFQ Data analysis of *C. burnetii* proteins

The “proteinGroups.txt” MaxQuant output file was uploaded into Perseus (Cox et al., 2014) (version 1.5.2.6) for further filtering and statistical analysis. Decoy hits, proteins only identified by site, and potential contaminants were removed. Only those protein groups quantified in at least two replicates out of three (for axenic media samples) and at least three out of the whole group for cell-based cultures were considered for further log_2_ transformation of LFQ intensities.

| Sample Nr | Phase of *C. burnetti* | | Medium | Genomic Equivalence [GE] /cell | c (µg/ml)  protein | Nr of identified *C. burnetii* proteins | Nr of identified *C. burnetii* peptides |
| --- | --- | --- | --- | --- | --- | --- | --- |
| 1 | | Phase I | ACCM-D | 1,89E+11 | 50,880 | 1038 | 8674 |
| 2 | | Phase I | ACCM-D | 1,89E+11 | 46,283 | 1004 | 8019 |
| 3 | | Phase I | ACCM-D | 1,89E+11 | 30,878 | 1036 | 8390 |
| 4 | | Phase I | ACCM-2 | 1,00E+11 | 36,998 | 981 | 7720 |
| 5 | | Phase I | ACCM-2 | 1,00E+11 | 42,450 | 1018 | 8600 |
| 6 | | Phase I | ACCM-2 | 2,27E+11 | 45,235 | 1046 | 8878 |
| 7 | | Phase II | ACCM-2 | 3,57E+10 | 43,710 | 1038 | 8367 |
| 8 | | Phase II | ACCM-2 | 3,57E+10 | 32,873 | 997 | 7719 |
| 9 | | Phase II | ACCM-2 | 3,57E+10 | 29,550 | 1018 | 7689 |
| 10 | | Phase II | ACCM-D | 3,41E+10 | 35,978 | 1014 | 7858 |
| 11 | | Phase II | ACCM-D | 3,41E+10 | 35,910 | 1019 | 8363 |
| 12 | | Phase II | ACCM-D | 3,41E+10 | 23,468 | 991 | 7553 |
| 13 | | - | L929 neg control | 1,66E+06 | 1012,050 | - | - |
| 14 | | - | L929 neg control | 5,00E+05 | 345,000 | - | - |
| 15 | | - | L929 neg control | 1,66E+06 | 1206,300 | - | - |
| 16 | | - | L929 neg control | 1,66E+06 | 1335,750 | - | - |
| 17 | | - | L929 neg control | 5,00E+05 | 358,950 | - | - |
| 18 | | - | L929 neg control | 5,00E+05 | 302,850 | - | - |
| 19 | | Phase II | L929 | 3,37E+10 | 190,800 | 659 | 4487 |
| 20 | | Phase II | L929 | 3,47E+09 | 630,450 | 809 | 5899 |
| 21 | | Phase II | L929 | 2,04E+09 | 1021,200 | 914 | 6547 |
| 22 | | Phase II | L929 | 5,41E+09 | 887,100 | 861 | 6710 |
| 23 | | Phase II | L929 | 4,55E+10 | 613,650 | 699 | 5078 |
| 24 | | Phase II | L929 | 1,44E+10 | 1245,900 | 721 | 4758 |
| 25 | | Phase I | L929 | 1,46E+10 | 1796,700 | 881 | 7163 |
| 26 | | Phase I | L929 | 1,13E+10 | 1603,650 | 869 | 6964 |
| 27 | | Phase I | L929 | 1,16E+10 | 1446,450 | 893 | 7180 |
| 28 | | Phase I | L929 | 8,40E+09 | 1193,400 | 822 | 6550 |
| 29 | | Phase I | L929 | 1,70E+10 | 1469,550 | 848 | 6864 |

**Table S1: List of analyzed samples with the Genomic Equivalence [GE] /cell, their protein yields, number of identified proteins and number of identified peptides**

|  |  | **LFQ value (average)** | | | |  | | |
| --- | --- | --- | --- | --- | --- | --- | --- | --- |
|  |  |  |  |  |  |  |  |  |
| **Protein IDs** | **Gene name** | **Phase I ACCMD median** | **Phase I ACCM2 median** | **Phase II ACCM2 median** | **Phase II ACCMD median** | **Peptides** | **MS/MS Count** | **ANOVA q-value** |
| B5QS73 | CBU_0089a | 27,179 | 24,820 | 26,848 | 24,260 | 4 | 22 | 0,0036 |
| B5QS96 | CBU_0516a | 25,177 | 22,874 | 24,151 | 22,748 | 3 | 13 | 0,0036 |
| B5QS99 | CBU_0562a | 27,888 | 25,264 | 27,006 | 23,728 | 7 | 45 | 0,0001 |
| B5QSA6 | cyoD | 22,760 | 26,132 | 22,966 | 23,773 | 2 | 16 | 0,0000 |
| B5QSB9 | CBU_1224a | 28,396 | 26,223 | 27,435 | 25,858 | 1 | 84 | 0,0009 |
| Q45966;B5QSC0 | scvA | 28,829 | 23,313 | 26,794 | 23,289 | 1 | 25 | 0,0000 |
| B5QSC8 | CBU_1379a | 24,278 | 23,911 | 25,506 | 25,358 | 4 | 25 | 0,0003 |
| B5QSE4 | CBU_1634a | 23,852 | 23,915 | 24,059 | 24,530 | 3 | 24 | 0,0102 |
| B5U8P9 | parB.1 | 24,277 | 24,854 | 24,583 | 24,383 | 8 | 38 | 0,0913 |
| B5U8Q0 | CBU_0677 | 22,991 | 23,349 | 28,836 | 29,384 | 13 | 83 | 0,0000 |
| B5U8Q7 | CBU_0700 | 22,339 | 22,504 | 24,094 | 26,735 | 8 | 28 | 0,0001 |
| H7C7C8 | CBU_0703 | 23,745 | 23,723 | 24,616 | 24,877 | 3 | 31 | 0,0103 |
| H7C7C9 | parB.2 | 28,158 | 28,089 | 28,492 | 28,332 | 15 | 148 | 0,0065 |
| H7C7D5 | CBU_0702 | 26,354 | 25,923 | 26,467 | 26,415 | 6 | 48 | 0,0212 |
| H7C7D7 | com1 | 31,732 | 31,806 | 31,485 | 30,916 | 15 | 380 | 0,0015 |
| H7C7D8 | cysQ-2 | 26,603 | 25,716 | 26,334 | 26,492 | 12 | 77 | 0,0007 |
| H7C7D9 | CBU_1670 | 24,432 | 23,927 | 24,262 | 24,322 | 3 | 15 | 0,3764 |
| H7C7E5 | rstB | 23,366 | 22,766 | 23,559 | 23,049 | 1 | 7 | 0,5042 |
| H7C7E7 | CBU_0676 | 22,875 | 23,476 | 30,371 | 30,545 | 17 | 128 | 0,0000 |
| H7C7F0 | CBU_0699 | 24,037 | 23,168 | 23,408 | 23,583 | 5 | 9 | 0,0716 |
| H7C7F1 | CBUA0037 | 26,479 | 27,191 | 27,349 | 27,273 | 12 | 83 | 0,0000 |
| H7C7F3 | icmT | 23,819 | 23,434 | 23,185 | 23,421 | 2 | 15 | 0,0335 |
| H7C7F5 | CBUA0013 | 25,622 | 26,494 | 25,086 | 26,689 | 11 | 52 | 0,0012 |
| H7C7G0 | CBUA0027 | 25,792 | 25,072 | 26,149 | 25,411 | 3 | 35 | 0,0065 |
| H7C7G1 | CBU_0678 | 23,025 | 23,515 | 27,233 | 27,808 | 17 | 96 | 0,0000 |
| H7C7G2 | rfbI | 25,756 | 25,451 | 25,984 | 26,536 | 8 | 43 | 0,0013 |
| O85387 | rplV | 28,320 | 28,953 | 28,955 | 29,027 | 7 | 66 | 0,0334 |
| O85388 | rpsC | 30,096 | 30,407 | 30,612 | 30,797 | 20 | 227 | 0,0004 |
| O87712 | dnaK | 32,341 | 32,824 | 32,502 | 32,605 | 36 | 906 | 0,0237 |
| P0C8S3 | rplL | 29,930 | 30,038 | 30,608 | 30,667 | 6 | 539 | 0,0001 |
| P0C8S4 | rpoB | 32,299 | 32,533 | 32,501 | 32,731 | 74 | 1243 | 0,0004 |
| P18789 | gltA | 29,408 | 29,172 | 29,020 | 29,016 | 14 | 164 | 0,0007 |
| P19421 | groL | 34,602 | 34,575 | 34,581 | 34,521 | 47 | 2852 | 0,9758 |
| P19422 | groS | 32,788 | 32,712 | 33,011 | 32,968 | 9 | 383 | 0,0360 |
| P19685 | sodB | 30,860 | 30,540 | 30,200 | 30,193 | 11 | 157 | 0,0018 |
| P24703 | dapB | 25,863 | 25,350 | 25,901 | 25,665 | 6 | 32 | 0,0036 |
| P39916 | trxB | 27,953 | 28,229 | 27,993 | 28,170 | 11 | 152 | 0,4161 |
| P39917 | lolA | 28,380 | 27,757 | 28,061 | 27,442 | 8 | 120 | 0,0545 |
| P39918 | rarA | 24,804 | 25,137 | 24,975 | 25,584 | 11 | 37 | 0,0032 |
| P39919 | serS | 28,622 | 28,346 | 28,235 | 28,154 | 17 | 154 | 0,0003 |
| P39920 | ftsK | 28,240 | 27,411 | 28,045 | 27,869 | 20 | 149 | 0,0000 |
| P42381 | dnaJ | 26,767 | 27,215 | 27,155 | 27,389 | 14 | 110 | 0,0001 |
| P45648 | rnpA | 23,258 | 23,248 | 23,455 | 23,883 | 2 | 14 | 0,3153 |
| P45650 | yidC | 27,530 | 27,474 | 27,599 | 27,852 | 12 | 90 | 0,0147 |
| P45651 | glyS | 28,295 | 28,409 | 28,136 | 28,156 | 30 | 231 | 0,0085 |
| P45679 | rsmB | 23,249 | 22,960 | 23,480 | 22,862 | 2 | 5 | 0,0755 |
| P45680 | uspA2 | 29,315 | 28,550 | 29,326 | 28,803 | 6 | 130 | 0,0001 |
| P47846 | hemA | 23,019 | 22,461 | 23,418 | 23,365 | 2 | 6 | 0,4055 |
| P47849 | prfA | 26,150 | 26,292 | 26,354 | 25,998 | 11 | 49 | 0,0215 |
| P51053 | sdhB | 28,635 | 28,548 | 28,312 | 28,518 | 8 | 118 | 0,0043 |
| P51054 | sdhA | 30,005 | 29,894 | 29,737 | 29,988 | 20 | 216 | 0,0009 |
| P51055 | sdhC | 23,766 | 25,202 | 24,487 | 22,892 | 1 | 15 | 0,2421 |
| P51056 | sucA | 30,920 | 31,030 | 30,904 | 31,073 | 40 | 546 | 0,0011 |
| P51752 | mip | 30,624 | 30,038 | 30,237 | 29,558 | 13 | 179 | 0,0001 |
| P51836 | era | 26,040 | 25,953 | 25,979 | 25,964 | 6 | 54 | 0,7406 |
| P51837 | rnc | 25,207 | 26,463 | 25,351 | 25,264 | 9 | 25 | 0,0003 |
| P53591 | sucD | 29,130 | 29,192 | 29,148 | 29,333 | 11 | 177 | 0,7523 |
| P53592 | sucC | 30,945 | 31,255 | 31,142 | 31,186 | 19 | 310 | 0,0151 |
| P59650 | CBU_0721 | 27,002 | 27,578 | 27,701 | 28,168 | 12 | 82 | 0,0006 |
| P59753 | rpsM | 28,772 | 29,495 | 29,391 | 29,600 | 8 | 86 | 0,0087 |
| P94612 | mnmE | 26,240 | 26,929 | 26,612 | 26,549 | 18 | 93 | 0,0005 |
| P94613 | mnmG | 24,520 | 25,833 | 24,955 | 25,044 | 8 | 43 | 0,0004 |
| P94616 | glyQ | 26,617 | 26,566 | 26,404 | 26,568 | 6 | 57 | 0,0165 |
| Q45885 | djlA | 23,432 | 24,395 | 23,958 | 23,882 | 4 | 13 | 0,0146 |
| Q45918 | pyrE | 28,472 | 28,864 | 28,099 | 27,954 | 9 | 81 | 0,0000 |
| Q45920 | dut | 26,107 | 25,651 | 26,796 | 26,590 | 4 | 37 | 0,0000 |
| Q4AAX7 | truB | 25,148 | 25,272 | 25,215 | 25,306 | 5 | 42 | 0,2720 |
| Q4AAX8 | CBU_1059 | 26,028 | 26,691 | 26,282 | 26,196 | 11 | 58 | 0,1192 |
| Q4AAX9 | truA | 24,182 | 23,917 | 24,174 | 23,462 | 6 | 13 | 0,2421 |
| Q4AAY0 | rluD | 25,708 | 25,500 | 25,537 | 25,783 | 10 | 62 | 0,2746 |
| Q4AAY1 | rluC | 23,753 | 23,749 | 23,870 | 23,896 | 5 | 16 | 0,5658 |
| Q81ZL2 | smc | 29,271 | 28,917 | 29,189 | 29,289 | 39 | 295 | 0,0017 |
| Q820B1 | pabA | 23,898 | 24,780 | 24,216 | 24,425 | 4 | 22 | 0,0113 |
| Q820B3 | rfbA | 26,607 | 26,287 | 26,904 | 27,295 | 15 | 75 | 0,0016 |
| Q820B4 | lnt | 24,951 | 24,979 | 24,519 | 24,895 | 4 | 34 | 0,0336 |
| Q820B5 | ubiG | 27,830 | 27,393 | 27,495 | 27,576 | 14 | 110 | 0,0006 |
| Q820B6 | CBU_0175 | 23,847 | 24,671 | 24,022 | 24,513 | 5 | 21 | 0,0100 |
| Q820V9 | cca | 25,734 | 25,222 | 25,533 | 25,449 | 11 | 73 | 0,0021 |
| Q820W0 | rsmI | 25,396 | 25,747 | 25,502 | 25,133 | 11 | 24 | 0,0047 |
| Q820W1 | CBU_1482 | 28,780 | 28,518 | 28,892 | 28,532 | 13 | 128 | 0,0067 |
| Q820W2 | mnmA | 25,008 | 24,544 | 24,729 | 25,098 | 8 | 42 | 0,0059 |
| Q820W3 | miaA | 24,130 | 23,809 | 23,891 | 23,935 | 4 | 24 | 0,4319 |
| Q820W4 | CBU_0847 | 25,020 | 24,850 | 25,185 | 25,066 | 6 | 42 | 0,0201 |
| Q820W5 | CBU_0744 | 25,325 | 25,655 | 25,160 | 24,919 | 7 | 56 | 0,0002 |
| Q820W6 | lpxA | 27,590 | 27,414 | 27,253 | 27,350 | 6 | 67 | 0,0106 |
| Q820W7 | fabZ | 28,223 | 28,059 | 27,830 | 27,837 | 8 | 62 | 0,0004 |
| Q820W8 | CBU_0610 | 27,808 | 28,204 | 27,738 | 27,963 | 9 | 98 | 0,0020 |
| Q820W9 | fabG | 29,159 | 28,863 | 29,103 | 29,038 | 7 | 124 | 0,0229 |
| Q820X0 | fabH | 28,600 | 28,955 | 28,796 | 28,834 | 7 | 131 | 0,0958 |
| Q820X1 | trmD | 24,591 | 25,435 | 25,203 | 25,367 | 8 | 36 | 0,0018 |
| Q820X2 | spoT | 28,860 | 28,658 | 28,495 | 28,306 | 30 | 213 | 0,0006 |
| Q820X3 | murG | 25,665 | 25,751 | 25,723 | 25,539 | 8 | 65 | 0,3488 |
| Q820X4 | CBU_0038 | 26,879 | 27,388 | 27,142 | 27,178 | 9 | 64 | 0,0124 |
| Q820X5 | fabA | 25,397 | 26,267 | 26,201 | 26,209 | 6 | 41 | 0,0004 |
| Q820X6 | CBU_0036 | 24,238 | 25,474 | 23,740 | 24,822 | 2 | 26 | 0,0032 |
| Q83A05 | CBUA0023 | 26,238 | 26,783 | 25,868 | 27,148 | 10 | 59 | 0,0001 |
| Q83A13 | CBUA0010 | 25,491 | 25,413 | 25,429 | 25,509 | 12 | 51 | 0,9474 |
| Q83A16 | pyrB | 26,849 | 27,324 | 26,857 | 26,809 | 10 | 79 | 0,0002 |
| Q83A18 | CBU_2093 | 22,929 | 24,914 | 23,692 | 23,700 | 4 | 18 | 0,0019 |
| Q83A19 | pckA | 29,981 | 30,255 | 30,012 | 30,067 | 22 | 309 | 0,0018 |
| Q83A20 | CBU_2091 | 26,217 | 26,513 | 26,117 | 26,079 | 5 | 34 | 0,0005 |
| Q83A21 | proC | 27,609 | 27,730 | 27,494 | 27,545 | 10 | 98 | 0,0107 |
| Q83A22 | CBU_2089 | 25,999 | 26,284 | 26,354 | 26,043 | 2 | 22 | 0,0699 |
| Q83A23 | CBU_2088 | 22,973 | 24,807 | 25,177 | 24,492 | 3 | 24 | 0,0095 |
| Q83A24 | trx | 28,647 | 28,214 | 28,484 | 28,451 | 4 | 90 | 0,3424 |
| Q83A25 | rho | 30,258 | 30,760 | 30,766 | 30,781 | 23 | 372 | 0,0021 |
| Q83A26 | CBU_2085 | 25,345 | 25,811 | 25,362 | 25,437 | 4 | 31 | 0,0137 |
| Q83A28 | queC | 23,040 | 22,923 | 23,639 | 23,429 | 4 | 13 | 0,1260 |
| Q83A29 | CBU_2082 | 23,747 | 23,524 | 23,523 | 22,733 | 3 | 11 | 0,1287 |
| Q83A30 | hemY | 29,244 | 29,022 | 28,889 | 28,835 | 20 | 213 | 0,0016 |
| Q83A31 | CBU_2080 | 28,572 | 28,344 | 28,310 | 28,023 | 13 | 157 | 0,0007 |
| Q83A33 | CBU_2078 | 25,038 | 24,744 | 25,004 | 24,774 | 7 | 34 | 0,0209 |
| Q83A34 | hemD | 24,671 | 24,764 | 24,622 | 24,117 | 7 | 28 | 0,0505 |
| Q83A35 | CBU_2076 | 26,779 | 26,907 | 27,089 | 26,733 | 5 | 33 | 0,4385 |
| Q83A36 | aroD | 24,862 | 25,069 | 25,095 | 24,957 | 3 | 25 | 0,0291 |
| Q83A37 | hemC | 26,279 | 26,426 | 26,571 | 26,275 | 7 | 48 | 0,0291 |
| Q83A38 | CBU_2073 | 26,252 | 26,059 | 26,181 | 25,696 | 3 | 32 | 0,0023 |
| Q83A39 | CBU_2072 | 25,498 | 24,811 | 25,553 | 24,951 | 4 | 26 | 0,1131 |
| Q83A41 | CBU_2070 | 27,499 | 27,290 | 27,570 | 26,882 | 6 | 39 | 0,0011 |
| Q83A42 | tdk | 24,852 | 24,844 | 24,970 | 24,502 | 5 | 27 | 0,0552 |
| Q83A43 | CBU_2068 | 24,880 | 24,641 | 24,959 | 24,288 | 2 | 13 | 0,1491 |
| Q83A53 | CBU_2057 | 23,400 | 24,068 | 23,554 | 23,896 | 2 | 14 | 0,2190 |
| Q83A56 | uvrD | 25,653 | 27,294 | 27,047 | 27,756 | 19 | 116 | 0,0001 |
| Q83A61 | trpS | 26,306 | 26,307 | 26,472 | 26,537 | 11 | 85 | 0,0379 |
| Q83A62 | metE | 26,548 | 27,560 | 27,341 | 26,997 | 20 | 101 | 0,0006 |
| Q83A77 | ahcY | 29,748 | 29,746 | 29,967 | 30,202 | 22 | 217 | 0,0015 |
| Q83A78 | metK | 29,933 | 30,195 | 30,567 | 30,612 | 19 | 309 | 0,0001 |
| Q83A79 | CBU_2029 | 30,367 | 28,404 | 28,276 | 28,587 | 8 | 102 | 0,0001 |
| Q83A83 | metC | 26,686 | 27,308 | 27,482 | 27,504 | 12 | 104 | 0,0002 |
| Q83A84 | CBU_2024 | 26,567 | 27,245 | 27,049 | 27,023 | 8 | 91 | 0,0006 |
| Q83A85 | CBU_2023 | 26,110 | 25,752 | 26,166 | 25,888 | 6 | 38 | 0,0030 |
| Q83A86 | CBU_2021 | 23,515 | 23,585 | 23,290 | 23,419 | 6 | 9 | 0,8950 |
| Q83A88 | ubiB | 27,295 | 27,423 | 27,557 | 27,331 | 16 | 104 | 0,0080 |
| Q83A90 | ubiE | 26,927 | 27,149 | 26,954 | 27,278 | 9 | 67 | 0,0021 |
| Q83A91 | CBU_2016 | 23,835 | 23,239 | 23,429 | 22,777 | 4 | 13 | 0,1049 |
| Q83A94 | hslU | 30,387 | 31,117 | 30,795 | 31,034 | 31 | 368 | 0,0004 |
| Q83A95 | hslV | 27,832 | 28,894 | 27,964 | 28,294 | 7 | 108 | 0,0004 |
| Q83A97 | CBU_2009 | 29,535 | 29,552 | 28,914 | 28,977 | 20 | 179 | 0,0018 |
| Q83A98 | argS | 27,636 | 27,858 | 27,533 | 27,532 | 15 | 142 | 0,0040 |
| Q83A99 | CBU_2007 | 23,132 | 22,746 | 22,586 | 23,870 | 4 | 6 | 0,0123 |
| Q83AA0 | CBU_2006 | 24,330 | 25,563 | 25,355 | 26,107 | 8 | 30 | 0,0001 |
| Q83AA1 | CBU_2004 | 26,187 | 26,619 | 26,408 | 26,436 | 10 | 71 | 0,0036 |
| Q83AA3 | purE | 26,559 | 26,947 | 26,896 | 26,784 | 4 | 58 | 0,0474 |
| Q83AA5 | topA | 28,091 | 28,143 | 28,400 | 28,209 | 36 | 224 | 0,0016 |
| Q83AA6 | smg | 23,081 | 22,803 | 23,049 | 22,774 | 2 | 6 | 0,4465 |
| Q83AA8 | fmt | 26,352 | 26,803 | 26,527 | 26,484 | 10 | 59 | 0,0373 |
| Q83AA9 | CBU_1996 | 26,609 | 26,823 | 27,236 | 27,066 | 9 | 75 | 0,0021 |
| Q83AB2 | folA | 25,866 | 25,827 | 25,946 | 25,712 | 4 | 24 | 0,7230 |
| Q83AB7 | apaH | 24,305 | 23,962 | 24,508 | 24,145 | 4 | 34 | 0,2270 |
| Q83AB8 | sppA | 28,493 | 28,275 | 28,627 | 28,509 | 11 | 171 | 0,0074 |
| Q83AB9 | CBU_1985 | 23,394 | 23,239 | 24,329 | 23,171 | 4 | 6 | 0,0132 |
| Q83AC0 | CBU_1984 | 23,315 | 23,302 | 24,750 | 23,892 | 3 | 12 | 0,0015 |
| Q83AC1 | uspA1 | 27,826 | 27,475 | 27,784 | 27,495 | 6 | 96 | 0,0458 |
| Q83AC2 | rsmA | 26,678 | 26,417 | 26,327 | 25,814 | 11 | 58 | 0,0012 |
| Q83AC3 | pdxA | 26,355 | 26,788 | 26,414 | 25,927 | 9 | 55 | 0,0015 |
| Q83AC4 | CBU_1980 | 30,170 | 30,016 | 29,874 | 29,656 | 15 | 173 | 0,0006 |
| Q83AC6 | lptD | 25,472 | 25,787 | 25,800 | 25,621 | 11 | 73 | 0,1856 |
| Q83AC7 | CBU_1977 | 24,639 | 24,607 | 24,266 | 24,746 | 9 | 39 | 0,0803 |
| Q83AC8 | CBU_1976 | 25,072 | 25,752 | 26,389 | 26,568 | 10 | 44 | 0,0000 |
| Q83AC9 | CBU_1975 | 27,616 | 27,743 | 27,601 | 27,319 | 6 | 87 | 0,0383 |
| Q83AD4 | dapF | 28,272 | 28,031 | 28,107 | 27,942 | 12 | 111 | 0,0181 |
| Q83AD5 | dksA | 27,395 | 27,228 | 27,871 | 27,637 | 6 | 42 | 0,0141 |
| Q83AD6 | folB | 23,229 | 24,154 | 23,854 | 23,636 | 3 | 15 | 0,0296 |
| Q83AD8 | prmC | 24,850 | 25,056 | 24,873 | 24,837 | 10 | 48 | 0,5053 |
| Q83AE4 | pntB | 24,888 | 23,268 | 25,194 | 25,257 | 3 | 20 | 0,0241 |
| Q83AE6 | pntAA | 27,558 | 27,272 | 27,886 | 27,702 | 18 | 112 | 0,0026 |
| Q83AE7 | CBU_1954 | 26,410 | 24,017 | 26,800 | 23,824 | 8 | 54 | 0,0001 |
| Q83AE9 | CBU_1952 | 23,190 | 22,666 | 23,155 | 22,859 | 4 | 6 | 0,3270 |
| Q83AF3 | glmU | 28,389 | 28,400 | 28,335 | 28,504 | 17 | 212 | 0,0060 |
| Q83AF4 | atpC | 25,225 | 25,460 | 25,256 | 25,321 | 2 | 22 | 0,9001 |
| Q83AF5 | atpD | 30,159 | 30,780 | 30,225 | 30,336 | 28 | 318 | 0,0006 |
| Q83AF6 | atpG | 27,811 | 28,261 | 27,812 | 28,084 | 10 | 132 | 0,0000 |
| Q83AF7 | atpA | 30,834 | 31,268 | 30,790 | 31,032 | 29 | 367 | 0,0001 |
| Q83AF8 | atpH | 25,774 | 26,872 | 26,387 | 26,209 | 8 | 86 | 0,0136 |
| Q83AF9 | atpF | 28,101 | 28,606 | 28,025 | 28,331 | 10 | 111 | 0,0001 |
| Q83AG0 | atpE | 28,799 | 28,577 | 28,523 | 28,802 | 2 | 93 | 0,5065 |
| Q83AG1 | atpB | 24,551 | 24,292 | 24,679 | 24,870 | 1 | 13 | 0,7525 |
| Q83AG6 | ku | 23,681 | 23,386 | 23,569 | 22,826 | 2 | 4 | 0,1290 |
| Q83AH1 | CBU_1928 | 25,534 | 25,164 | 26,102 | 25,463 | 13 | 68 | 0,0017 |
| Q83AH2 | parB | 26,863 | 27,250 | 27,368 | 27,166 | 14 | 139 | 0,0152 |
| Q83AH3 | parA | 25,649 | 25,495 | 25,694 | 25,380 | 8 | 51 | 0,0291 |
| Q83AH9 | CBU_1907 | 22,797 | 24,136 | 23,236 | 22,944 | 5 | 21 | 0,0251 |
| Q83AI3 | ftsY | 25,708 | 25,906 | 25,591 | 25,504 | 8 | 44 | 0,0032 |
| Q83AI4 | CBU_1902 | 28,763 | 28,774 | 28,635 | 28,131 | 14 | 144 | 0,0003 |
| Q83AI5 | CBU_1901 | 29,191 | 29,126 | 29,026 | 28,503 | 18 | 217 | 0,0001 |
| Q83AI9 | CBU_1896 | 23,409 | 23,294 | 23,694 | 23,717 | 2 | 15 | 0,1658 |
| Q83AJ2 | aroB | 25,743 | 25,712 | 25,660 | 25,313 | 10 | 78 | 0,1282 |
| Q83AJ8 | ponA | 27,588 | 27,529 | 27,369 | 27,216 | 18 | 149 | 0,0116 |
| Q83AK2 | queD | 26,734 | 26,404 | 26,884 | 26,505 | 8 | 78 | 0,0083 |
| Q83AK3 | hemL | 26,416 | 26,563 | 26,553 | 26,643 | 11 | 55 | 0,5532 |
| Q83AK5 | CBU_1880 | 24,085 | 23,650 | 23,998 | 24,442 | 1 | 12 | 0,1201 |
| Q83AK6 | def2 | 25,579 | 26,408 | 25,972 | 25,882 | 4 | 42 | 0,0005 |
| Q83AK7 | erpA | 23,190 | 23,752 | 23,622 | 24,379 | 2 | 22 | 0,0003 |
| Q83AK8 | CBU_1877 | 26,822 | 26,550 | 26,714 | 26,498 | 13 | 124 | 0,0010 |
| Q83AK9 | rsmJ | 23,377 | 23,047 | 23,950 | 23,962 | 5 | 13 | 0,0559 |
| Q83AL0 | gshB | 24,294 | 24,515 | 23,801 | 23,484 | 5 | 5 | 0,2463 |
| Q83AL1 | CBU_1874 | 28,589 | 29,165 | 28,348 | 28,428 | 17 | 168 | 0,0006 |
| Q83AL2 | rpe | 27,267 | 27,219 | 27,405 | 27,293 | 10 | 72 | 0,4720 |
| Q83AL3 | coq7 | 24,451 | 24,383 | 24,724 | 24,658 | 4 | 25 | 0,0115 |
| Q83AL4 | CBU_1869 | 27,443 | 26,444 | 27,568 | 26,411 | 9 | 76 | 0,0004 |
| Q83AL7 | parC | 28,758 | 28,843 | 28,677 | 28,648 | 29 | 212 | 0,2779 |
| Q83AL8 | CBU_1865 | 26,900 | 26,887 | 26,741 | 26,449 | 6 | 37 | 0,0123 |
| Q83AM0 | CBU_1863 | 23,719 | 24,260 | 23,427 | 26,021 | 12 | 40 | 0,0005 |
| Q83AM1 | CBU_1862 | 24,719 | 25,723 | 25,429 | 25,636 | 9 | 37 | 0,0001 |
| Q83AM7 | CBU_1856 | 25,286 | 24,316 | 24,805 | 24,940 | 4 | 19 | 0,0001 |
| Q83AN2 | CBU_1851 | 24,528 | 25,095 | 25,007 | 24,899 | 5 | 33 | 0,0968 |
| Q83AN5 | CBU_1847 | 27,206 | 25,130 | 27,366 | 26,136 | 3 | 25 | 0,3962 |
| Q83AN9 | ychF | 26,914 | 26,878 | 26,954 | 27,187 | 13 | 95 | 0,0416 |
| Q83AP0 | pth | 26,147 | 25,780 | 26,335 | 26,292 | 9 | 44 | 0,0018 |
| Q83AP1 | rplY | 28,725 | 29,058 | 29,377 | 29,410 | 10 | 118 | 0,0006 |
| Q83AP2 | CBU_1839 | 24,187 | 23,064 | 23,474 | 24,410 | 6 | 18 | 0,0009 |
| Q83AP3 | rfbC | 26,887 | 26,759 | 27,316 | 27,250 | 7 | 51 | 0,0006 |
| Q83AP4 | CBU_1837 | 28,685 | 29,056 | 29,133 | 29,359 | 15 | 141 | 0,0000 |
| Q83AP5 | CBU_1836 | 29,542 | 29,418 | 29,891 | 30,053 | 13 | 126 | 0,0008 |
| Q83AP6 | CBU_1835 | 28,968 | 29,204 | 29,358 | 29,368 | 23 | 244 | 0,0156 |
| Q83AP7 | rmlA | 24,932 | 25,344 | 26,098 | 26,040 | 9 | 44 | 0,0000 |
| Q83AQ1 | prs | 28,163 | 28,628 | 28,322 | 28,391 | 14 | 146 | 0,0023 |
| Q83AQ2 | lolB | 25,951 | 25,510 | 26,056 | 25,995 | 5 | 52 | 0,0007 |
| Q83AQ4 | psd | 25,975 | 26,207 | 25,839 | 26,269 | 6 | 44 | 0,0215 |
| Q83AQ8 | sodC | 24,452 | 27,525 | 26,471 | 23,856 | 6 | 39 | 0,0005 |
| Q83AQ9 | CBU_1821 | 24,821 | 24,800 | 24,682 | 24,472 | 4 | 26 | 0,0476 |
| Q83AR0 | CBU_1820 | 26,012 | 25,598 | 25,920 | 25,733 | 6 | 51 | 0,0296 |
| Q83AR3 | CBU_1817 | 24,162 | 24,436 | 24,861 | 25,075 | 3 | 20 | 0,0868 |
| Q83AR4 | efp | 28,168 | 27,636 | 28,434 | 28,356 | 4 | 36 | 0,0000 |
| Q83AR5 | priA | 22,920 | 23,339 | 23,273 | 23,121 | 6 | 16 | 0,4200 |
| Q83AR6 | CBU_1814 | 24,127 | 22,819 | 24,203 | 23,027 | 3 | 19 | 0,0011 |
| Q83AR8 | pdxB | 23,835 | 23,813 | 23,901 | 23,657 | 4 | 39 | 0,2579 |
| Q83AR9 | CBU_1811 | 23,622 | 23,260 | 23,401 | 23,345 | 5 | 15 | 0,1883 |
| Q83AS0 | macA | 26,942 | 26,688 | 26,760 | 26,649 | 12 | 92 | 0,0432 |
| Q83AT2 | CBU_1798 | 25,235 | 25,568 | 25,034 | 25,022 | 7 | 31 | 0,0839 |
| Q83AT3 | CBU_1797 | 23,901 | 25,076 | 24,257 | 23,833 | 2 | 13 | 0,0128 |
| Q83AT5 | polI | 27,865 | 28,415 | 28,054 | 27,848 | 31 | 247 | 0,0006 |
| Q83AU0 | CBU_1789 | 28,265 | 27,638 | 27,410 | 27,838 | 14 | 117 | 0,0001 |
| Q83AU2 | glmS | 28,303 | 28,262 | 28,064 | 28,218 | 25 | 178 | 0,5543 |
| Q83AU3 | CBU_1786 | 23,162 | 23,053 | 22,956 | 23,095 | 5 | 13 | 0,9738 |
| Q83AU4 | tkt | 28,976 | 29,179 | 29,118 | 29,289 | 21 | 212 | 0,0058 |
| Q83AU5 | gap | 31,311 | 31,576 | 31,378 | 31,444 | 21 | 320 | 0,0962 |
| Q83AU6 | pgk | 28,588 | 28,808 | 28,834 | 28,898 | 13 | 206 | 0,2086 |
| Q83AU7 | pyk | 28,973 | 29,394 | 29,325 | 29,238 | 17 | 185 | 0,0062 |
| Q83AV0 | fbaA | 30,732 | 30,381 | 30,680 | 30,558 | 17 | 233 | 0,0012 |
| Q83AV6 | engB | 27,845 | 28,349 | 28,031 | 28,062 | 10 | 99 | 0,0005 |
| Q83AV7 | CBU_1771 | 24,489 | 24,487 | 24,697 | 24,613 | 4 | 18 | 0,7969 |
| Q83AV8 | CBU_1770 | 28,115 | 28,520 | 28,111 | 28,225 | 15 | 166 | 0,0020 |
| Q83AW2 | feoB | 25,656 | 25,765 | 25,575 | 25,618 | 14 | 62 | 0,7231 |
| Q83AW6 | CBU_1762 | 26,436 | 25,132 | 25,458 | 23,725 | 2 | 9 | 0,1553 |
| Q83AW7 | CBU_1761 | 27,273 | 26,828 | 26,907 | 26,040 | 20 | 101 | 0,0000 |
| Q83AX3 | CBU_1754 | 27,560 | 27,511 | 27,284 | 27,210 | 8 | 116 | 0,0068 |
| Q83AX4 | CBU_1753 | 23,412 | 23,537 | 23,050 | 23,769 | 3 | 10 | 0,1710 |
| Q83AX5 | CBU_1752 | 29,470 | 28,747 | 29,138 | 29,782 | 26 | 283 | 0,0008 |
| Q83AX6 | CBU_1751 | 29,237 | 28,873 | 29,185 | 29,567 | 28 | 258 | 0,0001 |
| Q83AX8 | rplM | 28,294 | 28,957 | 28,535 | 28,780 | 6 | 93 | 0,0017 |
| Q83AX9 | rpsI | 27,727 | 28,438 | 28,561 | 28,567 | 4 | 83 | 0,0000 |
| Q83AY0 | sspA | 28,369 | 28,556 | 28,645 | 28,562 | 9 | 142 | 0,0191 |
| Q83AY1 | sspB | 23,804 | 24,905 | 24,601 | 25,245 | 4 | 19 | 0,0278 |
| Q83AY2 | CBU_1745 | 26,140 | 25,349 | 25,792 | 25,314 | 4 | 44 | 0,0005 |
| Q83AY3 | CBU_1744 | 27,820 | 27,621 | 27,732 | 27,486 | 8 | 91 | 0,0038 |
| Q83AY4 | gmhA | 26,143 | 26,562 | 26,220 | 26,050 | 8 | 60 | 0,0166 |
| Q83AY6 | CBU_1741 | 28,311 | 27,923 | 27,961 | 28,078 | 13 | 146 | 0,0009 |
| Q83AY8 | hipB | 24,655 | 24,124 | 23,945 | 23,489 | 1 | 9 | 0,5530 |
| Q83AY9 | purN | 28,378 | 28,382 | 28,326 | 28,493 | 11 | 62 | 0,8538 |
| Q83AZ0 | purM | 27,080 | 27,325 | 27,184 | 27,046 | 9 | 58 | 0,1729 |
| Q83AZ1 | CBU_1735 | 26,560 | 25,428 | 26,684 | 25,726 | 5 | 30 | 0,0131 |
| Q83AZ2 | CBU_1734 | 26,853 | 26,604 | 26,933 | 26,721 | 8 | 54 | 0,1289 |
| Q83AZ4 | CBU_1732 | 25,577 | 27,496 | 26,618 | 26,719 | 14 | 76 | 0,0000 |
| Q83AZ5 | CBU_1730 | 24,922 | 25,206 | 25,382 | 25,208 | 6 | 37 | 0,3814 |
| Q83AZ6 | hemF | 24,804 | 25,157 | 24,479 | 24,622 | 8 | 31 | 0,0033 |
| Q83AZ8 | arcB | 27,191 | 27,661 | 27,709 | 27,644 | 14 | 111 | 0,0085 |
| Q83AZ9 | accC | 27,909 | 27,952 | 28,230 | 28,449 | 13 | 102 | 0,0001 |
| Q83B00 | accB | 27,342 | 27,049 | 27,627 | 27,623 | 6 | 86 | 0,0085 |
| Q83B02 | dsbD | 27,227 | 26,884 | 26,960 | 27,002 | 10 | 74 | 0,0575 |
| Q83B04 | CBU_1721 | 24,882 | 24,701 | 24,449 | 24,221 | 5 | 21 | 0,0227 |
| Q83B05 | acnA | 30,372 | 30,649 | 30,292 | 30,352 | 34 | 393 | 0,0003 |
| Q83B06 | gcvT | 27,519 | 27,483 | 27,447 | 27,547 | 16 | 100 | 0,5263 |
| Q83B07 | gcvH | 28,465 | 28,414 | 28,521 | 28,560 | 6 | 82 | 0,9115 |
| Q83B08 | gcvPA | 26,992 | 27,402 | 27,350 | 27,296 | 9 | 97 | 0,0093 |
| Q83B09 | gcvPB | 27,773 | 28,338 | 27,986 | 28,040 | 17 | 134 | 0,0010 |
| Q83B14 | CBU_1706 | 32,039 | 32,633 | 32,239 | 32,317 | 14 | 406 | 0,0000 |
| Q83B15 | CBU_1705 | 28,372 | 26,044 | 28,347 | 27,455 | 4 | 36 | 0,1131 |
| Q83B16 | rnt | 22,829 | 23,957 | 23,420 | 23,402 | 4 | 16 | 0,0064 |
| Q83B17 | pyrC | 26,729 | 26,680 | 26,877 | 26,743 | 11 | 81 | 0,0011 |
| Q83B20 | CBU_1699 | 23,252 | 22,730 | 23,082 | 22,576 | 3 | 6 | 0,1624 |
| Q83B21 | CBU_1698 | 24,967 | 24,629 | 24,590 | 24,531 | 8 | 41 | 0,0008 |
| Q83B24 | metG | 27,096 | 27,409 | 27,368 | 27,409 | 18 | 116 | 0,0189 |
| Q83B28 | CBU_1691 | 25,404 | 24,490 | 24,421 | 23,432 | 6 | 21 | 0,0006 |
| Q83B30 | apbC | 24,758 | 24,920 | 24,518 | 25,055 | 4 | 20 | 0,3309 |
| Q83B31 | dcd | 26,721 | 26,901 | 26,827 | 26,916 | 6 | 47 | 0,5539 |
| Q83B32 | CBU_1686 | 26,444 | 26,174 | 25,072 | 26,902 | 12 | 52 | 0,0095 |
| Q83B33 | CBU_1685 | 23,772 | 23,541 | 23,125 | 23,539 | 4 | 11 | 0,1848 |
| Q83B36 | pyrG | 28,692 | 28,709 | 28,631 | 28,728 | 20 | 189 | 0,4060 |
| Q83B40 | speG | 27,432 | 26,895 | 26,757 | 26,113 | 6 | 56 | 0,0017 |
| Q83B41 | CBU_1677 | 27,706 | 23,313 | 26,321 | 22,946 | 9 | 40 | 0,0000 |
| Q83B43 | kdsA | 26,618 | 26,557 | 26,538 | 26,842 | 7 | 46 | 0,0239 |
| Q83B44 | eno | 28,505 | 28,404 | 28,648 | 28,499 | 15 | 170 | 0,0299 |
| Q83B51 | CBU_1664 | 28,437 | 28,536 | 28,847 | 28,737 | 7 | 127 | 0,0016 |
| Q83B54 | rfaF | 27,592 | 27,065 | 26,584 | 27,400 | 10 | 80 | 0,0000 |
| Q83B56 | CBU_1659 | 23,296 | 23,067 | 24,878 | 23,245 | 3 | 17 | 0,0044 |
| Q83B57 | CBU_1658 | 24,158 | 24,567 | 24,581 | 24,601 | 8 | 33 | 0,0329 |
| Q83B58 | CBU_1657 | 25,001 | 25,598 | 25,539 | 25,722 | 4 | 27 | 0,0012 |
| Q83B60 | hldE | 27,350 | 27,246 | 27,529 | 28,017 | 20 | 104 | 0,0036 |
| Q83B63 | icmX | 31,096 | 30,007 | 30,032 | 30,464 | 15 | 271 | 0,0000 |
| Q83B64 | CBU_1651 | 28,321 | 27,478 | 27,181 | 27,386 | 4 | 89 | 0,0000 |
| Q83B65 | icmW | 26,181 | 26,504 | 26,214 | 26,503 | 3 | 32 | 0,0009 |
| Q83B66 | icmV | 26,632 | 25,470 | 25,969 | 26,789 | 5 | 51 | 0,0001 |
| Q83B67 | dotaA | 30,211 | 27,102 | 29,967 | 30,346 | 10 | 177 | 0,0000 |
| Q83B69 | CBU_1646 | 22,731 | 22,377 | 22,771 | 22,644 | 1 | 18 | 0,4632 |
| Q83B70 | dotB | 28,829 | 27,991 | 28,182 | 28,298 | 18 | 179 | 0,0001 |
| Q83B71 | dotC | 28,972 | 28,412 | 28,554 | 28,653 | 12 | 174 | 0,0006 |
| Q83B72 | dotD | 28,463 | 27,719 | 28,002 | 28,295 | 6 | 58 | 0,0001 |
| Q83B73 | icmS | 25,129 | 26,054 | 25,396 | 25,627 | 4 | 31 | 0,0017 |
| Q83B75 | CBU_1638 | 26,311 | 25,843 | 26,242 | 25,880 | 8 | 42 | 0,0033 |
| Q83B79 | icmQ | 27,335 | 26,849 | 27,008 | 27,528 | 8 | 64 | 0,0004 |
| Q83B80 | icmP | 25,882 | 26,728 | 26,427 | 26,935 | 11 | 80 | 0,0001 |
| Q83B81 | icmO | 26,833 | 27,109 | 27,260 | 27,620 | 19 | 115 | 0,0004 |
| Q83B82 | icmN | 27,604 | 26,579 | 26,864 | 27,017 | 9 | 83 | 0,0036 |
| Q83B83 | icmL.2 | 28,220 | 27,277 | 27,694 | 27,721 | 8 | 79 | 0,0004 |
| Q83B84 | icmL.1 | 27,106 | 26,548 | 26,313 | 26,513 | 8 | 61 | 0,0001 |
| Q83B85 | icmK | 28,486 | 27,944 | 28,089 | 28,218 | 10 | 109 | 0,0015 |
| Q83B86 | icmE | 31,896 | 31,090 | 31,555 | 31,647 | 50 | 614 | 0,0018 |
| Q83B87 | icmG | 28,303 | 28,233 | 27,965 | 28,380 | 11 | 104 | 0,0020 |
| Q83B88 | icmC | 23,754 | 23,226 | 23,394 | 23,793 | 2 | 13 | 0,0094 |
| Q83B90 | icmJ | 26,078 | 26,037 | 25,855 | 26,270 | 6 | 43 | 0,1289 |
| Q83B91 | icmB | 29,397 | 28,917 | 28,713 | 28,975 | 32 | 254 | 0,0003 |
| Q83BA9 | CBU_1603 | 23,806 | 23,348 | 23,077 | 23,344 | 4 | 10 | 0,0033 |
| Q83BB0 | rimK | 24,597 | 24,831 | 23,822 | 22,897 | 7 | 28 | 0,0003 |
| Q83BB2 | CBU_1600 | 24,495 | 22,804 | 25,833 | 26,191 | 3 | 18 | 0,0000 |
| Q83BB6 | rpoD | 29,063 | 29,058 | 29,282 | 29,256 | 19 | 181 | 0,0397 |
| Q83BB7 | dnaG | 26,003 | 27,009 | 27,151 | 27,208 | 19 | 103 | 0,0000 |
| Q83BB8 | CBU_1594 | 27,440 | 26,979 | 27,401 | 27,431 | 3 | 32 | 0,0803 |
| Q83BC1 | nhaP.2 | 22,711 | 23,885 | 23,217 | 22,066 | 2 | 4 | 0,1047 |
| Q83BC2 | CBU_1589 | 28,173 | 23,308 | 28,290 | 23,784 | 2 | 21 | 0,0000 |
| Q83BC6 | shaD | 24,664 | 23,504 | 24,106 | 24,149 | 2 | 16 | 0,1406 |
| Q83BC9 | shaG | 24,619 | 23,751 | 23,533 | 24,658 | 2 | 17 | 0,0074 |
| Q83BD1 | CBU_1580 | 23,209 | 24,579 | 24,188 | 24,513 | 4 | 28 | 0,0006 |
| Q83BD2 | CBU_1579 | 26,667 | 26,387 | 26,745 | 26,593 | 6 | 47 | 0,0743 |
| Q83BD4 | CBU_1577 | 25,802 | 26,200 | 26,335 | 26,207 | 11 | 53 | 0,0147 |
| Q83BD6 | tolR | 22,813 | 23,537 | 23,142 | 23,148 | 2 | 9 | 0,3932 |
| Q83BD7 | tolQ | 25,810 | 25,711 | 25,969 | 25,538 | 5 | 35 | 0,1079 |
| Q83BD8 | CBU_1573 | 25,414 | 25,066 | 25,717 | 25,664 | 3 | 30 | 0,0006 |
| Q83BE0 | ruvB | 28,453 | 28,511 | 28,908 | 28,681 | 15 | 197 | 0,0033 |
| Q83BE4 | CBU_1566 | 23,030 | 24,131 | 23,468 | 22,668 | 5 | 22 | 0,4382 |
| Q83BE5 | aspS | 29,449 | 29,583 | 29,449 | 29,543 | 23 | 307 | 0,0458 |
| Q83BE9 | CBU_1561 | 26,134 | 23,285 | 23,718 | 23,780 | 2 | 18 | 0,0008 |
| Q83BF1 | CBU_1559 | 26,550 | 26,279 | 26,397 | 26,006 | 8 | 48 | 0,0006 |
| Q83BF2 | CBU_1558 | 27,429 | 27,390 | 27,983 | 27,953 | 6 | 68 | 0,0010 |
| Q83BF5 | nrdB | 25,499 | 26,346 | 26,199 | 26,735 | 7 | 42 | 0,0000 |
| Q83BF6 | nrdA | 28,394 | 29,186 | 29,171 | 29,348 | 26 | 278 | 0,0000 |
| Q83BF8 | rppH | 23,297 | 25,313 | 24,403 | 24,227 | 3 | 27 | 0,0006 |
| Q83BF9 | ptsP | 26,767 | 27,017 | 26,884 | 26,596 | 13 | 96 | 0,0024 |
| Q83BG1 | CBU_1548 | 26,649 | 25,287 | 26,837 | 26,141 | 2 | 30 | 0,0000 |
| Q83BG2 | thyA | 26,070 | 25,899 | 26,388 | 26,544 | 4 | 41 | 0,0059 |
| Q83BH0 | CBU_1538 | 30,025 | 29,914 | 30,232 | 29,886 | 19 | 219 | 0,0006 |
| Q83BH2 | gpmI | 27,226 | 27,190 | 27,410 | 27,393 | 18 | 124 | 0,1788 |
| Q83BH9 | CBU_1529 | 26,440 | 25,985 | 26,691 | 26,769 | 17 | 107 | 0,0001 |
| Q83BI7 | CBU_1521 | 27,907 | 27,527 | 27,704 | 27,573 | 4 | 42 | 0,0006 |
| Q83BI8 | grxC | 27,376 | 26,869 | 27,330 | 27,234 | 5 | 35 | 0,0082 |
| Q83BI9 | secB | 29,277 | 29,484 | 29,514 | 29,409 | 7 | 164 | 0,4891 |
| Q83BJ0 | gpsA | 27,715 | 27,608 | 27,622 | 27,405 | 8 | 96 | 0,1286 |
| Q83BJ5 | CBU_1513 | 26,045 | 26,394 | 25,228 | 25,333 | 9 | 34 | 0,0035 |
| Q83BJ6 | CBU_1512 | 23,470 | 25,602 | 24,033 | 24,171 | 4 | 8 | 0,0006 |
| Q83BJ8 | accA | 28,427 | 28,580 | 28,531 | 28,447 | 13 | 171 | 0,0206 |
| Q83BJ9 | tilS | 25,061 | 24,964 | 24,865 | 24,695 | 6 | 42 | 0,0102 |
| Q83BK0 | CBU_1508 | 25,319 | 25,172 | 25,102 | 24,861 | 8 | 50 | 0,0033 |
| Q83BK1 | CBU_1507 | 24,599 | 24,628 | 24,984 | 24,259 | 10 | 27 | 0,0494 |
| Q83BK2 | CBU_1506 | 24,300 | 24,173 | 24,469 | 24,450 | 2 | 18 | 0,0050 |
| Q83BK3 | lepA | 27,201 | 27,281 | 27,132 | 27,257 | 14 | 121 | 0,0564 |
| Q83BK4 | lepB-2 | 26,771 | 26,684 | 26,591 | 26,481 | 10 | 74 | 0,4679 |
| Q83BL1 | pdxJ | 26,970 | 26,021 | 26,867 | 26,130 | 5 | 53 | 0,0002 |
| Q83BL6 | gltX2 | 28,348 | 28,653 | 28,828 | 28,814 | 25 | 217 | 0,0009 |
| Q83BL7 | cysS | 27,638 | 27,741 | 27,993 | 28,097 | 22 | 192 | 0,0003 |
| Q83BM1 | CBU_1483 | 25,807 | 24,986 | 26,056 | 25,049 | 6 | 39 | 0,0001 |
| Q83BM6 | CBU_1477 | 23,534 | 24,981 | 23,728 | 23,966 | 6 | 15 | 0,0345 |
| Q83BM7 | oxyR | 24,976 | 25,431 | 25,211 | 25,253 | 9 | 40 | 0,0083 |
| Q83BM8 | gatB | 29,244 | 29,229 | 29,194 | 29,311 | 21 | 239 | 0,1085 |
| Q83BM9 | gatA | 28,641 | 28,752 | 28,561 | 28,717 | 16 | 203 | 0,0051 |
| Q83BN0 | gatC | 26,699 | 26,486 | 26,788 | 26,675 | 4 | 35 | 0,2708 |
| Q83BN2 | mreB | 31,102 | 30,416 | 30,708 | 30,715 | 16 | 273 | 0,0029 |
| Q83BN3 | mreC | 26,602 | 26,172 | 26,754 | 26,293 | 10 | 73 | 0,0021 |
| Q83BN5 | CBU_1468 | 25,162 | 25,632 | 25,321 | 25,332 | 19 | 53 | 0,1417 |
| Q83BN6 | tldD | 24,940 | 25,472 | 25,451 | 25,816 | 9 | 47 | 0,0010 |
| Q83BN7 | CBU_1466 | 24,060 | 22,991 | 23,717 | 23,440 | 3 | 19 | 0,1160 |
| Q83BN9 | hupB | 30,776 | 31,043 | 30,717 | 30,754 | 5 | 210 | 0,1062 |
| Q83BP5 | CBU_1458 | 26,108 | 26,212 | 26,012 | 26,026 | 10 | 46 | 0,1359 |
| Q83BQ2 | CBU_1451 | 28,584 | 28,805 | 28,558 | 28,642 | 20 | 183 | 0,0048 |
| Q83BQ3 | tpiA | 26,329 | 26,240 | 26,357 | 26,368 | 8 | 50 | 0,3001 |
| Q83BQ5 | nuoA | 23,526 | 23,716 | 24,383 | 25,371 | 1 | 12 | 0,2741 |
| Q83BQ6 | nuoB | 23,179 | 23,840 | 23,316 | 23,199 | 5 | 24 | 0,0211 |
| Q83BQ7 | nuoC | 27,961 | 28,002 | 27,840 | 27,973 | 10 | 112 | 0,1549 |
| Q83BQ8 | nuoD | 28,362 | 28,392 | 28,233 | 28,403 | 10 | 169 | 0,0796 |
| Q83BQ9 | nuoE | 26,806 | 27,093 | 26,703 | 26,938 | 6 | 43 | 0,0346 |
| Q83BR0 | nuoF | 27,264 | 27,669 | 27,188 | 27,399 | 11 | 103 | 0,0001 |
| Q83BR1 | nuoG | 29,533 | 29,451 | 29,429 | 29,633 | 27 | 298 | 0,0005 |
| Q83BR2 | nuoH | 25,050 | 25,042 | 25,496 | 24,995 | 3 | 30 | 0,2408 |
| Q83BR3 | nuoI | 27,176 | 27,136 | 27,161 | 27,084 | 8 | 65 | 0,9551 |
| Q83BR6 | nuoL | 26,525 | 26,510 | 26,630 | 27,036 | 4 | 65 | 0,0070 |
| Q83BR7 | nuoM | 26,583 | 26,252 | 26,968 | 27,270 | 4 | 42 | 0,0004 |
| Q83BR9 | rimP | 23,135 | 23,582 | 23,645 | 23,745 | 2 | 14 | 0,0211 |
| Q83BS0 | nusA | 29,888 | 30,030 | 30,073 | 30,114 | 24 | 315 | 0,1431 |
| Q83BS1 | infB | 29,807 | 30,008 | 30,103 | 30,183 | 28 | 362 | 0,0012 |
| Q83BS2 | rbfA | 24,786 | 25,133 | 25,202 | 25,281 | 5 | 30 | 0,2990 |
| Q83BS7 | CBU_1425 | 30,612 | 30,293 | 30,128 | 30,197 | 4 | 96 | 0,0241 |
| Q83BS8 | hemB | 28,016 | 28,100 | 27,893 | 27,888 | 13 | 126 | 0,0014 |
| Q83BT0 | radA | 26,167 | 26,527 | 26,550 | 26,400 | 12 | 116 | 0,0677 |
| Q83BT3 | glyA | 28,282 | 28,228 | 28,454 | 28,359 | 13 | 157 | 0,0496 |
| Q83BT4 | nrdR | 26,568 | 26,477 | 26,789 | 26,744 | 4 | 55 | 0,0024 |
| Q83BT5 | nusB | 25,994 | 26,983 | 26,919 | 26,616 | 6 | 58 | 0,0109 |
| Q83BT6 | CBU_1416 | 24,850 | 22,766 | 24,765 | 23,360 | 6 | 20 | 0,0551 |
| Q83BT7 | thiL | 27,255 | 26,672 | 26,745 | 26,494 | 12 | 77 | 0,0018 |
| Q83BT8 | CBU_1414 | 24,178 | 23,015 | 25,953 | 22,750 | 6 | 24 | 0,0009 |
| Q83BT9 | CBU_1413 | 23,648 | 23,052 | 24,895 | 23,108 | 3 | 6 | 0,0066 |
| Q83BU6 | CBU_1404 | 26,757 | 25,202 | 24,874 | 22,557 | 5 | 20 | 0,0016 |
| Q83BU7 | sucB | 30,739 | 30,672 | 30,712 | 30,720 | 17 | 240 | 0,8823 |
| Q83BU9 | enhA.5 | 25,979 | 22,960 | 23,774 | 22,873 | 2 | 9 | 0,0001 |
| Q83BV1 | map | 27,204 | 27,328 | 27,153 | 27,179 | 10 | 46 | 0,1142 |
| Q83BV3 | pyrH | 27,347 | 27,265 | 27,414 | 27,199 | 11 | 86 | 0,0573 |
| Q83BV4 | frr | 29,147 | 28,523 | 29,048 | 29,057 | 8 | 115 | 0,0029 |
| Q83BV5 | uppS | 25,755 | 25,313 | 24,501 | 24,991 | 4 | 26 | 0,4584 |
| Q83BV6 | cdsA | 22,737 | 23,076 | 23,034 | 22,924 | 1 | 3 | 0,7621 |
| Q83BV7 | CBU_1380 | 28,163 | 27,600 | 27,894 | 27,735 | 6 | 76 | 0,0086 |
| Q83BW2 | relA | 24,064 | 23,515 | 24,226 | 23,836 | 6 | 20 | 0,1303 |
| Q83BW3 | CBU_1374 | 26,488 | 27,064 | 26,889 | 27,014 | 8 | 81 | 0,0006 |
| Q83BW4 | pabB | 26,820 | 26,391 | 26,320 | 26,572 | 17 | 118 | 0,0029 |
| Q83BW5 | CBU_1372 | 24,932 | 25,371 | 24,197 | 24,793 | 3 | 26 | 0,0033 |
| Q83BW6 | CBU_1371 | 24,506 | 24,186 | 23,347 | 23,632 | 3 | 11 | 0,0666 |
| Q83BW7 | CBU_1370 | 23,956 | 23,713 | 23,549 | 24,122 | 7 | 25 | 0,0132 |
| Q83BX1 | CBU_1366 | 25,569 | 25,077 | 25,086 | 25,178 | 4 | 32 | 0,9253 |
| Q83BX4 | CBU_1363 | 23,272 | 23,790 | 23,668 | 23,417 | 6 | 26 | 0,0243 |
| Q83BX5 | czcD.2 | 25,922 | 25,848 | 25,950 | 25,895 | 7 | 15 | 0,8409 |
| Q83BX6 | CBU_1361 | 22,763 | 23,507 | 23,133 | 23,050 | 3 | 12 | 0,1018 |
| Q83BX7 | sufB | 24,846 | 25,209 | 25,156 | 25,187 | 9 | 41 | 0,0229 |
| Q83BX8 | sufC | 25,950 | 26,142 | 26,111 | 25,963 | 8 | 32 | 0,2069 |
| Q83BX9 | sufD | 24,291 | 24,298 | 24,438 | 24,742 | 5 | 14 | 0,0101 |
| Q83BY0 | csdB | 26,525 | 25,999 | 26,139 | 26,342 | 10 | 67 | 0,0001 |
| Q83BY1 | iscU.2 | 23,468 | 24,367 | 24,461 | 24,132 | 3 | 19 | 0,0028 |
| Q83BY2 | CBU_1355 | 23,734 | 22,982 | 23,573 | 23,903 | 3 | 7 | 0,3322 |
| Q83BY4 | rlmE | 23,242 | 23,437 | 23,154 | 22,744 | 2 | 9 | 0,8903 |
| Q83BY5 | ftsH | 30,009 | 29,865 | 29,859 | 29,880 | 24 | 302 | 0,0020 |
| Q83BY6 | folP | 26,622 | 26,158 | 26,751 | 26,183 | 7 | 57 | 0,0145 |
| Q83BY7 | glmM | 26,717 | 27,107 | 27,222 | 27,279 | 12 | 90 | 0,0006 |
| Q83BZ2 | CBU_1345 | 22,879 | 23,067 | 22,970 | 22,895 | 2 | 2 | 0,9605 |
| Q83BZ5 | guaB | 29,293 | 29,614 | 29,530 | 29,259 | 28 | 308 | 0,0059 |
| Q83BZ6 | guaA | 27,938 | 28,603 | 28,242 | 28,031 | 17 | 161 | 0,0001 |
| Q83BZ8 | rnhB | 22,721 | 22,765 | 23,670 | 23,199 | 3 | 14 | 0,0402 |
| Q83BZ9 | ddl | 27,254 | 26,884 | 27,061 | 27,094 | 10 | 92 | 0,1123 |
| Q83C00 | dnaE | 27,536 | 27,189 | 27,696 | 27,884 | 33 | 216 | 0,0005 |
| Q83C03 | CBU_1334 | 24,383 | 23,796 | 23,705 | 23,591 | 4 | 16 | 0,0066 |
| Q83C10 | thrS | 29,619 | 29,438 | 29,501 | 29,659 | 34 | 339 | 0,0148 |
| Q83C11 | infC | 28,881 | 28,872 | 29,020 | 29,193 | 8 | 87 | 0,0231 |
| Q83C12 | rpmI | 23,332 | 23,452 | 24,720 | 24,524 | 2 | 24 | 0,0000 |
| Q83C13 | rplT | 27,479 | 27,827 | 27,962 | 28,090 | 5 | 49 | 0,0006 |
| Q83C14 | pheS | 27,057 | 26,907 | 27,057 | 27,101 | 9 | 65 | 0,0204 |
| Q83C15 | pheT | 28,723 | 28,744 | 28,700 | 28,889 | 26 | 286 | 0,0737 |
| Q83C16 | ihfA | 29,702 | 29,731 | 29,469 | 29,081 | 6 | 129 | 0,0005 |
| Q83C17 | CBU_1319 | 25,488 | 25,395 | 26,094 | 25,926 | 3 | 29 | 0,0008 |
| Q83C26 | CBU_1308 | 27,461 | 23,875 | 25,588 | 26,398 | 11 | 55 | 0,0001 |
| Q83C28 | msrA | 24,617 | 24,667 | 25,303 | 25,122 | 9 | 45 | 0,0019 |
| Q83C29 | smpB | 25,130 | 25,219 | 25,603 | 25,475 | 4 | 52 | 0,0163 |
| Q83C30 | CBU_1304 | 23,850 | 23,875 | 24,013 | 23,249 | 4 | 11 | 0,0912 |
| Q83C31 | CBU_1303 | 22,809 | 23,111 | 23,462 | 23,371 | 2 | 10 | 0,6315 |
| Q83C32 | omlA | 27,916 | 27,242 | 27,593 | 27,659 | 5 | 51 | 0,0006 |
| Q83C33 | fur | 23,436 | 23,349 | 23,410 | 23,329 | 2 | 7 | 0,9867 |
| Q83C37 | recN | 27,793 | 27,831 | 27,892 | 27,790 | 23 | 170 | 0,3953 |
| Q83C38 | nadK | 27,384 | 26,684 | 27,149 | 26,591 | 9 | 80 | 0,0000 |
| Q83C40 | vapC | 23,397 | 22,825 | 22,913 | 24,019 | 2 | 11 | 0,0269 |
| Q83C41 | grpE | 27,702 | 28,008 | 28,008 | 27,363 | 9 | 75 | 0,3840 |
| Q83C42 | CBU_1292 | 27,010 | 26,067 | 26,560 | 26,737 | 10 | 56 | 0,0000 |
| Q83C43 | CBU_1291 | 25,153 | 24,082 | 24,258 | 24,623 | 3 | 28 | 0,0003 |
| Q83C46 | CBU_1286 | 23,373 | 23,961 | 23,476 | 23,366 | 6 | 22 | 0,0117 |
| Q83C48 | CBU_1284 | 24,028 | 23,972 | 24,350 | 24,043 | 8 | 31 | 0,0944 |
| Q83C49 | carA | 26,773 | 27,028 | 26,885 | 26,955 | 9 | 63 | 0,0040 |
| Q83C50 | carB | 28,594 | 29,037 | 28,785 | 28,875 | 33 | 253 | 0,0001 |
| Q83C51 | greA | 27,400 | 27,747 | 27,909 | 27,840 | 6 | 62 | 0,0001 |
| Q83C53 | CBU_1278 | 27,834 | 27,536 | 28,097 | 28,017 | 10 | 116 | 0,0001 |
| Q83C54 | eda | 26,414 | 26,581 | 26,758 | 26,330 | 6 | 63 | 0,1529 |
| Q83C55 | CBU_1276 | 29,119 | 29,202 | 29,146 | 29,324 | 13 | 204 | 0,1434 |
| Q83C56 | rspA | 29,944 | 29,918 | 29,873 | 30,064 | 18 | 284 | 0,1489 |
| Q83C57 | CBU_1274 | 24,775 | 25,261 | 24,730 | 23,898 | 3 | 30 | 0,0431 |
| Q83C58 | pfp | 29,239 | 29,271 | 29,418 | 29,847 | 18 | 276 | 0,0000 |
| Q83C60 | CBU_1269 | 28,177 | 27,714 | 27,981 | 27,810 | 9 | 95 | 0,0010 |
| Q83C61 | CBU_1268 | 28,805 | 28,446 | 28,617 | 28,477 | 18 | 179 | 0,0053 |
| Q83C62 | CBU_1267 | 26,905 | 26,525 | 26,452 | 26,705 | 12 | 70 | 0,0383 |
| Q83C63 | lipA | 26,537 | 26,824 | 26,288 | 26,618 | 10 | 53 | 0,0128 |
| Q83C64 | lipB | 23,778 | 23,854 | 23,425 | 23,910 | 3 | 28 | 0,2331 |
| Q83C67 | CBU_1262 | 23,403 | 22,417 | 22,954 | 23,058 | 2 | 14 | 0,3557 |
| Q83C68 | CBU_1261 | 30,381 | 29,982 | 30,045 | 30,341 | 21 | 291 | 0,0000 |
| Q83C69 | CBU_1260 | 29,541 | 29,911 | 29,218 | 29,622 | 10 | 154 | 0,0018 |
| Q83C70 | nhaP.1 | 26,210 | 24,430 | 25,368 | 24,318 | 6 | 27 | 0,0025 |
| Q83C71 | ndk | 28,865 | 29,992 | 29,664 | 29,906 | 8 | 127 | 0,0000 |
| Q83C74 | CBU_1255 | 26,136 | 26,144 | 26,275 | 25,953 | 6 | 42 | 0,0761 |
| Q83C77 | rlmN | 24,458 | 25,820 | 24,451 | 24,890 | 7 | 36 | 0,0026 |
| Q83C79 | CBU_1249 | 28,571 | 27,948 | 28,570 | 28,002 | 9 | 134 | 0,0020 |
| Q83C80 | hisS | 27,485 | 27,428 | 27,640 | 27,309 | 14 | 117 | 0,0055 |
| Q83C81 | CBU_1247 | 26,544 | 26,644 | 26,718 | 26,703 | 3 | 60 | 0,2443 |
| Q83C83 | der | 27,490 | 27,059 | 27,329 | 27,340 | 21 | 109 | 0,0008 |
| Q83C84 | CBU_1244 | 23,461 | 23,762 | 23,613 | 23,088 | 3 | 14 | 0,4266 |
| Q83C85 | xseA | 24,526 | 25,162 | 24,697 | 24,786 | 9 | 52 | 0,0192 |
| Q83C87 | mdh | 29,369 | 29,456 | 29,604 | 29,619 | 18 | 214 | 0,0328 |
| Q83C88 | tsaD | 24,092 | 24,551 | 24,223 | 22,136 | 6 | 19 | 0,0018 |
| Q83C89 | plsY | 22,239 | 22,856 | 23,129 | 22,762 | 3 | 15 | 0,1399 |
| Q83C91 | CBU_1237 | 23,412 | 23,386 | 23,403 | 23,575 | 2 | 5 | 0,9191 |
| Q83C93 | orn | 26,622 | 26,196 | 26,575 | 26,266 | 8 | 71 | 0,0005 |
| Q83C94 | CBU_1234 | 26,177 | 26,047 | 26,075 | 25,959 | 12 | 62 | 0,6386 |
| Q83C95 | CBU_1233 | 27,939 | 28,120 | 27,338 | 27,196 | 13 | 72 | 0,0004 |
| Q83C98 | CBU_1230 | 25,406 | 25,855 | 25,193 | 25,676 | 15 | 61 | 0,0316 |
| Q83C99 | CBU_1229 | 24,743 | 24,836 | 24,219 | 24,678 | 4 | 35 | 0,0405 |
| Q83CA0 | qseC | 24,700 | 25,996 | 25,908 | 25,888 | 10 | 39 | 0,0003 |
| Q83CA1 | qseB | 28,302 | 29,238 | 28,534 | 28,311 | 13 | 138 | 0,0015 |
| Q83CA2 | CBU_1226 | 30,618 | 30,390 | 30,234 | 30,516 | 68 | 604 | 0,0053 |
| Q83CA3 | CBU_1225 | 25,090 | 23,207 | 24,122 | 23,433 | 2 | 18 | 0,0081 |
| Q83CA4 | CBU_1224 | 27,522 | 26,820 | 26,944 | 26,999 | 14 | 111 | 0,0003 |
| Q83CA5 | kdgK | 27,698 | 27,228 | 27,462 | 27,432 | 10 | 87 | 0,0005 |
| Q83CA6 | dapA | 26,223 | 25,741 | 26,259 | 25,623 | 8 | 64 | 0,0033 |
| Q83CA7 | CBU_1221 | 28,888 | 28,788 | 28,777 | 28,659 | 7 | 103 | 0,1092 |
| Q83CA8 | purC | 28,361 | 27,983 | 28,370 | 28,286 | 9 | 102 | 0,0000 |
| Q83CA9 | CBU_1219 | 22,713 | 22,406 | 23,190 | 23,425 | 3 | 11 | 0,4805 |
| Q83CC3 | CBU_1204 | 27,811 | 26,832 | 27,587 | 26,640 | 18 | 144 | 0,0000 |
| Q83CC4 | CBU_1203 | 25,560 | 25,177 | 25,535 | 24,918 | 9 | 42 | 0,0398 |
| Q83CC6 | queA | 24,833 | 25,660 | 25,289 | 25,495 | 7 | 32 | 0,0003 |
| Q83CC8 | CBU_1198 | 23,307 | 23,315 | 24,380 | 24,091 | 3 | 21 | 0,0018 |
| Q83CD0 | clpA | 26,128 | 23,168 | 26,888 | 22,833 | 16 | 56 | 0,0000 |
| Q83CD1 | infA | 25,682 | 24,543 | 24,659 | 25,746 | 3 | 26 | 0,6666 |
| Q83CD5 | uvrC | 26,652 | 26,270 | 26,145 | 26,192 | 16 | 90 | 0,0024 |
| Q83CD7 | CBU_1183 | 30,114 | 30,766 | 30,938 | 30,717 | 4 | 125 | 0,0082 |
| Q83CD8 | iscS | 22,895 | 24,279 | 23,629 | 24,031 | 5 | 12 | 0,0021 |
| Q83CD9 | thiI | 22,434 | 23,405 | 23,073 | 22,917 | 3 | 12 | 0,0123 |
| Q83CE6 | CBU_1173 | 25,522 | 25,301 | 25,001 | 23,960 | 5 | 26 | 0,0025 |
| Q83CE9 | CBU_1169 | 29,681 | 29,839 | 28,726 | 28,653 | 8 | 102 | 0,0006 |
| Q83CF8 | CBU_1160 | 26,503 | 25,825 | 25,639 | 25,657 | 10 | 41 | 0,1372 |
| Q83CG4 | trpC | 24,555 | 23,857 | 24,390 | 23,825 | 3 | 18 | 0,0018 |
| Q83CG6 | CBU_1151 | 26,409 | 26,385 | 26,942 | 26,988 | 8 | 66 | 0,0001 |
| Q83CG9 | mfd | 27,296 | 27,311 | 27,183 | 27,338 | 30 | 149 | 0,1029 |
| Q83CH2 | yajC | 30,881 | 30,977 | 30,296 | 30,207 | 5 | 258 | 0,0008 |
| Q83CH3 | secD | 29,727 | 29,234 | 29,216 | 29,400 | 20 | 230 | 0,0011 |
| Q83CH4 | secF | 26,898 | 26,553 | 26,357 | 26,568 | 4 | 47 | 0,0005 |
| Q83CH7 | enhA.4 | 26,912 | 23,276 | 24,890 | 22,766 | 6 | 17 | 0,0001 |
| Q83CH8 | enhB.2 | 26,971 | 23,372 | 24,787 | 22,357 | 7 | 26 | 0,0001 |
| Q83CH9 | CBU_1136 | 28,495 | 22,161 | 25,491 | 22,803 | 28 | 78 | 0,0000 |
| Q83CI2 | suhB | 26,945 | 28,069 | 27,781 | 27,764 | 10 | 86 | 0,0000 |
| Q83CI4 | trmJ | 26,681 | 26,532 | 26,597 | 26,576 | 9 | 50 | 0,1181 |
| Q83CI6 | nifS | 25,860 | 26,384 | 26,184 | 26,635 | 11 | 59 | 0,0000 |
| Q83CJ1 | cbpM | 26,811 | 26,487 | 26,753 | 26,829 | 5 | 53 | 0,0007 |
| Q83CJ2 | cbpA | 27,757 | 27,530 | 28,049 | 27,758 | 15 | 89 | 0,0120 |
| Q83CJ3 | enhA.3 | 28,539 | 29,253 | 28,933 | 29,105 | 9 | 147 | 0,0001 |
| Q83CJ5 | CBU_1120 | 26,049 | 25,395 | 25,369 | 25,306 | 8 | 42 | 0,0005 |
| Q83CJ6 | CBU_1119 | 28,230 | 28,189 | 28,042 | 28,104 | 7 | 114 | 0,5865 |
| Q83CJ7 | etfB | 26,712 | 27,431 | 26,985 | 27,149 | 8 | 59 | 0,0001 |
| Q83CJ8 | etfA | 28,146 | 28,664 | 28,511 | 28,650 | 13 | 138 | 0,0006 |
| Q83CJ9 | ald | 28,092 | 28,948 | 28,311 | 28,268 | 15 | 152 | 0,0003 |
| Q83CK4 | CBU_1111 | 28,531 | 28,159 | 27,769 | 27,827 | 14 | 107 | 0,0075 |
| Q83CL1 | CBU_1103 | 26,618 | 25,052 | 25,275 | 26,284 | 7 | 48 | 0,0001 |
| Q83CL4 | CBU_1100 | 24,672 | 25,569 | 25,188 | 24,570 | 3 | 26 | 0,4005 |
| Q83CL5 | lepB-1 | 27,674 | 27,354 | 27,432 | 27,755 | 12 | 102 | 0,0135 |
| Q83CL6 | CBU_1098 | 28,475 | 26,447 | 27,518 | 27,917 | 14 | 108 | 0,0000 |
| Q83CL7 | aldC | 29,152 | 28,576 | 28,754 | 28,028 | 9 | 121 | 0,0001 |
| Q83CL8 | fumC | 28,958 | 28,533 | 28,719 | 28,465 | 14 | 158 | 0,0028 |
| Q83CL9 | CBU_1095 | 25,442 | 24,180 | 24,061 | 23,261 | 4 | 17 | 0,0088 |
| Q83CM0 | CBU_1094 | 28,916 | 28,102 | 28,491 | 27,977 | 15 | 145 | 0,0000 |
| Q83CM1 | CBU_1093 | 27,847 | 26,963 | 27,502 | 27,157 | 18 | 127 | 0,0006 |
| Q83CM3 | vacB | 28,940 | 28,760 | 28,764 | 28,813 | 29 | 266 | 0,3875 |
| Q83CM5 | nnr | 28,737 | 28,865 | 28,639 | 28,608 | 19 | 141 | 0,0017 |
| Q83CM6 | CBU_1087 | 23,146 | 23,147 | 23,506 | 23,183 | 2 | 7 | 0,7065 |
| Q83CM7 | CBU_1085 | 26,795 | 26,938 | 26,902 | 26,234 | 11 | 78 | 0,0038 |
| Q83CM8 | CBU_1084 | 25,058 | 24,771 | 26,083 | 25,213 | 7 | 34 | 0,0027 |
| Q83CM9 | mutL | 26,979 | 27,452 | 27,453 | 27,627 | 16 | 92 | 0,0007 |
| Q83CN1 | CBU_1080 | 24,283 | 24,475 | 24,448 | 24,532 | 4 | 44 | 0,2609 |
| Q83CN2 | CBU_1079 | 25,749 | 25,293 | 25,635 | 25,749 | 4 | 22 | 0,0246 |
| Q83CN4 | nagZ | 25,621 | 26,018 | 25,799 | 25,835 | 11 | 56 | 0,0899 |
| Q83CN5 | CBU_1075 | 23,855 | 24,028 | 23,910 | 23,580 | 3 | 19 | 0,0996 |
| Q83CN6 | CBU_1074 | 27,043 | 26,721 | 27,264 | 27,384 | 2 | 38 | 0,0005 |
| Q83CN7 | CBU_1073 | 25,798 | 25,269 | 25,343 | 25,251 | 6 | 39 | 0,0814 |
| Q83CN8 | tgt | 24,144 | 24,867 | 24,201 | 24,036 | 8 | 15 | 0,0291 |
| Q83CP2 | ttcA | 22,488 | 24,719 | 23,074 | 23,857 | 4 | 26 | 0,0008 |
| Q83CP4 | CBU_1065 | 26,025 | 25,830 | 25,359 | 25,017 | 7 | 55 | 0,0001 |
| Q83CP5 | CBU_1064 | 27,634 | 27,484 | 27,633 | 27,376 | 5 | 86 | 0,0146 |
| Q83CP8 | scpA | 25,073 | 25,020 | 25,458 | 25,240 | 5 | 28 | 0,1355 |
| Q83CP9 | scpB | 28,676 | 28,413 | 28,751 | 28,664 | 14 | 145 | 0,1312 |
| Q83CQ0 | CBU_1058 | 23,500 | 24,233 | 23,924 | 23,924 | 6 | 21 | 0,0008 |
| Q83CQ1 | CBU_1057 | 23,031 | 23,973 | 24,114 | 23,950 | 4 | 24 | 0,0128 |
| Q83CQ2 | mutS | 25,615 | 26,263 | 26,476 | 26,933 | 18 | 88 | 0,0007 |
| Q83CQ3 | CBU_1055 | 25,475 | 24,554 | 25,285 | 24,510 | 3 | 21 | 0,0813 |
| Q83CQ4 | recA | 30,294 | 30,447 | 30,489 | 30,546 | 16 | 279 | 0,0006 |
| Q83CQ5 | recX | 23,025 | 24,119 | 23,667 | 23,807 | 5 | 7 | 0,0277 |
| Q83CQ6 | alaS | 29,714 | 29,629 | 29,716 | 29,645 | 35 | 359 | 0,1253 |
| Q83CQ7 | CBU_1051 | 27,281 | 27,066 | 27,516 | 27,481 | 12 | 83 | 0,0005 |
| Q83CQ8 | csrA2 | 26,230 | 25,769 | 26,640 | 27,211 | 5 | 37 | 0,0009 |
| Q83CR5 | CBU_1042 | 24,755 | 22,624 | 24,145 | 22,971 | 3 | 11 | 0,0011 |
| Q83CR7 | cyoA | 28,860 | 28,853 | 28,628 | 28,916 | 9 | 123 | 0,0502 |
| Q83CR8 | cyoB | 27,171 | 27,067 | 27,196 | 27,379 | 8 | 77 | 0,0204 |
| Q83CS0 | CBU_1035 | 27,784 | 27,207 | 27,647 | 27,324 | 17 | 93 | 0,0024 |
| Q83CS1 | pgsA | 24,471 | 23,387 | 24,665 | 24,243 | 3 | 18 | 0,0006 |
| Q83CS6 | CBU_1027 | 28,235 | 26,440 | 27,124 | 27,043 | 15 | 92 | 0,0000 |
| Q83CT0 | CBU_1023 | 23,079 | 23,545 | 23,745 | 23,037 | 3 | 8 | 0,2186 |
| Q83CT2 | CBU_1021 | 26,604 | 26,352 | 26,537 | 26,279 | 4 | 58 | 0,3035 |
| Q83CT5 | CBU_1018 | 26,188 | 25,561 | 26,346 | 26,648 | 5 | 46 | 0,0004 |
| Q83CT6 | CBU_1017 | 24,037 | 24,072 | 23,936 | 23,515 | 8 | 20 | 0,0178 |
| Q83CU5 | bioB | 23,299 | 22,684 | 23,455 | 24,993 | 2 | 12 | 0,0041 |
| Q83CV0 | birA | 25,007 | 25,643 | 25,611 | 25,117 | 11 | 56 | 0,0006 |
| Q83CV1 | lolC | 26,451 | 26,511 | 26,401 | 26,476 | 10 | 63 | 0,5536 |
| Q83CV2 | lolD | 26,486 | 26,272 | 26,398 | 26,611 | 7 | 79 | 0,3166 |
| Q83CV3 | hflX | 23,406 | 24,851 | 24,476 | 24,484 | 7 | 31 | 0,0001 |
| Q83CV4 | purA | 28,082 | 28,292 | 28,533 | 28,544 | 16 | 112 | 0,0006 |
| Q83CV9 | def1 | 24,785 | 25,509 | 25,728 | 25,865 | 5 | 22 | 0,0000 |
| Q83CW4 | ung | 27,443 | 27,661 | 27,318 | 27,790 | 9 | 125 | 0,0000 |
| Q83CW5 | CBU_0987 | 25,774 | 25,787 | 25,606 | 26,018 | 10 | 36 | 0,4460 |
| Q83CW6 | rlmB | 26,914 | 26,747 | 26,945 | 26,782 | 13 | 112 | 0,0094 |
| Q83CW7 | CBU_0985 | 25,978 | 26,087 | 26,083 | 26,086 | 11 | 38 | 0,9538 |
| Q83CW9 | CBU_0982 | 26,134 | 26,075 | 26,262 | 26,183 | 9 | 64 | 0,0564 |
| Q83CX1 | CBU_0980 | 27,883 | 23,206 | 26,298 | 23,118 | 1 | 26 | 0,0001 |
| Q83CX2 | CBU_0979 | 28,293 | 27,291 | 27,121 | 26,853 | 5 | 79 | 0,0009 |
| Q83CX4 | CBU_0977 | 28,136 | 27,853 | 28,160 | 28,446 | 23 | 155 | 0,0007 |
| Q83CX5 | CBU_0976 | 26,440 | 26,340 | 26,585 | 26,350 | 10 | 88 | 0,6177 |
| Q83CX6 | CBU_0975 | 28,223 | 27,798 | 28,117 | 28,091 | 16 | 136 | 0,0019 |
| Q83CX7 | CBU_0974 | 27,577 | 27,088 | 27,562 | 27,465 | 9 | 112 | 0,0007 |
| Q83CX8 | CBU_0973 | 28,041 | 27,680 | 28,073 | 27,908 | 14 | 84 | 0,0015 |
| Q83CX9 | CBU_0972 | 26,647 | 25,481 | 26,198 | 25,828 | 6 | 51 | 0,0001 |
| Q83CY0 | pyrD | 24,961 | 23,752 | 26,396 | 25,378 | 6 | 21 | 0,0111 |
| Q83CY3 | CBU_0968 | 28,380 | 27,330 | 27,132 | 27,252 | 7 | 83 | 0,0001 |
| Q83CY5 | cydB | 25,168 | 24,454 | 24,651 | 24,058 | 2 | 22 | 0,3765 |
| Q83CY6 | cydA-2 | 28,942 | 28,470 | 28,524 | 28,712 | 12 | 105 | 0,0040 |
| Q83CY7 | CBU_0964 | 22,810 | 25,746 | 24,425 | 24,788 | 5 | 27 | 0,0002 |
| Q83CY8 | bcp | 31,068 | 30,705 | 30,951 | 30,811 | 14 | 367 | 0,0849 |
| Q83CY9 | CBU_0962 | 30,136 | 29,474 | 29,763 | 29,765 | 15 | 156 | 0,0018 |
| Q83CZ0 | CBU_0961 | 23,478 | 23,002 | 23,605 | 23,869 | 2 | 7 | 0,3004 |
| Q83CZ1 | CBU_0960 | 24,756 | 24,272 | 25,363 | 25,303 | 5 | 42 | 0,0082 |
| Q83CZ2 | CBU_0959 | 23,996 | 24,105 | 24,743 | 24,506 | 7 | 46 | 0,0163 |
| Q83CZ5;Q83CR4 | gacA.3;gacA.4 | 25,881 | 26,731 | 26,489 | 26,705 | 9 | 52 | 0,0075 |
| Q83CZ8 | CBU_0952 | 30,444 | 28,918 | 29,552 | 29,397 | 13 | 121 | 0,0004 |
| Q83D01 | rhuM | 24,708 | 24,986 | 24,756 | 24,393 | 10 | 55 | 0,0821 |
| Q83D04 | CBU_0943 | 24,921 | 24,893 | 24,940 | 24,567 | 4 | 12 | 0,9610 |
| Q83D06 | CBU_0941 | 23,296 | 23,883 | 23,763 | 24,866 | 2 | 23 | 0,0135 |
| Q83D08 | CBU_0939 | 28,585 | 28,304 | 28,420 | 28,493 | 21 | 165 | 0,3970 |
| Q83D09 | CBU_0937 | 31,966 | 32,074 | 31,570 | 32,022 | 21 | 505 | 0,0079 |
| Q83D11 | CBU_0935 | 23,894 | 23,849 | 25,993 | 25,057 | 4 | 20 | 0,0001 |
| Q83D12 | CBU_0934 | 26,998 | 27,053 | 27,002 | 26,753 | 10 | 100 | 0,0037 |
| Q83D14 | glpK | 27,318 | 27,070 | 26,740 | 26,990 | 16 | 97 | 0,0008 |
| Q83D15 | glpD | 25,771 | 25,784 | 25,716 | 25,614 | 11 | 59 | 0,4553 |
| Q83D17 | CBU_0929 | 26,571 | 25,414 | 26,645 | 26,540 | 7 | 44 | 0,0001 |
| Q83D18 | pdxH | 28,759 | 28,467 | 28,340 | 28,468 | 12 | 137 | 0,0015 |
| Q83D21 | CBU_0925 | 26,277 | 23,560 | 24,776 | 22,948 | 4 | 22 | 0,0000 |
| Q83D22 | CBU_0924 | 25,680 | 25,003 | 25,610 | 24,991 | 5 | 41 | 0,0080 |
| Q83D25 | CBU_0921 | 24,883 | 23,124 | 23,768 | 23,003 | 2 | 11 | 0,0088 |
| Q83D26 | CBU_0920 | 23,106 | 25,792 | 22,372 | 23,639 | 7 | 19 | 0,0283 |
| Q83D28 | CBU_0916 | 26,101 | 24,643 | 25,933 | 24,138 | 10 | 34 | 0,0000 |
| Q83D29 | enhB.1 | 30,460 | 29,037 | 29,815 | 29,180 | 9 | 141 | 0,0018 |
| Q83D31 | CBU_0913 | 25,190 | 25,194 | 25,003 | 24,661 | 5 | 23 | 0,6569 |
| Q83D32 | prpD | 25,480 | 25,675 | 26,200 | 26,250 | 12 | 66 | 0,0001 |
| Q83D34 | CBU_0910 | 22,269 | 22,791 | 22,310 | 22,059 | 4 | 7 | 0,0186 |
| Q83D37 | yciL | 26,470 | 26,951 | 27,196 | 26,947 | 6 | 64 | 0,0004 |
| Q83D39 | rpmE | 25,321 | 26,461 | 25,845 | 25,814 | 4 | 33 | 0,0030 |
| Q83D46 | CBU_0898 | 26,553 | 26,738 | 26,303 | 26,457 | 4 | 30 | 0,0448 |
| Q83D47 | purF | 28,230 | 28,084 | 28,278 | 28,346 | 13 | 134 | 0,0036 |
| Q83D49 | dedD | 27,055 | 27,853 | 27,558 | 27,446 | 5 | 52 | 0,0023 |
| Q83D50 | folC | 26,793 | 27,304 | 27,510 | 27,662 | 15 | 112 | 0,0001 |
| Q83D51 | accD | 27,411 | 27,134 | 27,381 | 27,355 | 10 | 110 | 0,0196 |
| Q83D52 | CBU_0891 | 28,349 | 28,258 | 28,305 | 28,103 | 7 | 88 | 0,2491 |
| Q83D53 | CBU_0890 | 26,367 | 26,547 | 26,598 | 26,605 | 5 | 35 | 0,0449 |
| Q83D54 | dsbA | 28,309 | 27,868 | 28,188 | 27,669 | 6 | 65 | 0,0001 |
| Q83D56 | coaBC | 24,555 | 25,828 | 25,526 | 25,688 | 8 | 26 | 0,0000 |
| Q83D58 | bipA | 26,743 | 27,203 | 27,087 | 27,248 | 13 | 79 | 0,0001 |
| Q83D66 | asd | 27,484 | 27,507 | 27,775 | 27,667 | 13 | 139 | 0,0143 |
| Q83D67 | aroC | 26,513 | 26,827 | 26,700 | 26,707 | 12 | 76 | 0,1492 |
| Q83D69 | udk | 24,526 | 25,252 | 25,743 | 25,802 | 4 | 18 | 0,0270 |
| Q83D71 | alr | 26,824 | 25,921 | 26,225 | 26,074 | 9 | 55 | 0,0001 |
| Q83D72 | dnaB | 28,453 | 28,390 | 28,499 | 28,585 | 20 | 207 | 0,0166 |
| Q83D73 | rplI | 29,452 | 29,510 | 29,921 | 30,185 | 12 | 155 | 0,0085 |
| Q83D75 | rpsR | 27,767 | 28,197 | 28,214 | 28,416 | 3 | 29 | 0,0001 |
| Q83D76 | rpsF | 25,848 | 26,452 | 26,748 | 25,219 | 6 | 27 | 0,2186 |
| Q83D82 | nadE | 27,743 | 28,019 | 27,638 | 27,661 | 17 | 155 | 0,0031 |
| Q83D83 | lpxK | 22,735 | 23,640 | 23,423 | 23,464 | 4 | 16 | 0,0075 |
| Q83D84 | msbA | 28,138 | 27,958 | 28,090 | 28,002 | 14 | 113 | 0,0770 |
| Q83D86 | mgsA | 23,435 | 23,443 | 23,358 | 23,670 | 4 | 21 | 0,7066 |
| Q83D87 | pnp | 30,157 | 30,579 | 30,251 | 30,408 | 36 | 427 | 0,0082 |
| Q83D88 | rpsO | 26,468 | 27,692 | 27,038 | 27,172 | 3 | 24 | 0,0033 |
| Q83D90 | galU | 27,192 | 27,198 | 27,193 | 27,434 | 6 | 69 | 0,0073 |
| Q83D91 | pgi | 24,426 | 24,308 | 24,314 | 24,472 | 5 | 28 | 0,4701 |
| Q83D92 | ugd | 26,638 | 26,693 | 26,742 | 26,956 | 10 | 132 | 0,0298 |
| Q83D93 | CBU_0845 | 27,515 | 27,447 | 27,492 | 27,506 | 16 | 120 | 0,7020 |
| Q83D94 | CBU_0844 | 26,663 | 26,629 | 26,706 | 26,732 | 11 | 84 | 0,5442 |
| Q83D96 | wecB | 24,060 | 24,543 | 23,514 | 23,766 | 6 | 16 | 0,0015 |
| Q83D97 | CBU_0841 | 23,419 | 23,877 | 23,670 | 23,500 | 3 | 12 | 0,7720 |
| Q83D98 | asnB-2 | 25,149 | 25,637 | 24,617 | 25,104 | 11 | 38 | 0,0036 |
| Q83D99 | CBU_0839 | 23,610 | 23,950 | 23,692 | 24,036 | 4 | 24 | 0,0728 |
| Q83DA0 | CBU_0838 | 24,824 | 25,852 | 24,384 | 25,840 | 2 | 10 | 0,3273 |
| Q83DA2 | CBU_0836 | 25,620 | 26,180 | 24,979 | 25,044 | 7 | 40 | 0,0038 |
| Q83DA3 | CBU_0835 | 24,953 | 25,807 | 24,900 | 25,128 | 3 | 33 | 0,0005 |
| Q83DA4 | CBU_0834 | 23,722 | 24,468 | 22,988 | 23,095 | 4 | 8 | 0,1021 |
| Q83DA5 | CBU_0833 | 26,097 | 26,229 | 25,825 | 25,891 | 11 | 51 | 0,0240 |
| Q83DA6 | CBU_0832 | 25,553 | 26,179 | 25,111 | 25,125 | 4 | 34 | 0,0001 |
| Q83DA7 | asnB-1 | 26,602 | 27,115 | 26,513 | 26,644 | 15 | 86 | 0,0217 |
| Q83DA8 | CBU_0830 | 27,393 | 28,321 | 27,230 | 27,395 | 17 | 108 | 0,0008 |
| Q83DA9 | CBU_0829 | 28,541 | 29,981 | 28,396 | 28,513 | 14 | 196 | 0,0001 |
| Q83DB0 | CBU_0828 | 26,864 | 27,596 | 26,707 | 26,914 | 17 | 122 | 0,0070 |
| Q83DB3 | CBU_0825 | 27,678 | 28,416 | 28,514 | 28,947 | 12 | 143 | 0,0000 |
| Q83DB4 | purB | 28,307 | 28,348 | 28,098 | 28,027 | 19 | 140 | 0,0398 |
| Q83DB5 | sfcA | 26,235 | 26,469 | 25,989 | 26,236 | 14 | 55 | 0,0018 |
| Q83DB6 | CBU_0822 | 24,812 | 25,188 | 24,537 | 24,414 | 6 | 21 | 0,0472 |
| Q83DB9 | CBU_0819 | 23,011 | 25,971 | 24,588 | 22,714 | 7 | 27 | 0,0005 |
| Q83DC0 | CBU_0818 | 25,759 | 25,696 | 25,662 | 24,925 | 5 | 48 | 0,0000 |
| Q83DC1 | aacA4 | 23,094 | 23,485 | 23,171 | 23,584 | 3 | 10 | 0,4924 |
| Q83DC7 | prfC | 25,507 | 25,882 | 25,962 | 25,990 | 12 | 72 | 0,0057 |
| Q83DC8 | CBU_0810 | 23,731 | 23,957 | 23,684 | 23,598 | 3 | 18 | 0,3721 |
| Q83DD0 | valS | 27,716 | 27,719 | 27,797 | 28,001 | 29 | 206 | 0,0006 |
| Q83DD1 | CBU_0807 | 26,629 | 26,594 | 26,504 | 26,495 | 9 | 65 | 0,8549 |
| Q83DD4 | CBU_0804 | 27,066 | 27,504 | 27,288 | 27,298 | 20 | 112 | 0,0042 |
| Q83DD5 | CBU_0803 | 25,265 | 25,698 | 26,330 | 25,841 | 8 | 58 | 0,0005 |
| Q83DD6 | CBU_0802 | 27,782 | 27,109 | 27,739 | 28,228 | 7 | 63 | 0,0000 |
| Q83DE0 | CBU_0798 | 27,712 | 27,460 | 27,170 | 26,899 | 12 | 80 | 0,0037 |
| Q83DE1 | CBU_0797 | 23,593 | 22,926 | 23,562 | 22,837 | 2 | 6 | 0,0145 |
| Q83DE2 | CBU_0796 | 26,355 | 25,695 | 25,136 | 25,614 | 3 | 26 | 0,5096 |
| Q83DE3 | folE | 27,342 | 27,608 | 27,606 | 27,384 | 9 | 74 | 0,0222 |
| Q83DE4 | CBU_0794 | 23,275 | 22,935 | 23,491 | 23,011 | 4 | 7 | 0,7872 |
| Q83DE9 | CBU_0789 | 26,187 | 25,340 | 25,730 | 25,841 | 16 | 67 | 0,0039 |
| Q83DF0 | CBU_0788 | 22,899 | 24,595 | 23,591 | 24,671 | 13 | 22 | 0,0006 |
| Q83DF5 | CBU_0782 | 22,939 | 22,935 | 23,156 | 23,327 | 3 | 5 | 0,9534 |
| Q83DF7 | gacA.2 | 27,770 | 27,533 | 28,185 | 27,646 | 11 | 83 | 0,0017 |
| Q83DG0 | CBU_0776 | 24,528 | 24,944 | 24,858 | 24,828 | 5 | 21 | 0,3271 |
| Q83DG4 | prpC | 26,015 | 25,357 | 26,001 | 25,628 | 7 | 51 | 0,0086 |
| Q83DG5 | prpB | 26,706 | 25,883 | 26,631 | 26,708 | 5 | 51 | 0,0004 |
| Q83DG6 | CBU_0770 | 25,770 | 25,491 | 25,606 | 25,532 | 9 | 51 | 0,0118 |
| Q83DG9 | CBU_0766 | 25,998 | 24,691 | 25,552 | 25,545 | 14 | 61 | 0,0005 |
| Q83DH1 | CBU_0762 | 27,079 | 26,715 | 26,757 | 26,516 | 7 | 59 | 0,0131 |
| Q83DH4 | bamD | 28,630 | 28,363 | 28,429 | 28,462 | 10 | 133 | 0,0626 |
| Q83DH6 | degP.2 | 29,036 | 29,244 | 29,078 | 28,563 | 13 | 166 | 0,0023 |
| Q83DH7 | CBU_0754 | 26,443 | 27,159 | 27,375 | 27,017 | 13 | 92 | 0,0009 |
| Q83DH8 | CBU_0753 | 26,241 | 26,716 | 26,509 | 26,373 | 11 | 74 | 0,0026 |
| Q83DH9 | CBU_0752 | 25,213 | 24,751 | 25,176 | 24,681 | 7 | 40 | 0,0262 |
| Q83DI0 | murA | 24,077 | 23,470 | 24,483 | 23,467 | 5 | 12 | 0,0828 |
| Q83DI1 | CBU_0750 | 27,791 | 27,128 | 27,491 | 27,361 | 11 | 84 | 0,0000 |
| Q83DI2 | CBU_0749 | 24,820 | 24,837 | 24,366 | 24,289 | 5 | 48 | 0,0142 |
| Q83DI3 | lptC | 23,651 | 24,158 | 24,157 | 24,142 | 4 | 26 | 0,0730 |
| Q83DI4 | CBU_0747 | 25,554 | 25,928 | 27,073 | 27,196 | 4 | 9 | 0,4642 |
| Q83DI5 | CBU_0746 | 26,229 | 26,643 | 26,432 | 26,362 | 8 | 67 | 0,0087 |
| Q83DI6 | CBU_0745 | 26,693 | 25,797 | 26,169 | 26,613 | 5 | 40 | 0,0344 |
| Q83DI9 | pmbA | 24,276 | 25,971 | 25,154 | 25,410 | 8 | 47 | 0,0001 |
| Q83DJ0 | lon | 29,584 | 30,083 | 29,817 | 30,009 | 39 | 368 | 0,0022 |
| Q83DJ1 | clpX | 27,321 | 28,181 | 28,009 | 28,527 | 17 | 142 | 0,0004 |
| Q83DJ2 | clpP | 28,283 | 28,313 | 28,257 | 28,222 | 6 | 105 | 0,9251 |
| Q83DJ3 | tig | 29,288 | 29,707 | 30,002 | 30,091 | 20 | 248 | 0,0005 |
| Q83DJ4 | CBU_0736 | 25,972 | 26,680 | 25,905 | 25,954 | 8 | 59 | 0,0000 |
| Q83DJ5 | CBU_0735 | 25,610 | 24,857 | 25,479 | 25,556 | 7 | 47 | 0,0011 |
| Q83DK0 | CBU_0730 | 26,953 | 26,370 | 26,609 | 26,157 | 2 | 29 | 0,0016 |
| Q83DK1 | CBU_0729 | 27,887 | 26,549 | 27,537 | 26,806 | 10 | 78 | 0,0000 |
| Q83DK2 | CBU_0728 | 29,036 | 28,538 | 28,928 | 28,314 | 7 | 50 | 0,0078 |
| Q83DK3 | CBU_0727 | 25,608 | 23,879 | 25,540 | 24,815 | 6 | 24 | 0,0541 |
| Q83DK5 | CBU_0722 | 24,534 | 25,548 | 25,921 | 26,431 | 8 | 33 | 0,0125 |
| Q83DK6 | CBU_0720 | 25,928 | 26,191 | 26,168 | 26,392 | 8 | 53 | 0,0223 |
| Q83DK7 | CBU_0719 | 24,580 | 22,873 | 24,098 | 22,415 | 2 | 10 | 0,0014 |
| Q83DK8 | CBU_0718 | 29,389 | 23,745 | 26,764 | 23,514 | 6 | 30 | 0,0001 |
| Q83DL0 | CBU_0714 | 24,747 | 23,928 | 24,635 | 23,948 | 2 | 22 | 0,0716 |
| Q83DL2 | gacA.1 | 25,556 | 22,962 | 25,777 | 23,589 | 5 | 22 | 0,0006 |
| Q83DM4 | CBU_0675 | 27,636 | 27,671 | 27,600 | 27,841 | 8 | 95 | 0,0134 |
| Q83DM5 | CBU_0674 | 29,925 | 28,823 | 29,419 | 29,582 | 11 | 165 | 0,0002 |
| Q83DM6 | CBU_0673 | 23,895 | 23,964 | 23,271 | 24,235 | 4 | 25 | 0,0613 |
| Q83DM7 | CBU_0672 | 25,909 | 25,802 | 25,722 | 26,180 | 9 | 74 | 0,1005 |
| Q83DM8 | rhlE | 23,458 | 24,852 | 24,509 | 24,624 | 13 | 19 | 0,0128 |
| Q83DN1 | dapD | 28,576 | 28,356 | 28,139 | 28,027 | 14 | 114 | 0,0012 |
| Q83DN2 | dapE | 27,946 | 27,248 | 27,174 | 27,169 | 12 | 107 | 0,0004 |
| Q83DN8 | dnaZX | 27,456 | 26,836 | 27,353 | 27,657 | 19 | 115 | 0,0003 |
| Q83DN9 | CBU_0658 | 28,808 | 28,501 | 28,963 | 29,205 | 7 | 76 | 0,0081 |
| Q83DP0 | recR | 23,665 | 23,681 | 23,409 | 23,643 | 5 | 22 | 0,1359 |
| Q83DP1 | CBU_0656 | 27,379 | 25,999 | 27,193 | 27,300 | 1 | 30 | 0,0943 |
| Q83DP7 | CBU_0649 | 26,243 | 26,755 | 26,266 | 26,837 | 3 | 40 | 0,0139 |
| Q83DP8 | ribH | 28,899 | 29,812 | 29,195 | 29,205 | 6 | 102 | 0,0372 |
| Q83DP9 | ribA | 26,935 | 27,084 | 26,856 | 27,496 | 16 | 80 | 0,1922 |
| Q83DQ0 | ribE | 23,868 | 26,322 | 26,643 | 26,257 | 6 | 32 | 0,0052 |
| Q83DQ3 | ribD | 25,877 | 25,460 | 25,619 | 25,526 | 8 | 46 | 0,0407 |
| Q83DQ4 | CBU_0642 | 25,371 | 25,007 | 25,065 | 24,960 | 4 | 40 | 0,0050 |
| Q83DQ5 | CBU_0641 | 28,813 | 28,854 | 28,930 | 28,967 | 17 | 219 | 0,0070 |
| Q83DQ6 | CBU_0640 | 28,385 | 28,555 | 27,925 | 28,268 | 16 | 150 | 0,0007 |
| Q83DQ7 | CBU_0639 | 28,062 | 28,040 | 27,912 | 27,986 | 8 | 54 | 0,0579 |
| Q83DQ8 | CBU_0638 | 29,054 | 28,572 | 28,904 | 29,080 | 19 | 146 | 0,0013 |
| Q83DQ9 | CBU_0637 | 26,107 | 25,456 | 26,507 | 26,957 | 5 | 29 | 0,0001 |
| Q83DR2 | CBU_0634 | 27,031 | 26,347 | 27,095 | 26,754 | 16 | 99 | 0,0006 |
| Q83DR4 | CBU_0632 | 29,000 | 29,804 | 29,307 | 29,277 | 12 | 143 | 0,0895 |
| Q83DR5 | purL | 27,636 | 27,816 | 27,576 | 27,820 | 36 | 258 | 0,0223 |
| Q83DR6 | putA | 31,015 | 30,859 | 30,596 | 30,789 | 51 | 579 | 0,1456 |
| Q83DR7 | ppa | 29,963 | 29,676 | 29,414 | 29,425 | 12 | 157 | 0,0051 |
| Q83DS3 | tadA | 24,168 | 24,777 | 25,217 | 24,678 | 4 | 25 | 0,0163 |
| Q83DS4 | CBU_0621 | 28,887 | 28,589 | 29,133 | 29,090 | 12 | 195 | 0,0063 |
| Q83DS5 | lpxB | 23,450 | 23,507 | 23,967 | 24,364 | 4 | 16 | 0,0010 |
| Q83DS9 | CBU_0616 | 25,878 | 26,147 | 25,968 | 25,842 | 7 | 55 | 0,1364 |
| Q83DT0 | lpxD | 27,372 | 28,033 | 27,428 | 27,427 | 11 | 74 | 0,0005 |
| Q83DT1 | ompH | 32,491 | 31,554 | 31,742 | 31,149 | 9 | 217 | 0,0070 |
| Q83DT2 | yaeT | 31,054 | 31,192 | 30,928 | 31,185 | 36 | 551 | 0,0505 |
| Q83DT3 | CBU_0609 | 23,476 | 23,138 | 24,791 | 25,199 | 4 | 25 | 0,0080 |
| Q83DT4 | CBU_0608 | 24,353 | 25,007 | 24,212 | 24,511 | 9 | 29 | 0,0188 |
| Q83DT5 | mvaD | 25,917 | 25,597 | 25,581 | 25,694 | 12 | 65 | 0,0087 |
| Q83DU3 | cysQ-1 | 25,473 | 25,288 | 25,090 | 25,263 | 7 | 61 | 0,0069 |
| Q83DU4 | CBU_0598 | 25,247 | 25,215 | 25,539 | 24,462 | 4 | 23 | 0,1011 |
| Q83DU5 | CBU_0597 | 23,018 | 23,566 | 22,972 | 23,268 | 3 | 11 | 0,0165 |
| Q83DV1 | CBU_0591 | 25,903 | 26,482 | 26,400 | 26,791 | 9 | 72 | 0,0020 |
| Q83DV3 | CBU_0589 | 26,531 | 26,665 | 26,222 | 26,293 | 6 | 37 | 0,1987 |
| Q83DV4 | nadA | 27,338 | 27,065 | 27,241 | 27,449 | 11 | 127 | 0,0005 |
| Q83DV5 | glpE | 28,198 | 28,207 | 27,967 | 27,943 | 5 | 52 | 0,5006 |
| Q83DV6 | CBU_0586 | 27,823 | 27,409 | 27,706 | 27,438 | 27 | 177 | 0,0069 |
| Q83DV7 | CBU_0585 | 25,971 | 25,725 | 25,673 | 25,883 | 2 | 22 | 0,0026 |
| Q83DV9 | CBU_0583 | 27,734 | 29,163 | 28,246 | 28,258 | 5 | 78 | 0,0000 |
| Q83DW0 | bolA | 25,387 | 26,439 | 25,967 | 26,034 | 4 | 35 | 0,0019 |
| Q83DW1 | CBU_0581 | 24,838 | 24,401 | 24,866 | 24,685 | 3 | 17 | 0,0007 |
| Q83DW2 | CBU_0580 | 25,349 | 24,023 | 25,418 | 24,827 | 8 | 19 | 0,0489 |
| Q83DW4 | CBU_0578 | 25,234 | 25,506 | 24,924 | 24,926 | 4 | 17 | 0,0036 |
| Q83DW6 | yfcX | 28,378 | 27,825 | 28,212 | 27,921 | 22 | 194 | 0,0004 |
| Q83DW8 | CBU_0574 | 27,587 | 26,943 | 27,470 | 27,122 | 11 | 96 | 0,0002 |
| Q83DW9 | CBU_0573 | 26,342 | 26,384 | 26,369 | 26,163 | 19 | 101 | 0,0806 |
| Q83DX0 | CBU_0572 | 29,062 | 28,876 | 28,897 | 28,558 | 21 | 256 | 0,0018 |
| Q83DX2 | CBU_0570 | 26,688 | 26,972 | 27,070 | 27,165 | 5 | 35 | 0,0040 |
| Q83DX3 | miaB | 26,651 | 26,173 | 25,460 | 26,315 | 10 | 58 | 0,0003 |
| Q83DX4 | CBU_0568 | 28,295 | 27,226 | 28,309 | 27,625 | 15 | 117 | 0,0000 |
| Q83DX5 | ybeY | 23,906 | 23,323 | 23,697 | 23,620 | 3 | 12 | 0,7040 |
| Q83DX7 | corC | 26,611 | 26,969 | 27,028 | 26,863 | 10 | 82 | 0,0103 |
| Q83DX9 | CBU_0562 | 25,430 | 24,867 | 25,257 | 25,039 | 9 | 49 | 0,0006 |
| Q83DY0 | CBU_0560 | 29,586 | 27,727 | 28,391 | 28,781 | 13 | 135 | 0,0000 |
| Q83DY1 | leuS | 29,140 | 29,201 | 29,044 | 29,181 | 25 | 255 | 0,0955 |
| Q83DY2 | lptE | 26,423 | 26,266 | 26,199 | 26,286 | 7 | 61 | 0,2276 |
| Q83DY3 | holA | 25,767 | 26,055 | 25,719 | 26,137 | 7 | 33 | 0,0732 |
| Q83DY4 | nadD | 25,222 | 24,993 | 24,612 | 24,584 | 6 | 16 | 0,0180 |
| Q83DY7 | rsfS | 25,926 | 26,186 | 26,516 | 26,616 | 3 | 42 | 0,0020 |
| Q83DY8 | pbpA | 25,882 | 25,851 | 25,967 | 25,013 | 10 | 55 | 0,0002 |
| Q83DY9 | rodA | 24,651 | 23,876 | 24,303 | 24,294 | 3 | 17 | 0,0347 |
| Q83DZ0 | CBU_0548 | 27,720 | 26,949 | 27,390 | 26,835 | 14 | 114 | 0,0004 |
| Q83DZ1 | CBU_0547 | 25,391 | 25,937 | 25,310 | 25,559 | 10 | 60 | 0,0013 |
| Q83DZ2 | htpX | 27,574 | 25,840 | 26,738 | 26,284 | 5 | 30 | 0,0003 |
| Q83DZ3 | lemA | 29,718 | 28,602 | 28,802 | 28,197 | 8 | 134 | 0,0003 |
| Q83DZ6 | ligA | 27,866 | 27,606 | 27,686 | 27,719 | 23 | 139 | 0,0238 |
| Q83DZ7 | zipA | 25,607 | 25,220 | 25,049 | 24,874 | 6 | 24 | 0,0121 |
| Q83DZ8 | CBU_0539 | 23,886 | 23,750 | 24,400 | 26,366 | 3 | 10 | 0,0001 |
| Q83E02 | CBU_0535 | 29,815 | 28,279 | 29,025 | 28,734 | 12 | 145 | 0,0001 |
| Q83E04 | rfe | 22,894 | 24,126 | 23,903 | 22,471 | 2 | 7 | 0,0163 |
| Q83E05 | CBU_0532 | 27,884 | 27,467 | 27,992 | 27,684 | 3 | 39 | 0,2595 |
| Q83E06 | pyrF | 27,472 | 27,212 | 27,242 | 27,135 | 10 | 88 | 0,0008 |
| Q83E07 | lapB | 25,781 | 25,739 | 25,690 | 25,605 | 9 | 47 | 0,4991 |
| Q83E08 | CBU_0529 | 24,144 | 24,617 | 23,963 | 23,116 | 2 | 17 | 0,0018 |
| Q83E09 | rpsA | 31,142 | 31,505 | 31,631 | 31,791 | 30 | 436 | 0,0004 |
| Q83E10 | cmk | 27,073 | 26,376 | 27,021 | 26,895 | 9 | 77 | 0,0033 |
| Q83E11 | aroA | 26,752 | 26,515 | 26,658 | 26,668 | 11 | 83 | 0,8378 |
| Q83E12 | serC | 29,269 | 28,734 | 29,108 | 29,082 | 13 | 204 | 0,0002 |
| Q83E13 | gyrA | 29,032 | 29,229 | 29,249 | 29,209 | 34 | 300 | 0,0373 |
| Q83E15 | mtaD | 29,547 | 29,295 | 29,421 | 29,572 | 17 | 182 | 0,0015 |
| Q83E16 | leuA | 26,058 | 26,071 | 25,903 | 25,622 | 10 | 62 | 0,0057 |
| Q83E17 | CBU_0519 | 22,946 | 23,226 | 23,724 | 23,533 | 4 | 12 | 0,3529 |
| Q83E18 | uvrB | 27,638 | 27,425 | 27,574 | 27,445 | 21 | 124 | 0,0138 |
| Q83E19 | aspB | 30,147 | 30,535 | 30,583 | 30,637 | 20 | 265 | 0,0082 |
| Q83E24 | CBU_0510 | 30,334 | 30,645 | 30,369 | 30,300 | 8 | 196 | 0,3918 |
| Q83E25 | trpR | 24,721 | 23,764 | 23,868 | 24,281 | 2 | 21 | 0,0975 |
| Q83E28 | recJ | 27,174 | 26,918 | 28,281 | 27,620 | 17 | 144 | 0,0006 |
| Q83E29 | CBU_0505 | 24,063 | 24,099 | 24,223 | 24,351 | 5 | 37 | 0,4103 |
| Q83E31 | glnA | 30,466 | 30,537 | 30,515 | 30,608 | 14 | 254 | 0,3161 |
| Q83E32 | CBU_0502 | 27,526 | 27,284 | 27,916 | 27,825 | 9 | 89 | 0,0008 |
| Q83E34 | holB | 23,830 | 23,556 | 23,304 | 24,237 | 5 | 30 | 0,0403 |
| Q83E35 | tmk | 26,815 | 26,473 | 26,991 | 26,698 | 10 | 52 | 0,0111 |
| Q83E37 | fabF | 29,195 | 29,177 | 29,219 | 29,160 | 14 | 192 | 0,9249 |
| Q83E38 | acpP | 31,274 | 31,172 | 31,273 | 31,150 | 3 | 95 | 0,9365 |
| Q83E39 | fabD | 28,479 | 28,437 | 28,672 | 28,538 | 9 | 111 | 0,5238 |
| Q83E40 | plsX | 29,861 | 30,159 | 29,758 | 29,896 | 24 | 265 | 0,0017 |
| Q83E41 | rpmF | 26,204 | 26,370 | 26,961 | 26,878 | 2 | 40 | 0,0017 |
| Q83E42 | CBU_0490 | 23,371 | 23,613 | 22,813 | 23,469 | 6 | 20 | 0,0741 |
| Q83E43 | CBU_0489 | 26,448 | 26,001 | 26,273 | 25,126 | 6 | 41 | 0,0629 |
| Q83E44 | CBU_0488 | 25,120 | 23,793 | 24,482 | 23,923 | 7 | 27 | 0,0061 |
| Q83E45 | rne | 29,232 | 29,640 | 29,282 | 29,553 | 28 | 427 | 0,0003 |
| Q83E49 | CBU_0482 | 26,689 | 25,699 | 26,149 | 25,284 | 6 | 36 | 0,0189 |
| Q83E50 | artP | 26,915 | 27,440 | 26,836 | 26,943 | 11 | 85 | 0,0006 |
| Q83E51 | argR | 23,559 | 24,474 | 23,946 | 22,812 | 4 | 10 | 0,1757 |
| Q83E52 | kdsB | 28,830 | 28,490 | 28,834 | 28,810 | 15 | 157 | 0,0018 |
| Q83E55 | CBU_0476 | 25,356 | 24,762 | 25,765 | 25,158 | 10 | 53 | 0,0001 |
| Q83E58 | CBU_0473 | 31,038 | 31,864 | 31,053 | 31,129 | 8 | 304 | 0,0272 |
| Q83E59 | recQ | 23,581 | 24,117 | 22,474 | 23,679 | 6 | 10 | 0,0067 |
| Q83E61 | CBU_0470 | 24,685 | 23,001 | 23,866 | 22,893 | 2 | 16 | 0,0024 |
| Q83E62 | CBU_0469 | 25,240 | 23,250 | 24,780 | 22,714 | 4 | 6 | 0,0003 |
| Q83E63 | xseB | 26,619 | 27,297 | 27,216 | 26,901 | 6 | 78 | 0,0345 |
| Q83E64 | bioC1 | 24,302 | 24,887 | 24,663 | 25,069 | 4 | 19 | 0,0279 |
| Q83E67 | lpdA | 30,786 | 30,871 | 30,856 | 30,803 | 25 | 416 | 0,2410 |
| Q83E68 | pdhC | 29,026 | 28,889 | 28,673 | 28,730 | 20 | 201 | 0,0012 |
| Q83E69 | pdhA | 30,354 | 30,579 | 30,126 | 30,277 | 38 | 469 | 0,0008 |
| Q83E70 | ampE | 27,364 | 26,717 | 27,180 | 27,205 | 4 | 41 | 0,0050 |
| Q83E74 | CBU_0455 | 25,705 | 24,854 | 26,095 | 25,433 | 6 | 28 | 0,0001 |
| Q83E75 | adk | 27,320 | 27,072 | 27,529 | 27,442 | 8 | 79 | 0,0011 |
| Q83E79 | ffh | 26,637 | 27,362 | 27,004 | 27,345 | 9 | 91 | 0,0023 |
| Q83E81 | CBU_0447 | 23,329 | 23,619 | 23,749 | 23,527 | 5 | 12 | 0,3499 |
| Q83E82 | CBU_0446 | 23,519 | 24,449 | 24,032 | 24,155 | 3 | 34 | 0,0320 |
| Q83E83 | rpsP | 27,273 | 27,961 | 27,885 | 28,166 | 7 | 60 | 0,0008 |
| Q83E85 | rplS | 28,483 | 28,994 | 28,888 | 29,060 | 6 | 103 | 0,0078 |
| Q83E86 | ogt | 23,498 | 23,618 | 23,532 | 24,515 | 5 | 21 | 0,1410 |
| Q83E94 | CBU_0433 | 25,867 | 25,481 | 25,569 | 23,558 | 6 | 28 | 0,0001 |
| Q83E96 | CBU_0431 | 26,717 | 26,123 | 26,426 | 26,327 | 7 | 66 | 0,0011 |
| Q83E97 | lysS | 29,214 | 29,451 | 29,373 | 29,422 | 23 | 284 | 0,0014 |
| Q83EA2 | panB | 24,754 | 25,512 | 25,360 | 25,464 | 6 | 26 | 0,0000 |
| Q83EA3 | panC | 26,128 | 26,903 | 27,046 | 27,275 | 11 | 93 | 0,0000 |
| Q83EA7 | CBU_0419 | 25,283 | 23,373 | 24,539 | 22,484 | 4 | 15 | 0,0003 |
| Q83EA8 | CBU_0418 | 24,609 | 24,427 | 24,351 | 24,437 | 6 | 32 | 0,2210 |
| Q83EC9 | ileS | 28,785 | 29,125 | 29,102 | 29,167 | 28 | 246 | 0,0001 |
| Q83ED0 | CBU_0395 | 29,433 | 28,231 | 29,235 | 28,796 | 11 | 142 | 0,0000 |
| Q83ED4 | ribF | 26,255 | 26,165 | 26,172 | 26,296 | 10 | 52 | 0,1958 |
| Q83ED5 | mviN | 24,079 | 24,153 | 24,333 | 24,445 | 2 | 20 | 0,2828 |
| Q83ED6 | rpsT | 25,651 | 27,364 | 27,305 | 27,351 | 1 | 22 | 0,0323 |
| Q83ED8 | obg | 26,428 | 26,249 | 26,431 | 26,393 | 12 | 78 | 0,3390 |
| Q83ED9 | rpmA | 27,660 | 28,334 | 28,099 | 28,360 | 3 | 47 | 0,0047 |
| Q83EE0 | rplU | 27,725 | 28,028 | 28,395 | 28,441 | 3 | 32 | 0,0021 |
| Q83EE1 | tag | 27,173 | 27,600 | 27,368 | 27,282 | 3 | 18 | 0,0103 |
| Q83EE2 | ispB | 24,752 | 25,604 | 25,470 | 25,679 | 8 | 57 | 0,0008 |
| Q83EE5 | ampD | 28,167 | 27,384 | 27,545 | 27,391 | 17 | 108 | 0,0001 |
| Q83EE6 | CBU_0378 | 24,670 | 24,734 | 24,291 | 23,277 | 3 | 21 | 0,0083 |
| Q83EF3 | CBU_0370 | 28,042 | 24,061 | 28,326 | 26,718 | 2 | 44 | 0,0000 |
| Q83EF5 | CBU_0368 | 27,886 | 27,759 | 27,898 | 27,713 | 11 | 128 | 0,2788 |
| Q83EF6 | phoB | 24,825 | 24,743 | 24,921 | 24,501 | 5 | 42 | 0,3689 |
| Q83EF7 | phoR | 24,684 | 24,716 | 24,445 | 24,424 | 8 | 38 | 0,1865 |
| Q83EF9 | CBU_0364 | 23,461 | 24,187 | 23,730 | 24,095 | 2 | 9 | 0,6800 |
| Q83EG9 | CBU_0353 | 28,054 | 27,665 | 28,033 | 27,726 | 13 | 132 | 0,0001 |
| Q83EH1 | CBU_0351 | 26,320 | 26,306 | 26,665 | 26,751 | 8 | 63 | 0,1787 |
| Q83EH2 | gph | 24,949 | 24,752 | 24,361 | 24,415 | 5 | 28 | 0,0117 |
| Q83EH4 | CBU_0347 | 26,341 | 26,010 | 25,726 | 24,999 | 3 | 25 | 0,2157 |
| Q83EH5 | xylB | 24,454 | 24,708 | 23,522 | 23,319 | 5 | 12 | 0,0145 |
| Q83EH9 | pfkA | 25,273 | 25,000 | 25,523 | 25,386 | 3 | 30 | 0,0015 |
| Q83EI2 | pepN | 30,082 | 30,203 | 30,156 | 30,430 | 42 | 440 | 0,0001 |
| Q83EI3 | fis | 28,121 | 28,811 | 28,418 | 28,997 | 4 | 53 | 0,0162 |
| Q83EI4 | purH | 26,883 | 26,806 | 27,129 | 27,007 | 17 | 124 | 0,0042 |
| Q83EJ0 | thiC | 23,504 | 23,511 | 23,681 | 23,104 | 4 | 7 | 0,4719 |
| Q83EJ4 | purD | 30,443 | 30,146 | 29,727 | 29,746 | 16 | 239 | 0,0023 |
| Q83EJ6 | CBU_0324 | 24,460 | 24,228 | 23,564 | 23,943 | 10 | 24 | 0,0733 |
| Q83EJ8 | CBU_0322 | 22,960 | 22,801 | 22,189 | 22,284 | 6 | 24 | 0,0096 |
| Q83EK2 | dnaQ | 25,545 | 25,714 | 25,741 | 25,781 | 6 | 52 | 0,3797 |
| Q83EK3 | rnhA | 25,036 | 25,507 | 25,143 | 25,319 | 4 | 32 | 0,0015 |
| Q83EK5 | gloB | 23,628 | 22,516 | 23,097 | 23,860 | 4 | 9 | 0,1308 |
| Q83EK6 | CBU_0313 | 25,993 | 25,775 | 24,429 | 25,062 | 10 | 35 | 0,0290 |
| Q83EK7 | folD | 25,927 | 26,128 | 26,236 | 26,117 | 6 | 53 | 0,0169 |
| Q83EK8 | ompP1 | 25,011 | 23,437 | 24,645 | 23,639 | 5 | 19 | 0,0519 |
| Q83EL0 | htpG | 31,654 | 32,136 | 31,628 | 32,045 | 40 | 698 | 0,0005 |
| Q83EL2 | CBU_0307 | 27,723 | 27,636 | 27,232 | 27,600 | 9 | 58 | 0,5852 |
| Q83EL4 | recG | 22,696 | 23,301 | 22,931 | 23,598 | 7 | 19 | 0,0312 |
| Q83EL5 | CBU_0304 | 27,506 | 27,647 | 27,579 | 27,145 | 3 | 64 | 0,3483 |
| Q83EL6 | rpoZ | 27,995 | 27,925 | 28,143 | 28,470 | 7 | 88 | 0,0008 |
| Q83EL7 | gmk | 25,807 | 25,684 | 25,566 | 25,058 | 6 | 28 | 0,1662 |
| Q83EL8 | yicC | 25,069 | 25,384 | 25,091 | 24,998 | 7 | 38 | 0,0079 |
| Q83EL9 | rph | 27,204 | 27,716 | 27,019 | 27,107 | 9 | 66 | 0,0000 |
| Q83EM0 | murI | 25,885 | 25,604 | 25,805 | 25,606 | 5 | 54 | 0,0291 |
| Q83EM1 | xth | 27,981 | 28,312 | 27,796 | 27,822 | 12 | 118 | 0,0055 |
| Q83EM3 | CBU_0294 | 26,855 | 26,145 | 26,840 | 26,659 | 14 | 94 | 0,0004 |
| Q83EM4 | rpmB | 26,989 | 27,546 | 27,363 | 27,636 | 6 | 32 | 0,0111 |
| Q83EM5 | rpmG | 25,201 | 26,079 | 24,991 | 25,428 | 4 | 30 | 0,0367 |
| Q83EM7 | coaD | 28,096 | 28,123 | 28,286 | 27,909 | 7 | 111 | 0,1940 |
| Q83EM9 | pcnB | 27,358 | 27,267 | 27,272 | 27,258 | 16 | 105 | 0,1413 |
| Q83EN0 | CBU_0285 | 23,754 | 22,917 | 23,589 | 23,058 | 2 | 10 | 0,1677 |
| Q83EN6 | CBU_0279 | 25,085 | 25,054 | 24,871 | 24,447 | 3 | 24 | 0,0936 |
| Q83EN9 | CBU_0276 | 24,552 | 24,410 | 23,403 | 23,992 | 6 | 31 | 0,0322 |
| Q83EP0 | hemE | 25,230 | 25,378 | 24,830 | 24,133 | 5 | 55 | 0,0452 |
| Q83EP1 | uvrA | 28,065 | 29,138 | 28,583 | 28,228 | 27 | 156 | 0,0001 |
| Q83EP3 | CBU_0272 | 28,180 | 27,327 | 28,063 | 28,073 | 4 | 45 | 0,0001 |
| Q83EP4 | ssb | 29,737 | 29,688 | 29,760 | 29,882 | 7 | 110 | 0,9757 |
| Q83EP5 | CBU_0270 | 29,530 | 29,466 | 29,572 | 29,541 | 22 | 285 | 0,2186 |
| Q83EQ0 | CBU_0265 | 24,815 | 24,661 | 24,596 | 24,948 | 4 | 25 | 0,1484 |
| Q83EQ1 | rplQ | 27,728 | 28,231 | 28,284 | 28,532 | 4 | 60 | 0,0085 |
| Q83EQ2 | rpoA | 31,486 | 31,942 | 31,736 | 31,871 | 24 | 564 | 0,0003 |
| Q83EQ3 | rpsD | 29,618 | 29,999 | 30,283 | 30,382 | 18 | 167 | 0,0000 |
| Q83EQ4 | rpsK | 27,905 | 28,167 | 28,323 | 28,539 | 5 | 57 | 0,0005 |
| Q83EQ6 | secY | 27,140 | 26,935 | 27,144 | 27,490 | 9 | 58 | 0,0018 |
| Q83EQ7 | rplO | 27,983 | 28,331 | 28,506 | 28,653 | 7 | 56 | 0,0005 |
| Q83EQ8 | rpmD | 25,430 | 24,299 | 24,703 | 23,959 | 2 | 18 | 0,8684 |
| Q83EQ9 | rpsE | 27,379 | 27,823 | 27,959 | 28,078 | 8 | 79 | 0,0010 |
| Q83ER0 | rplR | 27,726 | 28,293 | 28,320 | 28,477 | 4 | 60 | 0,0020 |
| Q83ER1 | rplF | 27,775 | 28,179 | 28,392 | 28,501 | 7 | 97 | 0,0010 |
| Q83ER2 | rpsH | 28,999 | 29,231 | 29,705 | 29,816 | 8 | 109 | 0,0001 |
| Q83ER3 | rpsN | 26,493 | 26,732 | 26,812 | 25,856 | 5 | 48 | 0,4718 |
| Q83ER4 | rplE | 28,678 | 29,287 | 29,010 | 29,312 | 14 | 132 | 0,0002 |
| Q83ER5 | rplX | 28,446 | 28,763 | 29,087 | 29,215 | 8 | 82 | 0,0001 |
| Q83ER6 | rplN | 28,443 | 28,938 | 29,050 | 29,154 | 9 | 106 | 0,0006 |
| Q83ER7 | rpsQ | 26,472 | 25,540 | 26,565 | 28,041 | 4 | 29 | 0,4007 |
| Q83ER8;REV__Q83ER8 | rpmC | 26,202 | 26,824 | 26,771 | 26,886 | 4 | 45 | 0,0083 |
| Q83ER9 | rplP | 27,864 | 28,334 | 28,381 | 28,555 | 5 | 79 | 0,0068 |
| Q83ES1 | rplB | 28,728 | 29,183 | 29,203 | 29,273 | 10 | 143 | 0,0007 |
| Q83ES2 | rplW | 26,713 | 27,502 | 26,911 | 27,255 | 3 | 43 | 0,0300 |
| Q83ES3 | rplD | 28,556 | 29,283 | 29,080 | 29,278 | 14 | 162 | 0,0032 |
| Q83ES4 | rplC | 28,926 | 29,363 | 29,659 | 29,678 | 12 | 191 | 0,0004 |
| Q83ES5 | rpsJ | 28,200 | 28,619 | 28,726 | 28,786 | 7 | 81 | 0,0075 |
| Q83ES6 | tufA | 33,176 | 33,048 | 33,464 | 33,369 | 30 | 916 | 0,0037 |
| Q83ES7 | fusA | 31,218 | 31,549 | 31,609 | 31,655 | 31 | 533 | 0,0001 |
| Q83ES8 | rpsG | 29,812 | 30,388 | 30,368 | 30,442 | 8 | 146 | 0,0017 |
| Q83ES9 | rpsL | 27,101 | 27,285 | 27,716 | 27,758 | 4 | 49 | 0,0032 |
| Q83ET0 | rpoC | 32,451 | 32,586 | 32,638 | 32,850 | 77 | 1160 | 0,0018 |
| Q83ET2 | rplJ | 28,430 | 29,057 | 28,953 | 29,091 | 6 | 132 | 0,0212 |
| Q83ET3 | rplA | 29,648 | 30,162 | 30,320 | 30,454 | 11 | 177 | 0,0006 |
| Q83ET4 | rplK | 28,997 | 29,350 | 29,629 | 29,747 | 8 | 138 | 0,0000 |
| Q83ET5 | nusG | 29,222 | 29,237 | 29,482 | 29,519 | 9 | 145 | 0,0190 |
| Q83ET9 | CBU_0221 | 24,860 | 24,756 | 24,484 | 24,549 | 5 | 31 | 0,0145 |
| Q83EU4 | CBU_0215 | 28,914 | 28,099 | 28,061 | 27,729 | 16 | 144 | 0,0001 |
| Q83EU5 | CBU_0214 | 24,385 | 24,660 | 23,540 | 24,731 | 3 | 17 | 0,0679 |
| Q83EV3 | gltX1 | 27,300 | 27,973 | 27,604 | 27,696 | 21 | 158 | 0,0003 |
| Q83EV7 | CBU_0201 | 27,099 | 27,047 | 27,537 | 27,230 | 5 | 51 | 0,0454 |
| Q83EV9 | coaA | 26,142 | 26,208 | 26,325 | 25,896 | 11 | 40 | 0,0293 |
| Q83EW0 | CBU_0198 | 26,915 | 25,964 | 26,881 | 26,499 | 8 | 45 | 0,0006 |
| Q83EW1 | CBU_0197 | 26,085 | 25,212 | 25,899 | 25,703 | 14 | 74 | 0,0003 |
| Q83EW3 | ampG | 23,415 | 23,298 | 24,240 | 24,199 | 3 | 18 | 0,0012 |
| Q83EW4 | sda | 24,711 | 25,497 | 24,391 | 23,742 | 4 | 31 | 0,0209 |
| Q83EW5 | CBU_0193 | 22,869 | 23,176 | 23,747 | 22,730 | 2 | 5 | 0,1027 |
| Q83EX6 | CBU_0182 | 23,020 | 23,555 | 23,657 | 22,998 | 3 | 12 | 0,0650 |
| Q83EX7 | tyrS | 28,610 | 28,571 | 28,474 | 28,366 | 19 | 198 | 0,0001 |
| Q83EX8 | CBU_0180 | 26,477 | 26,996 | 26,051 | 26,288 | 14 | 90 | 0,2752 |
| Q83EX9 | anmK | 26,393 | 26,277 | 25,917 | 25,735 | 9 | 83 | 0,0193 |
| Q83EY0 | CBU_0178 | 27,018 | 27,087 | 26,682 | 26,379 | 11 | 88 | 0,0007 |
| Q83EY1 | CBU_0177 | 27,107 | 26,971 | 26,681 | 26,359 | 11 | 86 | 0,0006 |
| Q83F01 | coaE | 25,843 | 24,997 | 25,259 | 24,919 | 6 | 32 | 0,0005 |
| Q83F02 | queF | 26,803 | 26,834 | 26,540 | 27,041 | 10 | 85 | 0,4066 |
| Q83F03 | zapD | 26,641 | 26,058 | 26,238 | 26,147 | 8 | 67 | 0,0228 |
| Q83F06 | secA | 29,399 | 29,466 | 29,406 | 29,314 | 30 | 332 | 0,0601 |
| Q83F11 | lpxC | 23,042 | 22,912 | 22,790 | 22,920 | 4 | 9 | 0,9830 |
| Q83F12 | ftsZ | 29,600 | 29,848 | 29,891 | 29,905 | 18 | 214 | 0,1249 |
| Q83F13 | ftsA | 28,459 | 28,370 | 28,817 | 28,578 | 16 | 204 | 0,0083 |
| Q83F14 | CBU_0139 | 23,866 | 24,521 | 24,470 | 24,257 | 3 | 18 | 0,0128 |
| Q83F16 | murB | 24,505 | 23,948 | 24,530 | 23,027 | 4 | 17 | 0,0051 |
| Q83F17 | murC | 26,393 | 26,486 | 26,779 | 26,481 | 11 | 63 | 0,0182 |
| Q83F20 | murD | 27,217 | 26,834 | 27,306 | 27,221 | 15 | 131 | 0,0013 |
| Q83F27 | murF | 27,540 | 27,830 | 27,897 | 27,891 | 14 | 138 | 0,0081 |
| Q83F28 | murE | 26,135 | 25,818 | 26,105 | 25,835 | 9 | 62 | 0,0044 |
| Q83F33 | ftsI | 26,022 | 25,862 | 25,568 | 25,604 | 12 | 40 | 0,0345 |
| Q83F35 | rsmH | 24,058 | 23,895 | 23,563 | 23,475 | 6 | 14 | 0,0166 |
| Q83F36 | mraZ | 28,110 | 27,559 | 27,649 | 27,139 | 9 | 86 | 0,0017 |
| Q83F37 | CBU_0114 | 28,538 | 27,862 | 28,585 | 28,298 | 12 | 142 | 0,0291 |
| Q83F38 | CBU_0113 | 23,703 | 24,172 | 25,264 | 25,653 | 2 | 14 | 0,0065 |
| Q83F39 | tdh | 26,406 | 26,581 | 26,555 | 26,720 | 6 | 45 | 0,2609 |
| Q83F40 | kbl | 27,591 | 27,920 | 27,767 | 27,706 | 13 | 118 | 0,0022 |
| Q83F42 | CBU_0109 | 27,727 | 28,036 | 27,665 | 27,639 | 7 | 68 | 0,0347 |
| Q83F43 | CBU_0108 | 22,564 | 23,114 | 23,180 | 22,867 | 2 | 10 | 0,2305 |
| Q83F44 | metN | 23,494 | 24,948 | 24,353 | 23,671 | 5 | 18 | 0,0122 |
| Q83F46 | CBU_0103 | 28,648 | 28,776 | 28,551 | 28,494 | 19 | 178 | 0,0008 |
| Q83F50 | sixA | 25,733 | 25,247 | 25,840 | 26,170 | 4 | 54 | 0,0015 |
| Q83F51 | nadC | 23,588 | 23,622 | 23,547 | 23,188 | 4 | 11 | 0,6434 |
| Q83F53 | clS | 23,650 | 24,255 | 23,674 | 23,957 | 3 | 16 | 0,2520 |
| Q83F54 | CBU_0095 | 24,324 | 24,603 | 24,743 | 24,873 | 2 | 13 | 0,0894 |
| Q83F55 | clpB | 30,487 | 30,488 | 30,345 | 30,386 | 41 | 514 | 0,7797 |
| Q83F56 | queE | 26,115 | 26,401 | 26,036 | 25,908 | 7 | 58 | 0,0032 |
| Q83F57 | ybgF | 29,565 | 29,321 | 29,688 | 29,220 | 16 | 158 | 0,3081 |
| Q83F58 | CBU_0091 | 29,867 | 29,771 | 29,423 | 29,347 | 9 | 134 | 0,0007 |
| Q83F59 | tolB | 28,319 | 28,048 | 28,203 | 28,125 | 16 | 164 | 0,2723 |
| Q83F61 | CBU_0087 | 27,132 | 25,261 | 26,697 | 26,924 | 15 | 66 | 0,0000 |
| Q83F64 | CBU_0084 | 28,852 | 28,073 | 28,104 | 28,457 | 13 | 142 | 0,0000 |
| Q83F67 | proS | 28,255 | 28,287 | 28,115 | 28,059 | 18 | 196 | 0,0283 |
| Q83F72 | visC | 26,281 | 26,340 | 26,478 | 26,534 | 10 | 78 | 0,0461 |
| Q83F73 | ubiH | 25,261 | 25,319 | 25,536 | 25,496 | 10 | 57 | 0,6358 |
| Q83F75 | CBU_0073 | 29,622 | 29,529 | 29,233 | 29,289 | 22 | 228 | 0,0007 |
| Q83F82 | CBU_0066 | 23,820 | 23,903 | 23,836 | 23,448 | 5 | 23 | 0,6979 |
| Q83F83 | CBU_0065 | 28,301 | 27,288 | 28,025 | 27,636 | 7 | 69 | 0,0010 |
| Q83F84 | parE | 27,802 | 27,851 | 27,744 | 27,651 | 19 | 146 | 0,1247 |
| Q83F85 | kdtA | 23,000 | 22,846 | 23,475 | 22,561 | 2 | 5 | 0,2079 |
| Q83F92 | CBU_0056 | 29,568 | 29,250 | 29,340 | 29,154 | 27 | 272 | 0,1497 |
| Q83F94 | ubiC | 26,631 | 26,473 | 26,412 | 26,531 | 9 | 69 | 0,1007 |
| Q83F97 | CBU_0051 | 25,296 | 25,763 | 25,924 | 26,261 | 8 | 50 | 0,0662 |
| Q83F98 | CBU_0050 | 23,200 | 25,022 | 23,847 | 25,172 | 4 | 15 | 0,0095 |
| Q83F99 | CBU_0049 | 23,478 | 22,344 | 23,192 | 22,878 | 3 | 16 | 0,0006 |
| Q83FA0 | CBU_0048 | 25,388 | 24,847 | 25,557 | 24,814 | 7 | 37 | 0,0003 |
| Q83FA1 | CBU_0045 | 29,984 | 29,779 | 29,824 | 29,716 | 14 | 172 | 0,0139 |
| Q83FA2 | CBU_0044 | 27,809 | 27,401 | 27,343 | 27,643 | 10 | 67 | 0,0034 |
| Q83FA3 | CBU_0043 | 26,983 | 26,961 | 26,566 | 25,964 | 7 | 62 | 0,0054 |
| Q83FA4 | hemH | 23,640 | 24,088 | 23,905 | 23,907 | 4 | 11 | 0,1015 |
| Q83FA6 | prlC | 29,673 | 29,871 | 29,486 | 29,430 | 22 | 246 | 0,0004 |
| Q83FA7 | CBU_0035 | 26,921 | 27,778 | 27,136 | 27,397 | 12 | 93 | 0,0000 |
| Q83FA8 | acpP | 23,226 | 25,452 | 25,857 | 27,865 | 3 | 19 | 0,0135 |
| Q83FB1 | CBU_0031 | 27,698 | 27,225 | 27,796 | 27,824 | 11 | 75 | 0,0001 |
| Q83FB2 | CBU_0029 | 23,354 | 22,664 | 23,486 | 23,889 | 2 | 3 | 0,1789 |
| Q83FB4 | rpiA | 23,550 | 23,291 | 23,937 | 22,388 | 2 | 17 | 0,0507 |
| Q83FB6 | csrA1 | 26,299 | 27,778 | 27,376 | 27,857 | 3 | 36 | 0,0003 |
| Q83FB7 | CBU_0023 | 24,368 | 24,480 | 24,658 | 24,978 | 5 | 37 | 0,0075 |
| Q83FB9 | CBU_0021 | 29,204 | 29,181 | 28,747 | 29,800 | 27 | 281 | 0,0000 |
| Q83FC0 | CBU_0020 | 26,508 | 25,488 | 25,690 | 24,968 | 5 | 29 | 0,0018 |
| Q83FC3 | deoC | 27,633 | 27,363 | 27,530 | 27,297 | 15 | 144 | 0,1047 |
| Q83FC4 | xapA | 26,824 | 26,839 | 26,723 | 26,530 | 11 | 87 | 0,0114 |
| Q83FC5 | CBU_0015 | 23,415 | 22,861 | 23,035 | 22,992 | 2 | 9 | 0,5129 |
| Q83FD0 | aroE | 26,392 | 26,286 | 26,512 | 26,374 | 11 | 65 | 0,4626 |
| Q83FD1 | dacB | 23,411 | 23,693 | 24,293 | 23,482 | 5 | 15 | 0,1416 |
| Q83FD3 | CBU_0007 | 26,004 | 25,148 | 23,415 | 23,838 | 2 | 13 | 0,1079 |
| Q83FD5 | gyrB | 28,863 | 29,185 | 29,086 | 29,133 | 30 | 261 | 0,0085 |
| Q83FD6 | recF | 24,744 | 24,420 | 24,388 | 24,739 | 4 | 10 | 0,1188 |
| Q83FD7 | dnaN | 29,813 | 29,852 | 30,161 | 30,239 | 13 | 198 | 0,0006 |
| Q83FD8 | dnaA | 24,141 | 26,198 | 25,591 | 25,793 | 12 | 67 | 0,0001 |
| Q9KI19 | rpoS | 25,707 | 22,888 | 25,913 | 23,331 | 7 | 21 | 0,0003 |
| Q9KI21 | surE | 26,476 | 26,795 | 26,554 | 26,420 | 7 | 51 | 0,1252 |
| Q9X5U8 | rpsB | 29,479 | 30,001 | 29,940 | 30,060 | 14 | 224 | 0,0011 |
| Q9X5U9 | tsf | 29,629 | 29,691 | 29,773 | 29,907 | 16 | 243 | 0,2409 |
| Q9ZH99 | icd | 30,977 | 31,242 | 30,979 | 31,276 | 32 | 419 | 0,0042 |

Table S2: LFQ analysis of all quantifiable proteins depicted as log2 average protein LFQ intensities (including values imputed by perseus-type value imputation) for each analyses in axenic media.

|  |  | **LFQ intensities (average)** | |  |  |  |
| --- | --- | --- | --- | --- | --- | --- |
| **Protein IDs** | **Gene name** | **L929 inf. Phase II** | **L929 inf. Phase I** | **Peptides** | **MS/MS Count** | **Student's t-test q-value** |
| B5QS73 | CBU_0089a | 27,571 | 27,440 | 6 | 66 | 0,9394 |
| B5QS96 | CBU_0516a | 22,246 | 21,261 | 4 | 8 | 0,3092 |
| B5QS99 | CBU_0562a | 24,169 | 24,118 | 6 | 37 | 0,9696 |
| Q45966;B5QSC0 | scvA | 22,176 | 23,290 | 1 | 28 | 0,4900 |
| B5QSE4 | CBU_1634a | 21,480 | 20,642 | 2 | 17 | 0,2614 |
| B5QSF7 | CBU_1764a | 22,657 | 21,998 | 4 | 9 | 0,6380 |
| B5QSG2 | CBU_1847b | 22,265 | 21,804 | 4 | 9 | 0,0878 |
| B5U8P9 | parB.1 | 22,539 | 23,120 | 10 | 20 | 0,3788 |
| B5U8Q0 | CBU_0677 | 23,445 | 21,061 | 10 | 16 | 0,0837 |
| B5U8Q4 | CBUA0028 | 21,146 | 20,615 | 3 | 8 | 0,8250 |
| B5U8Q7 | CBU_0700 | 21,675 | 20,935 | 2 | 3 | 0,7518 |
| H7C7C9 | parB.2 | 25,390 | 25,707 | 18 | 95 | 0,5508 |
| H7C7D5 | CBU_0702 | 21,616 | 21,234 | 6 | 16 | 0,0237 |
| H7C7D7 | com1 | 28,790 | 29,097 | 18 | 437 | 0,1970 |
| H7C7D8 | cysQ-2 | 22,723 | 22,009 | 13 | 40 | 0,2605 |
| H7C7D9 | CBU_1670 | 21,183 | 21,184 | 2 | 7 | 0,9785 |
| H7C7E7 | CBU_0676 | 24,867 | 20,320 | 13 | 43 | 0,0374 |
| H7C7F1 | CBUA0037 | 23,226 | 23,351 | 11 | 47 | 0,9793 |
| H7C7F5 | CBUA0013 | 21,788 | 21,308 | 6 | 12 | 0,1486 |
| H7C7G0 | CBUA0027 | 22,109 | 22,382 | 3 | 13 | 0,4341 |
| H7C7G1 | CBU_0678 | 22,053 | 21,082 | 4 | 7 | 0,0272 |
| H7C7G2 | rfbI | 21,692 | 20,864 | 7 | 18 | 0,0171 |
| O85387 | rplV | 23,973 | 23,965 | 5 | 25 | 0,9114 |
| O85388 | rpsC | 26,777 | 26,692 | 19 | 180 | 0,8950 |
| O87712 | dnaK | 28,724 | 29,156 | 47 | 678 | 0,4662 |
| P0C8S3 | rplL | 26,879 | 26,691 | 6 | 104 | 0,6503 |
| P0C8S4 | rpoB | 28,871 | 28,782 | 90 | 988 | 0,8885 |
| P18789 | gltA | 26,229 | 26,371 | 16 | 167 | 0,7254 |
| P19421 | groL | 31,278 | 31,654 | 59 | 1382 | 0,4170 |
| P19422 | groS | 29,208 | 29,542 | 12 | 205 | 0,6613 |
| P19685 | sodB | 28,246 | 28,096 | 13 | 235 | 0,7398 |
| P24703 | dapB | 21,786 | 21,727 | 5 | 25 | 0,9949 |
| P39916 | trxB | 24,525 | 24,819 | 9 | 116 | 0,4504 |
| P39917 | lolA | 25,189 | 25,766 | 11 | 93 | 0,0880 |
| P39918 | rarA | 21,869 | 21,961 | 9 | 25 | 0,9569 |
| P39919 | serS | 25,272 | 25,949 | 18 | 164 | 0,0075 |
| P39920 | ftsK | 24,407 | 24,576 | 23 | 91 | 0,3757 |
| P42381 | dnaJ | 22,847 | 23,274 | 12 | 70 | 0,7004 |
| P45650 | yidC | 23,862 | 24,061 | 10 | 51 | 0,4878 |
| P45651 | glyS | 24,775 | 24,938 | 26 | 156 | 0,4680 |
| P45680 | uspA2 | 25,781 | 25,568 | 6 | 79 | 0,2163 |
| P47849 | prfA | 21,211 | 21,530 | 7 | 20 | 0,8739 |
| P51053 | sdhB | 24,912 | 25,474 | 10 | 99 | 0,1351 |
| P51054 | sdhA | 26,199 | 26,404 | 18 | 171 | 0,3695 |
| P51056 | sucA | 26,676 | 26,919 | 45 | 325 | 0,4720 |
| P51752 | mip | 27,837 | 28,059 | 16 | 167 | 0,5664 |
| P51836 | era | 21,974 | 22,343 | 4 | 22 | 0,4177 |
| P51837 | rnc | 21,554 | 21,393 | 5 | 11 | 0,1637 |
| P53591 | sucD | 25,904 | 26,271 | 13 | 126 | 0,3398 |
| P53592 | sucC | 27,297 | 27,549 | 18 | 219 | 0,4961 |
| P59650 | CBU_0721 | 23,205 | 22,998 | 9 | 44 | 0,9409 |
| P59753 | rpsM | 25,852 | 25,704 | 8 | 91 | 0,8590 |
| P94612 | mnmE | 21,637 | 21,344 | 7 | 23 | 0,7471 |
| P94613 | mnmG | 20,846 | 20,077 | 2 | 16 | 0,4177 |
| P94616 | glyQ | 23,414 | 23,394 | 8 | 33 | 0,9679 |
| Q45881 | hcbA | 24,474 | 26,401 | 2 | 42 | 0,3686 |
| Q45918 | pyrE | 26,476 | 27,088 | 9 | 104 | 0,2574 |
| Q45920 | dut | 22,747 | 21,294 | 4 | 28 | 0,1315 |
| Q4AAX7 | truB | 20,986 | 21,301 | 4 | 17 | 0,9951 |
| Q4AAX8 | CBU_1059 | 21,817 | 21,981 | 7 | 12 | 0,7297 |
| Q4AAY0 | rluD | 22,548 | 22,779 | 8 | 33 | 0,6405 |
| Q81ZL2 | smc | 25,733 | 26,636 | 39 | 218 | 0,0674 |
| Q820B3 | rfbA | 25,138 | 23,748 | 10 | 46 | 0,0506 |
| Q820B4 | lnt | 22,554 | 23,365 | 3 | 35 | 0,0208 |
| Q820B5 | ubiG | 23,195 | 24,413 | 12 | 77 | 0,0324 |
| Q820B6 | CBU_0175 | 20,783 | 20,203 | 4 | 9 | 0,4739 |
| Q820V9 | cca | 22,195 | 22,290 | 9 | 32 | 0,5868 |
| Q820W0 | rsmI | 22,030 | 22,691 | 7 | 31 | 0,0672 |
| Q820W1 | CBU_1482 | 26,030 | 26,214 | 11 | 135 | 0,4829 |
| Q820W2 | mnmA | 21,052 | 21,104 | 3 | 19 | 0,3748 |
| Q820W3 | miaA | 21,347 | 21,648 | 8 | 14 | 0,6123 |
| Q820W4 | CBU_0847 | 22,216 | 22,472 | 4 | 31 | 0,4651 |
| Q820W5 | CBU_0744 | 22,024 | 23,227 | 6 | 40 | 0,0741 |
| Q820W6 | lpxA | 23,734 | 23,895 | 7 | 54 | 0,7631 |
| Q820W7 | fabZ | 24,295 | 24,363 | 7 | 45 | 0,9431 |
| Q820W8 | CBU_0610 | 24,230 | 23,729 | 6 | 34 | 0,1648 |
| Q820W9 | fabG | 24,948 | 24,379 | 7 | 91 | 0,0755 |
| Q820X0 | fabH | 24,270 | 24,113 | 8 | 65 | 0,7624 |
| Q820X1 | trmD | 21,639 | 22,003 | 4 | 21 | 0,3215 |
| Q820X2 | spoT | 26,365 | 27,107 | 32 | 291 | 0,1107 |
| Q820X3 | murG | 23,030 | 22,983 | 7 | 44 | 0,8773 |
| Q820X4 | CBU_0038 | 23,694 | 23,637 | 7 | 32 | 0,8692 |
| Q820X5 | fabA | 21,790 | 21,155 | 5 | 12 | 0,9586 |
| Q820X6 | CBU_0036 | 21,358 | 21,019 | 2 | 16 | 0,0487 |
| Q83A05 | CBUA0023 | 22,072 | 20,516 | 5 | 16 | 0,0551 |
| Q83A13 | CBUA0010 | 22,572 | 22,327 | 12 | 22 | 0,9918 |
| Q83A16 | pyrB | 22,884 | 23,164 | 8 | 26 | 0,2207 |
| Q83A18 | CBU_2093 | 21,442 | 21,135 | 4 | 23 | 0,3571 |
| Q83A19 | pckA | 27,116 | 27,221 | 21 | 249 | 0,4928 |
| Q83A20 | CBU_2091 | 22,729 | 23,540 | 6 | 32 | 0,4069 |
| Q83A21 | proC | 24,625 | 24,939 | 12 | 85 | 0,0182 |
| Q83A24 | trx | 26,393 | 26,640 | 8 | 95 | 0,3024 |
| Q83A25 | rho | 27,253 | 27,796 | 28 | 314 | 0,0698 |
| Q83A26 | CBU_2085 | 21,969 | 22,119 | 4 | 17 | 0,5886 |
| Q83A30 | hemY | 26,201 | 26,630 | 30 | 244 | 0,1770 |
| Q83A31 | CBU_2080 | 25,792 | 25,595 | 14 | 159 | 0,6990 |
| Q83A32 | CBU_2079 | 21,538 | 21,401 | 2 | 8 | 0,9987 |
| Q83A33 | CBU_2078 | 22,059 | 22,364 | 8 | 23 | 0,2867 |
| Q83A34 | hemD | 20,739 | 21,671 | 6 | 26 | 0,1164 |
| Q83A35 | CBU_2076 | 22,956 | 24,022 | 3 | 26 | 0,1476 |
| Q83A36 | aroD | 21,633 | 22,529 | 3 | 17 | 0,0188 |
| Q83A37 | hemC | 22,499 | 23,243 | 8 | 41 | 0,0368 |
| Q83A38 | CBU_2073 | 22,574 | 21,625 | 4 | 22 | 0,3368 |
| Q83A39 | CBU_2072 | 23,248 | 24,473 | 4 | 23 | 0,1164 |
| Q83A41 | CBU_2070 | 24,189 | 24,665 | 9 | 44 | 0,3889 |
| Q83A42 | tdk | 22,578 | 22,779 | 5 | 35 | 0,6694 |
| Q83A43 | CBU_2068 | 21,203 | 21,301 | 2 | 7 | 0,8570 |
| Q83A56 | uvrD | 25,497 | 23,502 | 12 | 27 | 0,0323 |
| Q83A61 | trpS | 22,188 | 22,392 | 5 | 26 | 0,6774 |
| Q83A62 | metE | 22,689 | 21,099 | 11 | 29 | 0,2960 |
| Q83A77 | ahcY | 25,788 | 25,862 | 20 | 188 | 0,9564 |
| Q83A78 | metK | 25,863 | 25,435 | 18 | 172 | 0,6733 |
| Q83A79 | CBU_2029 | 27,958 | 28,209 | 11 | 169 | 0,4564 |
| Q83A83 | metC | 23,047 | 23,177 | 10 | 50 | 0,8481 |
| Q83A84 | CBU_2024 | 23,377 | 23,789 | 7 | 51 | 0,0176 |
| Q83A85 | CBU_2023 | 24,110 | 24,629 | 7 | 37 | 0,1381 |
| Q83A88 | ubiB | 22,923 | 22,994 | 13 | 49 | 0,9704 |
| Q83A89 | CBU_2018 | 20,712 | 20,832 | 3 | 22 | 0,9458 |
| Q83A90 | ubiE | 23,247 | 23,838 | 10 | 52 | 0,1308 |
| Q83A94 | hslU | 26,756 | 26,857 | 35 | 262 | 0,8846 |
| Q83A95 | hslV | 24,532 | 24,555 | 5 | 81 | 0,9689 |
| Q83A97 | CBU_2009 | 26,222 | 26,344 | 25 | 185 | 0,8753 |
| Q83A98 | argS | 24,886 | 25,496 | 21 | 133 | 0,0237 |
| Q83AA0 | CBU_2006 | 21,406 | 21,244 | 3 | 5 | 0,8518 |
| Q83AA1 | CBU_2004 | 22,801 | 23,007 | 9 | 38 | 0,4935 |
| Q83AA3 | purE | 23,291 | 23,368 | 4 | 41 | 0,7287 |
| Q83AA5 | topA | 24,076 | 24,274 | 24 | 93 | 0,5902 |
| Q83AA8 | fmt | 23,423 | 24,318 | 8 | 51 | 0,1402 |
| Q83AA9 | CBU_1996 | 23,701 | 23,437 | 9 | 55 | 0,3866 |
| Q83AB0 | acyP | 21,998 | 21,922 | 3 | 13 | 0,6996 |
| Q83AB2 | folA | 23,530 | 24,166 | 5 | 53 | 0,0585 |
| Q83AB7 | apaH | 23,626 | 22,395 | 5 | 20 | 0,3162 |
| Q83AB8 | sppA | 25,673 | 25,301 | 11 | 127 | 0,1552 |
| Q83AC0 | CBU_1984 | 25,076 | 24,489 | 4 | 34 | 0,6495 |
| Q83AC1 | uspA1 | 24,328 | 24,824 | 6 | 66 | 0,1404 |
| Q83AC2 | rsmA | 22,472 | 23,537 | 10 | 53 | 0,0464 |
| Q83AC3 | pdxA | 23,627 | 24,293 | 9 | 42 | 0,0491 |
| Q83AC4 | CBU_1980 | 27,519 | 27,022 | 14 | 192 | 0,3019 |
| Q83AC6 | lptD | 22,254 | 22,336 | 9 | 47 | 0,7072 |
| Q83AC7 | CBU_1977 | 20,844 | 19,990 | 7 | 16 | 0,0210 |
| Q83AC9 | CBU_1975 | 24,267 | 24,690 | 7 | 89 | 0,1550 |
| Q83AD4 | dapF | 25,206 | 25,767 | 13 | 93 | 0,0236 |
| Q83AD5 | dksA | 24,581 | 24,362 | 6 | 44 | 0,6972 |
| Q83AD8 | prmC | 21,090 | 20,691 | 6 | 20 | 0,6345 |
| Q83AE6 | pntAA | 23,216 | 22,442 | 13 | 50 | 0,4185 |
| Q83AE7 | CBU_1954 | 22,910 | 21,972 | 9 | 54 | 0,1760 |
| Q83AF3 | glmU | 25,074 | 25,541 | 19 | 158 | 0,0430 |
| Q83AF4 | atpC | 22,222 | 20,606 | 2 | 15 | 0,1487 |
| Q83AF5 | atpD | 26,863 | 26,882 | 24 | 241 | 0,9791 |
| Q83AF6 | atpG | 24,060 | 24,030 | 9 | 82 | 0,9628 |
| Q83AF7 | atpA | 27,504 | 27,345 | 28 | 309 | 0,7106 |
| Q83AF8 | atpH | 21,988 | 21,928 | 7 | 65 | 0,9702 |
| Q83AF9 | atpF | 28,073 | 27,987 | 10 | 94 | 0,8810 |
| Q83AG0 | atpE | 22,470 | 20,924 | 2 | 26 | 0,0487 |
| Q83AG6 | ku | 21,055 | 21,124 | 4 | 6 | 0,8401 |
| Q83AH1 | CBU_1928 | 21,467 | 21,268 | 11 | 22 | 0,8258 |
| Q83AH2 | parB | 23,221 | 23,851 | 14 | 62 | 0,4669 |
| Q83AH3 | parA | 22,574 | 23,386 | 7 | 29 | 0,1546 |
| Q83AI3 | ftsY | 22,464 | 22,455 | 7 | 35 | 0,9799 |
| Q83AI4 | CBU_1902 | 25,531 | 26,020 | 15 | 133 | 0,0329 |
| Q83AI5 | CBU_1901 | 25,923 | 26,659 | 18 | 188 | 0,0161 |
| Q83AJ2 | aroB | 22,796 | 23,211 | 9 | 55 | 0,0474 |
| Q83AJ8 | ponA | 23,953 | 23,759 | 17 | 80 | 0,1603 |
| Q83AK2 | queD | 23,671 | 23,837 | 8 | 58 | 0,5607 |
| Q83AK3 | hemL | 22,591 | 22,350 | 8 | 22 | 0,9584 |
| Q83AK6 | def2 | 23,392 | 23,115 | 4 | 20 | 0,7304 |
| Q83AK7 | erpA | 21,614 | 21,844 | 3 | 22 | 0,8265 |
| Q83AK8 | CBU_1877 | 23,060 | 23,400 | 14 | 58 | 0,3638 |
| Q83AL0 | gshB | 26,259 | 29,191 | 5 | 42 | 0,3784 |
| Q83AL1 | CBU_1874 | 25,555 | 26,042 | 17 | 179 | 0,0903 |
| Q83AL2 | rpe | 24,511 | 24,748 | 8 | 50 | 0,4056 |
| Q83AL3 | coq7 | 21,190 | 21,485 | 3 | 9 | 0,7428 |
| Q83AL4 | CBU_1869 | 24,280 | 24,974 | 10 | 68 | 0,1765 |
| Q83AL7 | parC | 25,702 | 26,109 | 23 | 210 | 0,0323 |
| Q83AL8 | CBU_1865 | 21,741 | 22,765 | 7 | 17 | 0,2690 |
| Q83AM0 | CBU_1863 | 22,163 | 21,320 | 4 | 12 | 0,4052 |
| Q83AM1 | CBU_1862 | 21,417 | 20,887 | 7 | 10 | 0,3135 |
| Q83AM7 | CBU_1856 | 22,765 | 22,555 | 5 | 17 | 0,8255 |
| Q83AN5 | CBU_1847 | 23,003 | 22,721 | 4 | 22 | 0,8088 |
| Q83AN9 | ychF | 22,005 | 22,331 | 9 | 24 | 0,8002 |
| Q83AP0 | pth | 22,208 | 21,868 | 7 | 25 | 0,4573 |
| Q83AP1 | rplY | 25,268 | 25,078 | 8 | 60 | 0,6982 |
| Q83AP3 | rfbC | 23,074 | 23,063 | 8 | 30 | 0,9682 |
| Q83AP4 | CBU_1837 | 25,147 | 25,083 | 15 | 62 | 0,9573 |
| Q83AP5 | CBU_1836 | 24,414 | 24,453 | 11 | 84 | 0,9790 |
| Q83AP6 | CBU_1835 | 25,096 | 25,013 | 21 | 166 | 0,9223 |
| Q83AQ1 | prs | 25,013 | 25,092 | 14 | 96 | 0,8733 |
| Q83AQ2 | lolB | 21,145 | 21,655 | 4 | 15 | 0,9775 |
| Q83AQ4 | psd | 22,173 | 22,876 | 6 | 22 | 0,3685 |
| Q83AQ8 | sodC | 26,587 | 26,995 | 7 | 98 | 0,4734 |
| Q83AQ9 | CBU_1821 | 20,906 | 20,883 | 3 | 12 | 0,6536 |
| Q83AR0 | CBU_1820 | 21,603 | 21,503 | 7 | 36 | 0,8287 |
| Q83AR3 | CBU_1817 | 21,597 | 21,659 | 3 | 14 | 0,4718 |
| Q83AR4 | efp | 25,288 | 25,295 | 7 | 45 | 0,9940 |
| Q83AR8 | pdxB | 21,109 | 21,621 | 6 | 30 | 0,1624 |
| Q83AS0 | macA | 24,527 | 24,653 | 12 | 81 | 0,3896 |
| Q83AS1 | CBU_1809 | 21,878 | 22,334 | 5 | 15 | 0,3885 |
| Q83AT2 | CBU_1798 | 22,216 | 22,566 | 6 | 24 | 0,1473 |
| Q83AT3 | CBU_1797 | 25,270 | 24,414 | 3 | 16 | 0,1019 |
| Q83AT5 | polI | 24,882 | 25,771 | 33 | 183 | 0,0184 |
| Q83AU0 | CBU_1789 | 26,010 | 26,364 | 13 | 84 | 0,5915 |
| Q83AU2 | glmS | 24,778 | 24,989 | 24 | 102 | 0,0887 |
| Q83AU3 | CBU_1786 | 20,944 | 20,943 | 5 | 11 | 0,7609 |
| Q83AU4 | tkt | 25,569 | 25,533 | 21 | 121 | 0,9344 |
| Q83AU5 | gap | 27,893 | 27,837 | 22 | 274 | 0,9225 |
| Q83AU6 | pgk | 25,526 | 25,616 | 14 | 151 | 0,7443 |
| Q83AU7 | pyk | 26,210 | 26,350 | 19 | 140 | 0,4620 |
| Q83AV0 | fbaA | 27,423 | 27,894 | 18 | 215 | 0,2333 |
| Q83AV6 | engB | 24,721 | 25,170 | 7 | 66 | 0,1507 |
| Q83AV8 | CBU_1770 | 25,833 | 25,999 | 16 | 145 | 0,4454 |
| Q83AW1 | feoA | 22,153 | 22,856 | 4 | 20 | 0,7479 |
| Q83AW2 | feoB | 26,896 | 27,084 | 24 | 182 | 0,8349 |
| Q83AW7 | CBU_1761 | 24,237 | 24,720 | 26 | 118 | 0,0949 |
| Q83AX3 | CBU_1754 | 24,851 | 25,018 | 10 | 111 | 0,6132 |
| Q83AX5 | CBU_1752 | 24,563 | 23,898 | 29 | 148 | 0,5441 |
| Q83AX6 | CBU_1751 | 25,657 | 25,538 | 25 | 131 | 0,8450 |
| Q83AX8 | rplM | 24,949 | 24,731 | 7 | 54 | 0,6995 |
| Q83AX9 | rpsI | 24,695 | 24,472 | 3 | 60 | 0,6718 |
| Q83AY0 | sspA | 24,763 | 24,563 | 8 | 92 | 0,7461 |
| Q83AY2 | CBU_1745 | 22,406 | 22,576 | 4 | 20 | 0,8957 |
| Q83AY3 | CBU_1744 | 24,825 | 25,190 | 6 | 38 | 0,0549 |
| Q83AY4 | gmhA | 23,466 | 23,574 | 9 | 48 | 0,8012 |
| Q83AY6 | CBU_1741 | 25,611 | 26,159 | 14 | 155 | 0,0181 |
| Q83AY8 | hipB | 23,588 | 23,755 | 6 | 20 | 0,9394 |
| Q83AY9 | purN | 22,206 | 23,774 | 9 | 29 | 0,0669 |
| Q83AZ0 | purM | 24,460 | 25,100 | 11 | 77 | 0,0179 |
| Q83AZ1 | CBU_1735 | 24,322 | 24,975 | 7 | 52 | 0,1645 |
| Q83AZ2 | CBU_1734 | 21,913 | 23,200 | 8 | 43 | 0,0186 |
| Q83AZ4 | CBU_1732 | 21,248 | 20,807 | 5 | 14 | 0,2353 |
| Q83AZ5 | CBU_1730 | 20,932 | 21,741 | 6 | 10 | 0,4919 |
| Q83AZ6 | hemF | 22,115 | 22,147 | 6 | 19 | 0,6337 |
| Q83AZ8 | arcB | 22,706 | 23,350 | 11 | 34 | 0,2755 |
| Q83AZ9 | accC | 24,998 | 25,009 | 11 | 109 | 0,9787 |
| Q83B00 | accB | 24,213 | 24,125 | 6 | 36 | 0,8787 |
| Q83B02 | dsbD | 23,889 | 24,041 | 7 | 60 | 0,4944 |
| Q83B04 | CBU_1721 | 20,920 | 21,180 | 3 | 10 | 0,6480 |
| Q83B05 | acnA | 26,719 | 26,905 | 32 | 309 | 0,6416 |
| Q83B06 | gcvT | 24,184 | 24,398 | 11 | 92 | 0,3569 |
| Q83B07 | gcvH | 25,352 | 25,083 | 6 | 57 | 0,5882 |
| Q83B08 | gcvPA | 23,327 | 24,107 | 7 | 48 | 0,0692 |
| Q83B09 | gcvPB | 24,340 | 24,528 | 14 | 85 | 0,3577 |
| Q83B14 | CBU_1706 | 29,642 | 29,832 | 16 | 373 | 0,4727 |
| Q83B15 | CBU_1705 | 25,612 | 26,212 | 3 | 43 | 0,5904 |
| Q83B17 | pyrC | 22,983 | 23,175 | 9 | 37 | 0,3389 |
| Q83B21 | CBU_1698 | 21,999 | 22,510 | 8 | 17 | 0,8522 |
| Q83B24 | metG | 24,215 | 24,299 | 15 | 85 | 0,5958 |
| Q83B28 | CBU_1691 | 23,154 | 23,525 | 4 | 37 | 0,5202 |
| Q83B36 | pyrG | 25,431 | 25,690 | 19 | 145 | 0,5605 |
| Q83B40 | speG | 24,292 | 24,547 | 7 | 47 | 0,5795 |
| Q83B41 | CBU_1677 | 23,560 | 24,263 | 11 | 88 | 0,5088 |
| Q83B43 | kdsA | 22,293 | 23,416 | 6 | 22 | 0,2904 |
| Q83B44 | eno | 25,943 | 26,314 | 19 | 185 | 0,0708 |
| Q83B51 | CBU_1664 | 24,759 | 25,073 | 9 | 86 | 0,0705 |
| Q83B53 | CBU_1662 | 23,047 | 22,322 | 2 | 10 | 0,5861 |
| Q83B54 | rfaF | 22,693 | 23,997 | 10 | 72 | 0,2606 |
| Q83B58 | CBU_1657 | 21,380 | 21,692 | 3 | 25 | 0,0845 |
| Q83B60 | hldE | 22,535 | 21,392 | 14 | 34 | 0,3776 |
| Q83B63 | icmX | 27,860 | 28,378 | 22 | 255 | 0,1380 |
| Q83B64 | CBU_1651 | 25,127 | 25,481 | 7 | 90 | 0,1045 |
| Q83B65 | icmW | 22,326 | 23,360 | 4 | 24 | 0,2208 |
| Q83B66 | icmV | 22,848 | 22,110 | 4 | 26 | 0,3023 |
| Q83B67 | dotaA | 23,701 | 23,704 | 13 | 106 | 0,9978 |
| Q83B70 | dotB | 24,766 | 25,152 | 22 | 114 | 0,1547 |
| Q83B71 | dotC | 26,015 | 26,490 | 12 | 173 | 0,2453 |
| Q83B72 | dotD | 25,466 | 25,642 | 11 | 72 | 0,6413 |
| Q83B73 | icmS | 22,829 | 23,011 | 4 | 30 | 0,6426 |
| Q83B75 | CBU_1638 | 21,299 | 21,648 | 8 | 19 | 0,3141 |
| Q83B79 | icmQ | 22,132 | 21,677 | 7 | 28 | 0,6062 |
| Q83B80 | icmP | 22,513 | 22,310 | 8 | 43 | 0,6963 |
| Q83B81 | icmO | 23,119 | 23,320 | 14 | 63 | 0,4177 |
| Q83B82 | icmN | 25,042 | 25,483 | 10 | 82 | 0,3788 |
| Q83B83 | icmL.2 | 23,521 | 24,124 | 7 | 37 | 0,4193 |
| Q83B84 | icmL.1 | 23,442 | 24,162 | 8 | 45 | 0,0934 |
| Q83B85 | icmK | 24,848 | 25,337 | 9 | 82 | 0,3216 |
| Q83B86 | icmE | 27,231 | 28,532 | 43 | 346 | 0,0340 |
| Q83B87 | icmG | 24,774 | 25,825 | 12 | 85 | 0,0671 |
| Q83B90 | icmJ | 22,087 | 22,619 | 7 | 26 | 0,3772 |
| Q83B91 | icmB | 26,240 | 26,941 | 32 | 260 | 0,0835 |
| Q83BA9 | CBU_1603 | 20,900 | 20,842 | 3 | 9 | 0,3584 |
| Q83BB0 | rimK | 23,480 | 24,897 | 8 | 22 | 0,3693 |
| Q83BB6 | rpoD | 25,788 | 25,775 | 16 | 145 | 0,9693 |
| Q83BB7 | dnaG | 22,918 | 22,932 | 11 | 47 | 0,9685 |
| Q83BB8 | CBU_1594 | 24,738 | 24,841 | 5 | 50 | 0,5279 |
| Q83BC2 | CBU_1589 | 23,642 | 22,703 | 2 | 26 | 0,4333 |
| Q83BD2 | CBU_1579 | 21,550 | 23,139 | 6 | 28 | 0,0344 |
| Q83BD6 | tolR | 21,588 | 21,843 | 2 | 8 | 0,4816 |
| Q83BD7 | tolQ | 22,703 | 23,206 | 7 | 32 | 0,1230 |
| Q83BD8 | CBU_1573 | 22,131 | 22,531 | 4 | 26 | 0,7629 |
| Q83BE0 | ruvB | 25,550 | 25,617 | 17 | 143 | 0,9393 |
| Q83BE4 | CBU_1566 | 22,420 | 22,828 | 4 | 21 | 0,0588 |
| Q83BE5 | aspS | 26,403 | 26,260 | 23 | 217 | 0,5047 |
| Q83BF1 | CBU_1559 | 22,698 | 22,907 | 5 | 35 | 0,1910 |
| Q83BF2 | CBU_1558 | 23,912 | 24,456 | 5 | 44 | 0,6621 |
| Q83BF5 | nrdB | 22,872 | 23,187 | 5 | 29 | 0,7483 |
| Q83BF6 | nrdA | 24,625 | 26,552 | 26 | 203 | 0,2813 |
| Q83BF8 | rppH | 21,051 | 22,026 | 3 | 10 | 0,1295 |
| Q83BF9 | ptsP | 24,474 | 24,717 | 13 | 107 | 0,4193 |
| Q83BG1 | CBU_1548 | 22,532 | 20,761 | 2 | 15 | 0,3473 |
| Q83BG2 | thyA | 21,900 | 21,665 | 5 | 16 | 0,3677 |
| Q83BH0 | CBU_1538 | 26,315 | 26,195 | 20 | 178 | 0,8626 |
| Q83BH1 | CBU_1537 | 21,078 | 20,662 | 2 | 7 | 0,3368 |
| Q83BH2 | gpmI | 24,370 | 24,657 | 18 | 91 | 0,0585 |
| Q83BH9 | CBU_1529 | 22,834 | 23,351 | 15 | 56 | 0,3704 |
| Q83BI7 | CBU_1521 | 25,366 | 25,900 | 4 | 62 | 0,0765 |
| Q83BI8 | grxC | 25,264 | 25,786 | 8 | 63 | 0,2345 |
| Q83BI9 | secB | 26,342 | 26,745 | 6 | 92 | 0,0527 |
| Q83BJ0 | gpsA | 24,399 | 24,923 | 7 | 77 | 0,0899 |
| Q83BJ5 | CBU_1513 | 23,374 | 23,428 | 8 | 46 | 0,9399 |
| Q83BJ8 | accA | 25,546 | 25,750 | 13 | 139 | 0,0850 |
| Q83BJ9 | tilS | 21,631 | 22,174 | 7 | 39 | 0,0172 |
| Q83BK0 | CBU_1508 | 22,375 | 22,388 | 11 | 44 | 0,9777 |
| Q83BK1 | CBU_1507 | 21,079 | 21,362 | 5 | 19 | 0,8325 |
| Q83BK2 | CBU_1506 | 21,067 | 21,560 | 3 | 10 | 0,1532 |
| Q83BK3 | lepA | 23,695 | 23,938 | 14 | 75 | 0,1890 |
| Q83BK4 | lepB-2 | 22,254 | 23,371 | 7 | 42 | 0,0884 |
| Q83BL1 | pdxJ | 23,916 | 24,412 | 4 | 39 | 0,2118 |
| Q83BL6 | gltX2 | 25,904 | 25,940 | 28 | 228 | 0,8743 |
| Q83BL7 | cysS | 23,622 | 23,761 | 18 | 98 | 0,8362 |
| Q83BM1 | CBU_1483 | 21,997 | 23,124 | 6 | 16 | 0,0210 |
| Q83BM7 | oxyR | 21,056 | 21,532 | 6 | 19 | 0,6924 |
| Q83BM8 | gatB | 25,499 | 25,325 | 22 | 155 | 0,3689 |
| Q83BM9 | gatA | 25,360 | 25,309 | 17 | 154 | 0,8581 |
| Q83BN0 | gatC | 22,688 | 23,005 | 4 | 18 | 0,6539 |
| Q83BN2 | mreB | 28,143 | 28,182 | 21 | 269 | 0,8833 |
| Q83BN3 | mreC | 23,245 | 24,311 | 9 | 75 | 0,1604 |
| Q83BN5 | CBU_1468 | 21,405 | 22,651 | 12 | 36 | 0,0370 |
| Q83BN6 | tldD | 21,624 | 22,729 | 10 | 36 | 0,0752 |
| Q83BN9 | hupB | 27,072 | 26,819 | 5 | 144 | 0,5925 |
| Q83BP5 | CBU_1458 | 21,900 | 22,073 | 8 | 36 | 0,1131 |
| Q83BQ2 | CBU_1451 | 25,882 | 25,685 | 15 | 97 | 0,5877 |
| Q83BQ7 | nuoC | 23,523 | 23,682 | 10 | 70 | 0,8168 |
| Q83BQ8 | nuoD | 24,288 | 24,567 | 12 | 100 | 0,4336 |
| Q83BQ9 | nuoE | 22,392 | 22,561 | 4 | 27 | 0,7991 |
| Q83BR0 | nuoF | 23,170 | 23,677 | 10 | 39 | 0,5008 |
| Q83BR1 | nuoG | 25,287 | 25,702 | 20 | 192 | 0,3302 |
| Q83BR3 | nuoI | 23,075 | 23,365 | 7 | 46 | 0,3989 |
| Q83BR6 | nuoL | 21,561 | 20,961 | 4 | 16 | 0,2432 |
| Q83BS0 | nusA | 26,625 | 26,599 | 25 | 245 | 0,9682 |
| Q83BS1 | infB | 26,475 | 26,359 | 30 | 250 | 0,6560 |
| Q83BS2 | rbfA | 21,412 | 21,005 | 5 | 16 | 0,9530 |
| Q83BS7 | CBU_1425 | 28,043 | 28,324 | 4 | 86 | 0,1137 |
| Q83BS8 | hemB | 23,840 | 24,186 | 11 | 79 | 0,6162 |
| Q83BT0 | radA | 22,945 | 22,357 | 13 | 49 | 0,6478 |
| Q83BT3 | glyA | 25,323 | 25,143 | 16 | 93 | 0,3901 |
| Q83BT4 | nrdR | 23,385 | 23,373 | 4 | 31 | 0,9698 |
| Q83BT5 | nusB | 22,815 | 22,139 | 6 | 46 | 0,6680 |
| Q83BT6 | CBU_1416 | 22,279 | 22,547 | 6 | 10 | 0,6472 |
| Q83BT7 | thiL | 24,248 | 25,059 | 13 | 113 | 0,0552 |
| Q83BU6 | CBU_1404 | 23,863 | 24,710 | 6 | 26 | 0,1635 |
| Q83BU7 | sucB | 27,245 | 27,432 | 19 | 222 | 0,2838 |
| Q83BU9 | enhA.5 | 22,668 | 24,221 | 4 | 21 | 0,2327 |
| Q83BV1 | map | 22,829 | 23,075 | 5 | 21 | 0,2850 |
| Q83BV3 | pyrH | 23,999 | 24,115 | 7 | 75 | 0,6517 |
| Q83BV4 | frr | 25,609 | 25,606 | 9 | 74 | 0,9972 |
| Q83BV7 | CBU_1380 | 24,485 | 24,803 | 5 | 66 | 0,6621 |
| Q83BW2 | relA | 22,136 | 22,001 | 6 | 32 | 0,5046 |
| Q83BW3 | CBU_1374 | 23,421 | 23,244 | 7 | 38 | 0,2591 |
| Q83BW4 | pabB | 22,970 | 23,670 | 16 | 60 | 0,3422 |
| Q83BW5 | CBU_1372 | 21,991 | 21,901 | 3 | 22 | 0,7587 |
| Q83BX5 | czcD.2 | 23,391 | 22,980 | 7 | 36 | 0,1360 |
| Q83BX7 | sufB | 21,659 | 21,713 | 5 | 22 | 0,2455 |
| Q83BX8 | sufC | 23,418 | 23,232 | 9 | 54 | 0,4894 |
| Q83BY0 | csdB | 23,253 | 23,276 | 9 | 41 | 0,9266 |
| Q83BY1 | iscU.2 | 21,167 | 20,782 | 3 | 7 | 0,2338 |
| Q83BY5 | ftsH | 27,155 | 27,363 | 31 | 336 | 0,3983 |
| Q83BY6 | folP | 23,653 | 23,746 | 12 | 46 | 0,8723 |
| Q83BY7 | glmM | 23,064 | 23,043 | 9 | 43 | 0,9403 |
| Q83BZ5 | guaB | 25,116 | 25,147 | 25 | 179 | 0,9437 |
| Q83BZ6 | guaA | 24,282 | 23,876 | 19 | 96 | 0,5600 |
| Q83BZ9 | ddl | 24,917 | 24,938 | 14 | 93 | 0,9634 |
| Q83C00 | dnaE | 25,734 | 24,769 | 26 | 72 | 0,0864 |
| Q83C10 | thrS | 26,635 | 26,328 | 33 | 215 | 0,2038 |
| Q83C11 | infC | 25,404 | 25,354 | 12 | 72 | 0,9441 |
| Q83C12 | rpmI | 20,378 | 19,637 | 1 | 6 | 0,4179 |
| Q83C13 | rplT | 23,082 | 22,262 | 3 | 20 | 0,4068 |
| Q83C14 | pheS | 23,454 | 23,678 | 7 | 29 | 0,1417 |
| Q83C15 | pheT | 25,122 | 25,177 | 25 | 156 | 0,7914 |
| Q83C16 | ihfA | 27,250 | 27,908 | 10 | 161 | 0,0486 |
| Q83C26 | CBU_1308 | 21,336 | 20,226 | 9 | 38 | 0,2177 |
| Q83C28 | msrA | 21,808 | 21,983 | 6 | 24 | 0,2911 |
| Q83C29 | smpB | 20,927 | 21,230 | 2 | 9 | 0,7892 |
| Q83C32 | omlA | 22,840 | 21,835 | 5 | 13 | 0,4581 |
| Q83C37 | recN | 23,666 | 24,203 | 22 | 106 | 0,6367 |
| Q83C38 | nadK | 24,206 | 24,821 | 9 | 81 | 0,0182 |
| Q83C41 | grpE | 25,158 | 24,634 | 5 | 47 | 0,3263 |
| Q83C42 | CBU_1292 | 21,242 | 21,573 | 8 | 22 | 0,4659 |
| Q83C43 | CBU_1291 | 21,407 | 21,175 | 3 | 11 | 0,9558 |
| Q83C46 | CBU_1286 | 20,960 | 20,896 | 5 | 17 | 0,4453 |
| Q83C49 | carA | 22,215 | 23,477 | 10 | 39 | 0,0951 |
| Q83C50 | carB | 24,763 | 25,179 | 24 | 138 | 0,0330 |
| Q83C51 | greA | 24,042 | 24,335 | 5 | 45 | 0,5826 |
| Q83C53 | CBU_1278 | 24,402 | 24,225 | 11 | 80 | 0,3781 |
| Q83C54 | eda | 23,565 | 23,680 | 8 | 42 | 0,7492 |
| Q83C55 | CBU_1276 | 25,701 | 25,766 | 13 | 136 | 0,7359 |
| Q83C56 | rspA | 27,026 | 27,036 | 21 | 238 | 0,9719 |
| Q83C57 | CBU_1274 | 21,412 | 22,310 | 4 | 17 | 0,1425 |
| Q83C58 | pfp | 24,592 | 24,364 | 17 | 138 | 0,8240 |
| Q83C60 | CBU_1269 | 24,859 | 25,347 | 12 | 73 | 0,4271 |
| Q83C61 | CBU_1268 | 25,712 | 26,185 | 19 | 165 | 0,1014 |
| Q83C62 | CBU_1267 | 22,537 | 22,789 | 11 | 36 | 0,6113 |
| Q83C63 | lipA | 22,658 | 23,710 | 9 | 41 | 0,1998 |
| Q83C68 | CBU_1261 | 26,665 | 26,901 | 19 | 210 | 0,4533 |
| Q83C69 | CBU_1260 | 26,960 | 28,055 | 13 | 209 | 0,0885 |
| Q83C70 | nhaP.1 | 21,977 | 23,320 | 7 | 20 | 0,0863 |
| Q83C71 | ndk | 26,144 | 25,991 | 6 | 94 | 0,8034 |
| Q83C74 | CBU_1255 | 22,958 | 23,507 | 4 | 36 | 0,0950 |
| Q83C77 | rlmN | 21,839 | 21,741 | 7 | 20 | 0,7482 |
| Q83C79 | CBU_1249 | 25,422 | 25,879 | 9 | 116 | 0,1159 |
| Q83C80 | hisS | 24,703 | 25,203 | 15 | 120 | 0,0153 |
| Q83C81 | CBU_1247 | 22,033 | 21,922 | 2 | 30 | 0,7553 |
| Q83C83 | der | 24,463 | 25,213 | 19 | 118 | 0,0186 |
| Q83C85 | xseA | 21,298 | 21,378 | 7 | 27 | 0,6952 |
| Q83C87 | mdh | 26,593 | 26,898 | 15 | 187 | 0,2073 |
| Q83C88 | tsaD | 21,980 | 22,453 | 5 | 18 | 0,6989 |
| Q83C90 | CBU_1238 | 21,231 | 20,387 | 2 | 7 | 0,5916 |
| Q83C93 | orn | 23,072 | 23,335 | 8 | 53 | 0,7430 |
| Q83C94 | CBU_1234 | 23,076 | 23,178 | 12 | 55 | 0,9286 |
| Q83C95 | CBU_1233 | 24,793 | 25,203 | 13 | 84 | 0,5829 |
| Q83C98 | CBU_1230 | 21,977 | 22,394 | 11 | 15 | 0,3002 |
| Q83CA0 | qseC | 20,915 | 21,349 | 3 | 15 | 0,5323 |
| Q83CA1 | qseB | 25,571 | 27,095 | 14 | 166 | 0,0342 |
| Q83CA2 | CBU_1226 | 27,459 | 27,707 | 65 | 470 | 0,5621 |
| Q83CA3 | CBU_1225 | 21,599 | 21,664 | 2 | 8 | 0,8248 |
| Q83CA4 | CBU_1224 | 23,281 | 24,120 | 14 | 70 | 0,0206 |
| Q83CA5 | kdgK | 24,818 | 24,847 | 11 | 69 | 0,9410 |
| Q83CA6 | dapA | 23,827 | 24,291 | 7 | 63 | 0,2176 |
| Q83CA7 | CBU_1221 | 26,705 | 27,029 | 10 | 127 | 0,2179 |
| Q83CA8 | purC | 25,143 | 25,157 | 9 | 93 | 0,9764 |
| Q83CC3 | CBU_1204 | 25,834 | 26,285 | 17 | 131 | 0,2691 |
| Q83CC4 | CBU_1203 | 21,616 | 22,158 | 10 | 22 | 0,1549 |
| Q83CC6 | queA | 21,281 | 20,836 | 3 | 14 | 0,5795 |
| Q83CD0 | clpA | 21,517 | 21,437 | 16 | 38 | 0,9290 |
| Q83CD1 | infA | 22,121 | 22,238 | 3 | 21 | 0,8292 |
| Q83CD5 | uvrC | 23,549 | 24,207 | 16 | 58 | 0,0593 |
| Q83CD7 | CBU_1183 | 27,449 | 27,399 | 7 | 121 | 0,9688 |
| Q83CE6 | CBU_1173 | 22,698 | 23,244 | 3 | 27 | 0,5159 |
| Q83CE9 | CBU_1169 | 26,618 | 27,421 | 9 | 125 | 0,0587 |
| Q83CF8 | CBU_1160 | 22,943 | 23,095 | 9 | 27 | 0,9549 |
| Q83CG4 | trpC | 21,450 | 22,106 | 7 | 19 | 0,2073 |
| Q83CG6 | CBU_1151 | 22,950 | 22,574 | 6 | 39 | 0,6737 |
| Q83CG9 | mfd | 22,397 | 23,831 | 26 | 78 | 0,0176 |
| Q83CH2 | yajC | 27,873 | 28,199 | 6 | 196 | 0,3843 |
| Q83CH3 | secD | 26,388 | 26,528 | 15 | 184 | 0,4727 |
| Q83CH4 | secF | 23,176 | 23,345 | 5 | 31 | 0,8399 |
| Q83CH7 | enhA.4 | 22,022 | 22,955 | 7 | 34 | 0,2212 |
| Q83CH8 | enhB.2 | 22,088 | 21,927 | 8 | 46 | 0,9707 |
| Q83CH9 | CBU_1136 | 23,955 | 24,984 | 30 | 146 | 0,2961 |
| Q83CI2 | suhB | 27,477 | 26,872 | 8 | 24 | 0,5333 |
| Q83CI4 | trmJ | 23,785 | 24,410 | 7 | 54 | 0,0593 |
| Q83CI6 | nifS | 21,900 | 21,411 | 7 | 24 | 0,3209 |
| Q83CJ1 | cbpM | 22,239 | 22,103 | 3 | 24 | 0,8744 |
| Q83CJ2 | cbpA | 24,533 | 24,992 | 12 | 99 | 0,1423 |
| Q83CJ3 | enhA.3 | 25,524 | 25,841 | 8 | 94 | 0,3017 |
| Q83CJ5 | CBU_1120 | 21,572 | 23,382 | 15 | 42 | 0,0161 |
| Q83CJ6 | CBU_1119 | 24,714 | 24,752 | 8 | 68 | 0,9234 |
| Q83CJ7 | etfB | 22,558 | 23,055 | 6 | 31 | 0,4716 |
| Q83CJ8 | etfA | 24,004 | 24,276 | 9 | 72 | 0,5076 |
| Q83CJ9 | ald | 24,589 | 25,051 | 15 | 86 | 0,0368 |
| Q83CK4 | CBU_1111 | 25,330 | 26,270 | 16 | 121 | 0,0203 |
| Q83CL1 | CBU_1103 | 23,168 | 21,875 | 9 | 31 | 0,0140 |
| Q83CL4 | CBU_1100 | 23,888 | 24,203 | 3 | 30 | 0,3812 |
| Q83CL5 | lepB-1 | 24,628 | 24,943 | 12 | 63 | 0,1970 |
| Q83CL6 | CBU_1098 | 23,224 | 22,695 | 14 | 62 | 0,3578 |
| Q83CL7 | aldC | 26,078 | 26,553 | 9 | 144 | 0,2279 |
| Q83CL8 | fumC | 26,312 | 26,814 | 18 | 169 | 0,1552 |
| Q83CL9 | CBU_1095 | 26,723 | 26,499 | 5 | 94 | 0,8402 |
| Q83CM0 | CBU_1094 | 26,582 | 27,254 | 20 | 246 | 0,0639 |
| Q83CM1 | CBU_1093 | 23,984 | 24,178 | 14 | 56 | 0,4224 |
| Q83CM3 | vacB | 25,556 | 25,830 | 27 | 173 | 0,0793 |
| Q83CM5 | nnr | 25,320 | 25,839 | 18 | 130 | 0,0080 |
| Q83CM7 | CBU_1085 | 24,052 | 25,312 | 13 | 79 | 0,0120 |
| Q83CM8 | CBU_1084 | 21,616 | 21,155 | 6 | 21 | 0,1257 |
| Q83CM9 | mutL | 22,968 | 22,808 | 11 | 34 | 0,8352 |
| Q83CN1 | CBU_1080 | 20,731 | 20,682 | 4 | 16 | 0,3361 |
| Q83CN4 | nagZ | 21,778 | 21,506 | 7 | 20 | 0,5660 |
| Q83CN7 | CBU_1073 | 22,366 | 22,532 | 5 | 28 | 0,7259 |
| Q83CN8 | tgt | 21,026 | 22,444 | 5 | 19 | 0,0086 |
| Q83CP4 | CBU_1065 | 22,684 | 23,232 | 5 | 36 | 0,3015 |
| Q83CP5 | CBU_1064 | 24,415 | 24,503 | 6 | 70 | 0,7345 |
| Q83CP8 | scpA | 22,396 | 22,921 | 10 | 42 | 0,4709 |
| Q83CP9 | scpB | 24,910 | 25,150 | 17 | 140 | 0,6583 |
| Q83CQ2 | mutS | 22,524 | 22,444 | 12 | 43 | 0,9785 |
| Q83CQ3 | CBU_1055 | 21,244 | 21,646 | 2 | 18 | 0,2959 |
| Q83CQ4 | recA | 26,495 | 26,663 | 15 | 175 | 0,8264 |
| Q83CQ6 | alaS | 26,913 | 27,615 | 38 | 402 | 0,1704 |
| Q83CQ7 | CBU_1051 | 24,046 | 24,720 | 10 | 75 | 0,0158 |
| Q83CQ8 | csrA2 | 23,717 | 23,902 | 4 | 21 | 0,6477 |
| Q83CR7 | cyoA | 24,920 | 25,083 | 10 | 65 | 0,6758 |
| Q83CR8 | cyoB | 22,765 | 22,956 | 6 | 34 | 0,6172 |
| Q83CS0 | CBU_1035 | 24,610 | 25,094 | 15 | 82 | 0,0309 |
| Q83CS6 | CBU_1027 | 25,850 | 25,928 | 15 | 148 | 0,8742 |
| Q83CT0 | CBU_1023 | 20,741 | 20,755 | 3 | 9 | 0,4184 |
| Q83CT2 | CBU_1021 | 23,978 | 24,094 | 5 | 56 | 0,8311 |
| Q83CT5 | CBU_1018 | 21,309 | 20,941 | 5 | 14 | 0,9423 |
| Q83CT6 | CBU_1017 | 21,192 | 20,761 | 7 | 26 | 0,8782 |
| Q83CU5 | bioB | 21,695 | 21,003 | 3 | 7 | 0,0761 |
| Q83CU7 | bioH | 25,232 | 24,084 | 2 | 24 | 0,0165 |
| Q83CV1 | lolC | 23,022 | 23,289 | 8 | 46 | 0,5208 |
| Q83CV2 | lolD | 23,428 | 24,267 | 8 | 58 | 0,0158 |
| Q83CV4 | purA | 25,110 | 25,195 | 13 | 108 | 0,6527 |
| Q83CV9 | def1 | 21,235 | 21,884 | 4 | 12 | 0,0588 |
| Q83CW4 | ung | 23,538 | 23,478 | 13 | 62 | 0,9549 |
| Q83CW5 | CBU_0987 | 22,996 | 22,878 | 7 | 26 | 0,9289 |
| Q83CW6 | rlmB | 24,152 | 24,635 | 9 | 72 | 0,0377 |
| Q83CW7 | CBU_0985 | 23,056 | 23,553 | 8 | 38 | 0,0374 |
| Q83CW9 | CBU_0982 | 23,405 | 23,505 | 8 | 41 | 0,5502 |
| Q83CX1 | CBU_0980 | 24,773 | 25,406 | 1 | 67 | 0,5154 |
| Q83CX2 | CBU_0979 | 26,219 | 27,214 | 6 | 136 | 0,1653 |
| Q83CX4 | CBU_0977 | 24,351 | 24,154 | 16 | 97 | 0,3027 |
| Q83CX5 | CBU_0976 | 23,796 | 23,524 | 8 | 70 | 0,6092 |
| Q83CX6 | CBU_0975 | 25,060 | 25,087 | 14 | 138 | 0,9470 |
| Q83CX7 | CBU_0974 | 24,479 | 24,587 | 11 | 82 | 0,6353 |
| Q83CX8 | CBU_0973 | 25,285 | 25,541 | 13 | 92 | 0,0952 |
| Q83CX9 | CBU_0972 | 23,334 | 23,382 | 6 | 34 | 0,8870 |
| Q83CY0 | pyrD | 22,804 | 23,392 | 7 | 41 | 0,3015 |
| Q83CY3 | CBU_0968 | 24,776 | 25,435 | 7 | 107 | 0,1021 |
| Q83CY5 | cydB | 21,070 | 21,064 | 3 | 9 | 0,0234 |
| Q83CY6 | cydA-2 | 25,169 | 25,345 | 12 | 100 | 0,6022 |
| Q83CY8 | bcp | 28,408 | 28,997 | 17 | 315 | 0,2106 |
| Q83CY9 | CBU_0962 | 26,491 | 27,173 | 16 | 154 | 0,0166 |
| Q83CZ5;Q83CR4 | gacA.3;gacA.4 | 23,546 | 23,164 | 7 | 37 | 0,1181 |
| Q83CZ8 | CBU_0952 | 26,886 | 26,921 | 15 | 153 | 0,9708 |
| Q83D01 | rhuM | 21,429 | 21,452 | 6 | 19 | 0,9685 |
| Q83D04 | CBU_0943 | 24,072 | 24,971 | 4 | 38 | 0,1865 |
| Q83D06 | CBU_0941 | 21,312 | 20,800 | 2 | 9 | 0,1011 |
| Q83D08 | CBU_0939 | 25,452 | 25,867 | 21 | 129 | 0,3580 |
| Q83D09 | CBU_0937 | 28,937 | 29,879 | 26 | 520 | 0,0165 |
| Q83D12 | CBU_0934 | 22,841 | 24,064 | 9 | 45 | 0,0345 |
| Q83D14 | glpK | 24,791 | 24,956 | 13 | 86 | 0,3132 |
| Q83D15 | glpD | 23,086 | 23,890 | 9 | 25 | 0,3353 |
| Q83D17 | CBU_0929 | 22,321 | 20,283 | 6 | 21 | 0,0975 |
| Q83D18 | pdxH | 25,560 | 25,558 | 9 | 103 | 0,9975 |
| Q83D21 | CBU_0925 | 22,595 | 23,541 | 7 | 41 | 0,1356 |
| Q83D22 | CBU_0924 | 22,899 | 23,394 | 6 | 27 | 0,2350 |
| Q83D25 | CBU_0921 | 23,081 | 23,184 | 3 | 25 | 0,9946 |
| Q83D28 | CBU_0916 | 23,049 | 23,672 | 8 | 41 | 0,0673 |
| Q83D29 | enhB.1 | 27,041 | 27,871 | 8 | 146 | 0,2166 |
| Q83D31 | CBU_0913 | 22,109 | 22,309 | 4 | 10 | 0,2162 |
| Q83D32 | prpD | 22,273 | 22,444 | 8 | 17 | 0,4905 |
| Q83D34 | CBU_0910 | 21,785 | 21,479 | 4 | 15 | 0,8691 |
| Q83D37 | yciL | 23,470 | 23,832 | 5 | 56 | 0,2611 |
| Q83D46 | CBU_0898 | 24,506 | 25,071 | 5 | 25 | 0,1313 |
| Q83D47 | purF | 25,433 | 26,180 | 13 | 125 | 0,0132 |
| Q83D49 | dedD | 25,015 | 25,666 | 8 | 56 | 0,0842 |
| Q83D50 | folC | 22,543 | 22,139 | 11 | 38 | 0,6525 |
| Q83D51 | accD | 24,293 | 24,547 | 10 | 69 | 0,2203 |
| Q83D52 | CBU_0891 | 25,443 | 25,634 | 6 | 89 | 0,6304 |
| Q83D53 | CBU_0890 | 21,896 | 21,849 | 3 | 6 | 0,9403 |
| Q83D54 | dsbA | 23,896 | 24,770 | 7 | 37 | 0,2801 |
| Q83D56 | coaBC | 21,049 | 21,032 | 3 | 9 | 0,2674 |
| Q83D58 | bipA | 22,434 | 22,310 | 8 | 34 | 0,9715 |
| Q83D66 | asd | 24,975 | 25,586 | 14 | 126 | 0,0208 |
| Q83D67 | aroC | 24,389 | 24,925 | 12 | 88 | 0,0706 |
| Q83D69 | udk | 22,108 | 22,265 | 3 | 20 | 0,6894 |
| Q83D71 | alr | 23,154 | 23,889 | 13 | 58 | 0,0836 |
| Q83D72 | dnaB | 25,168 | 25,814 | 18 | 125 | 0,0455 |
| Q83D73 | rplI | 26,575 | 26,460 | 10 | 115 | 0,8899 |
| Q83D75 | rpsR | 24,484 | 24,159 | 4 | 32 | 0,5440 |
| Q83D81 | CBU_0859 | 21,470 | 21,442 | 4 | 10 | 0,2584 |
| Q83D82 | nadE | 25,285 | 25,288 | 19 | 172 | 0,9973 |
| Q83D83 | lpxK | 21,131 | 21,327 | 7 | 17 | 0,5021 |
| Q83D84 | msbA | 23,951 | 24,544 | 14 | 80 | 0,0438 |
| Q83D87 | pnp | 26,298 | 26,305 | 34 | 280 | 0,9945 |
| Q83D88 | rpsO | 23,301 | 22,893 | 2 | 19 | 0,7624 |
| Q83D90 | galU | 23,960 | 24,081 | 6 | 65 | 0,4433 |
| Q83D91 | pgi | 21,274 | 21,322 | 5 | 22 | 0,8014 |
| Q83D92 | ugd | 23,172 | 22,354 | 8 | 75 | 0,4462 |
| Q83D93 | CBU_0845 | 24,267 | 24,243 | 17 | 87 | 0,9413 |
| Q83D94 | CBU_0844 | 22,278 | 22,350 | 8 | 43 | 0,9065 |
| Q83DA2 | CBU_0836 | 21,552 | 22,240 | 5 | 24 | 0,1614 |
| Q83DA3 | CBU_0835 | 21,083 | 21,138 | 2 | 7 | 0,3895 |
| Q83DA6 | CBU_0832 | 21,313 | 21,789 | 3 | 15 | 0,0329 |
| Q83DA7 | asnB-1 | 21,380 | 21,243 | 10 | 27 | 0,7511 |
| Q83DA8 | CBU_0830 | 22,377 | 22,860 | 13 | 50 | 0,6175 |
| Q83DA9 | CBU_0829 | 23,584 | 24,360 | 12 | 90 | 0,4165 |
| Q83DB0 | CBU_0828 | 24,001 | 22,897 | 11 | 27 | 0,0161 |
| Q83DB3 | CBU_0825 | 22,930 | 21,896 | 11 | 51 | 0,3677 |
| Q83DB4 | purB | 24,941 | 25,177 | 17 | 89 | 0,4554 |
| Q83DB5 | sfcA | 23,295 | 23,968 | 12 | 54 | 0,0204 |
| Q83DB6 | CBU_0822 | 21,362 | 21,367 | 5 | 13 | 0,9766 |
| Q83DB9 | CBU_0819 | 21,415 | 23,674 | 7 | 27 | 0,0173 |
| Q83DC0 | CBU_0818 | 22,261 | 22,849 | 6 | 37 | 0,1838 |
| Q83DC7 | prfC | 22,863 | 23,650 | 10 | 36 | 0,2287 |
| Q83DC8 | CBU_0810 | 21,211 | 21,477 | 4 | 15 | 0,6068 |
| Q83DD0 | valS | 25,697 | 25,502 | 27 | 120 | 0,5210 |
| Q83DD1 | CBU_0807 | 22,708 | 23,651 | 12 | 56 | 0,1248 |
| Q83DD4 | CBU_0804 | 23,951 | 24,772 | 16 | 96 | 0,0072 |
| Q83DD5 | CBU_0803 | 24,114 | 23,909 | 8 | 57 | 0,6536 |
| Q83DD6 | CBU_0802 | 25,924 | 25,979 | 9 | 54 | 0,9560 |
| Q83DE0 | CBU_0798 | 23,609 | 25,552 | 12 | 89 | 0,0164 |
| Q83DE2 | CBU_0796 | 23,853 | 24,138 | 3 | 34 | 0,2279 |
| Q83DE3 | folE | 25,231 | 25,729 | 13 | 112 | 0,0133 |
| Q83DE9 | CBU_0789 | 24,006 | 24,891 | 23 | 104 | 0,0351 |
| Q83DF0 | CBU_0788 | 21,273 | 21,057 | 5 | 6 | 0,4554 |
| Q83DF7 | gacA.2 | 25,610 | 25,469 | 9 | 74 | 0,5917 |
| Q83DG0 | CBU_0776 | 22,986 | 22,617 | 7 | 48 | 0,3571 |
| Q83DG4 | prpC | 23,737 | 23,193 | 9 | 37 | 0,2196 |
| Q83DG5 | prpB | 24,201 | 24,162 | 10 | 61 | 0,9320 |
| Q83DG6 | CBU_0770 | 22,979 | 23,224 | 9 | 42 | 0,4661 |
| Q83DG9 | CBU_0766 | 22,838 | 23,224 | 12 | 30 | 0,3137 |
| Q83DH1 | CBU_0762 | 23,634 | 24,396 | 6 | 53 | 0,0188 |
| Q83DH4 | bamD | 25,318 | 25,185 | 9 | 91 | 0,5202 |
| Q83DH6 | degP.2 | 26,382 | 27,401 | 15 | 147 | 0,0235 |
| Q83DH7 | CBU_0754 | 23,095 | 23,274 | 12 | 45 | 0,9415 |
| Q83DH8 | CBU_0753 | 21,542 | 23,324 | 10 | 38 | 0,0186 |
| Q83DH9 | CBU_0752 | 21,384 | 21,814 | 3 | 15 | 0,4191 |
| Q83DI0 | murA | 21,368 | 21,532 | 6 | 9 | 0,2215 |
| Q83DI1 | CBU_0750 | 24,204 | 24,540 | 9 | 59 | 0,0558 |
| Q83DI2 | CBU_0749 | 21,471 | 21,714 | 6 | 29 | 0,2621 |
| Q83DI3 | lptC | 21,451 | 20,764 | 4 | 8 | 0,3130 |
| Q83DI5 | CBU_0746 | 22,258 | 23,155 | 8 | 49 | 0,0211 |
| Q83DI6 | CBU_0745 | 23,390 | 25,402 | 4 | 30 | 0,0080 |
| Q83DI7 | ptsH | 23,027 | 23,577 | 2 | 7 | 0,5280 |
| Q83DJ0 | lon | 25,646 | 25,509 | 31 | 254 | 0,8294 |
| Q83DJ1 | clpX | 23,628 | 22,462 | 14 | 30 | 0,0558 |
| Q83DJ2 | clpP | 25,755 | 25,945 | 7 | 97 | 0,5264 |
| Q83DJ3 | tig | 26,356 | 26,091 | 20 | 173 | 0,3058 |
| Q83DJ4 | CBU_0736 | 22,550 | 22,882 | 8 | 33 | 0,3894 |
| Q83DK0 | CBU_0730 | 23,262 | 23,503 | 2 | 21 | 0,7681 |
| Q83DK1 | CBU_0729 | 22,351 | 23,612 | 8 | 41 | 0,1836 |
| Q83DK2 | CBU_0728 | 21,544 | 21,600 | 5 | 24 | 0,9667 |
| Q83DK3 | CBU_0727 | 21,940 | 21,643 | 3 | 16 | 0,8811 |
| Q83DK6 | CBU_0720 | 22,596 | 21,414 | 6 | 16 | 0,3349 |
| Q83DK7 | CBU_0719 | 21,503 | 22,005 | 2 | 15 | 0,4945 |
| Q83DK8 | CBU_0718 | 26,588 | 27,277 | 8 | 113 | 0,4955 |
| Q83DL0 | CBU_0714 | 21,261 | 21,639 | 2 | 18 | 0,2286 |
| Q83DL2 | gacA.1 | 21,621 | 21,610 | 5 | 16 | 0,7501 |
| Q83DM4 | CBU_0675 | 22,941 | 21,015 | 11 | 49 | 0,1352 |
| Q83DM5 | CBU_0674 | 24,870 | 23,376 | 14 | 97 | 0,1419 |
| Q83DN1 | dapD | 25,133 | 26,350 | 17 | 133 | 0,0199 |
| Q83DN2 | dapE | 25,026 | 26,364 | 14 | 128 | 0,0172 |
| Q83DN6 | CBU_0661 | 22,379 | 23,057 | 2 | 10 | 0,7510 |
| Q83DN8 | dnaZX | 23,270 | 23,766 | 19 | 81 | 0,6761 |
| Q83DN9 | CBU_0658 | 24,710 | 25,021 | 6 | 70 | 0,8487 |
| Q83DP0 | recR | 21,317 | 21,504 | 5 | 18 | 0,5892 |
| Q83DP8 | ribH | 26,263 | 26,996 | 6 | 90 | 0,0235 |
| Q83DP9 | ribA | 22,282 | 22,399 | 11 | 55 | 0,8586 |
| Q83DQ0 | ribE | 21,419 | 23,859 | 4 | 21 | 0,0125 |
| Q83DQ3 | ribD | 23,173 | 24,106 | 13 | 57 | 0,0169 |
| Q83DQ4 | CBU_0642 | 21,800 | 22,545 | 4 | 16 | 0,0677 |
| Q83DQ5 | CBU_0641 | 25,122 | 25,034 | 22 | 126 | 0,8565 |
| Q83DQ6 | CBU_0640 | 24,671 | 24,728 | 15 | 101 | 0,8060 |
| Q83DQ7 | CBU_0639 | 24,408 | 24,414 | 9 | 65 | 0,9804 |
| Q83DQ8 | CBU_0638 | 25,007 | 25,377 | 18 | 104 | 0,3011 |
| Q83DQ9 | CBU_0637 | 22,751 | 22,037 | 3 | 26 | 0,4667 |
| Q83DR2 | CBU_0634 | 21,516 | 21,197 | 11 | 34 | 0,7507 |
| Q83DR4 | CBU_0632 | 27,359 | 27,157 | 12 | 175 | 0,8704 |
| Q83DR5 | purL | 24,226 | 24,518 | 33 | 130 | 0,1618 |
| Q83DR6 | putA | 27,633 | 28,118 | 48 | 467 | 0,0363 |
| Q83DR7 | ppa | 27,255 | 27,146 | 11 | 158 | 0,6176 |
| Q83DS3 | tadA | 21,904 | 21,487 | 3 | 23 | 0,5676 |
| Q83DS4 | CBU_0621 | 24,969 | 24,406 | 12 | 104 | 0,0483 |
| Q83DS9 | CBU_0616 | 21,211 | 20,907 | 7 | 26 | 0,9781 |
| Q83DT0 | lpxD | 23,862 | 24,686 | 11 | 76 | 0,1906 |
| Q83DT1 | ompH | 29,586 | 29,798 | 15 | 299 | 0,6171 |
| Q83DT2 | yaeT | 27,323 | 27,227 | 34 | 361 | 0,8006 |
| Q83DT5 | mvaD | 21,103 | 21,913 | 14 | 30 | 0,1801 |
| Q83DU3 | cysQ-1 | 22,564 | 22,815 | 9 | 56 | 0,4178 |
| Q83DV1 | CBU_0591 | 22,301 | 22,559 | 8 | 20 | 0,8710 |
| Q83DV3 | CBU_0589 | 23,932 | 24,632 | 7 | 46 | 0,1412 |
| Q83DV4 | nadA | 23,210 | 23,329 | 9 | 22 | 0,6495 |
| Q83DV5 | glpE | 24,635 | 24,956 | 7 | 57 | 0,3130 |
| Q83DV6 | CBU_0586 | 24,358 | 24,761 | 25 | 133 | 0,0494 |
| Q83DV7 | CBU_0585 | 21,631 | 21,680 | 2 | 12 | 0,5888 |
| Q83DV9 | CBU_0583 | 25,051 | 24,920 | 4 | 60 | 0,8254 |
| Q83DW0 | bolA | 23,058 | 23,820 | 4 | 33 | 0,2069 |
| Q83DW2 | CBU_0580 | 21,265 | 21,921 | 6 | 17 | 0,7875 |
| Q83DW4 | CBU_0578 | 24,948 | 24,880 | 5 | 26 | 0,9561 |
| Q83DW6 | yfcX | 25,612 | 25,953 | 26 | 201 | 0,2965 |
| Q83DW8 | CBU_0574 | 24,524 | 25,330 | 11 | 76 | 0,0831 |
| Q83DW9 | CBU_0573 | 23,423 | 24,418 | 20 | 113 | 0,0604 |
| Q83DX0 | CBU_0572 | 25,927 | 27,197 | 20 | 276 | 0,0192 |
| Q83DX2 | CBU_0570 | 23,446 | 23,193 | 5 | 35 | 0,1976 |
| Q83DX3 | miaB | 22,397 | 22,654 | 9 | 38 | 0,4718 |
| Q83DX4 | CBU_0568 | 24,131 | 24,354 | 13 | 81 | 0,6053 |
| Q83DX7 | corC | 22,113 | 22,572 | 8 | 39 | 0,0324 |
| Q83DX9 | CBU_0562 | 21,335 | 21,771 | 10 | 41 | 0,6528 |
| Q83DY0 | CBU_0560 | 25,423 | 25,027 | 14 | 120 | 0,1419 |
| Q83DY1 | leuS | 25,664 | 25,929 | 21 | 151 | 0,0701 |
| Q83DY2 | lptE | 23,476 | 23,479 | 5 | 52 | 0,9912 |
| Q83DY3 | holA | 20,561 | 20,073 | 4 | 13 | 0,4836 |
| Q83DY4 | nadD | 21,008 | 21,460 | 4 | 13 | 0,9408 |
| Q83DY8 | pbpA | 23,342 | 24,445 | 10 | 67 | 0,0040 |
| Q83DZ0 | CBU_0548 | 23,815 | 24,050 | 16 | 78 | 0,6765 |
| Q83DZ2 | htpX | 24,682 | 25,377 | 5 | 49 | 0,1139 |
| Q83DZ3 | lemA | 27,065 | 28,214 | 15 | 258 | 0,0183 |
| Q83DZ6 | ligA | 26,180 | 26,872 | 24 | 111 | 0,0207 |
| Q83DZ7 | zipA | 22,901 | 23,494 | 10 | 46 | 0,4048 |
| Q83E02 | CBU_0535 | 26,521 | 27,182 | 18 | 180 | 0,1554 |
| Q83E05 | CBU_0532 | 23,048 | 24,142 | 2 | 35 | 0,3630 |
| Q83E06 | pyrF | 24,134 | 24,365 | 11 | 78 | 0,0953 |
| Q83E09 | rpsA | 27,701 | 27,702 | 30 | 371 | 0,9970 |
| Q83E10 | cmk | 22,723 | 23,567 | 10 | 49 | 0,0234 |
| Q83E11 | aroA | 23,534 | 23,910 | 9 | 52 | 0,0688 |
| Q83E12 | serC | 26,050 | 26,207 | 15 | 177 | 0,5108 |
| Q83E13 | gyrA | 25,583 | 26,078 | 31 | 221 | 0,1838 |
| Q83E15 | mtaD | 24,864 | 24,196 | 17 | 107 | 0,3683 |
| Q83E16 | leuA | 22,291 | 22,467 | 9 | 31 | 0,3586 |
| Q83E18 | uvrB | 23,721 | 24,523 | 17 | 85 | 0,0208 |
| Q83E19 | aspB | 26,409 | 26,307 | 18 | 170 | 0,8859 |
| Q83E24 | CBU_0510 | 28,336 | 28,633 | 11 | 303 | 0,6771 |
| Q83E25 | trpR | 21,665 | 22,029 | 2 | 12 | 0,2580 |
| Q83E28 | recJ | 23,538 | 22,132 | 13 | 61 | 0,1963 |
| Q83E31 | glnA | 27,783 | 27,812 | 16 | 266 | 0,9404 |
| Q83E32 | CBU_0502 | 24,493 | 25,302 | 9 | 55 | 0,0186 |
| Q83E35 | tmk | 23,450 | 23,837 | 11 | 62 | 0,2156 |
| Q83E37 | fabF | 25,708 | 25,844 | 12 | 127 | 0,6058 |
| Q83E38 | acpP | 27,872 | 27,614 | 4 | 94 | 0,6616 |
| Q83E39 | fabD | 23,911 | 23,756 | 7 | 61 | 0,8369 |
| Q83E40 | plsX | 25,608 | 25,400 | 20 | 169 | 0,4525 |
| Q83E41 | rpmF | 22,214 | 20,979 | 2 | 15 | 0,1258 |
| Q83E42 | CBU_0490 | 21,161 | 20,971 | 3 | 20 | 0,6549 |
| Q83E43 | CBU_0489 | 21,822 | 21,856 | 6 | 32 | 0,8961 |
| Q83E44 | CBU_0488 | 22,712 | 22,805 | 6 | 33 | 0,8396 |
| Q83E45 | rne | 26,862 | 26,718 | 26 | 267 | 0,7690 |
| Q83E49 | CBU_0482 | 24,329 | 24,362 | 9 | 37 | 0,9758 |
| Q83E50 | artP | 24,059 | 23,445 | 7 | 47 | 0,2082 |
| Q83E51 | argR | 22,218 | 22,903 | 5 | 17 | 0,5053 |
| Q83E52 | kdsB | 25,427 | 25,803 | 14 | 104 | 0,0207 |
| Q83E55 | CBU_0476 | 23,146 | 23,640 | 10 | 45 | 0,0896 |
| Q83E58 | CBU_0473 | 28,731 | 27,974 | 8 | 244 | 0,1760 |
| Q83E61 | CBU_0470 | 21,857 | 21,606 | 6 | 23 | 0,7267 |
| Q83E62 | CBU_0469 | 23,464 | 23,209 | 4 | 24 | 0,6165 |
| Q83E63 | xseB | 23,867 | 24,175 | 6 | 72 | 0,5948 |
| Q83E64 | bioC1 | 21,739 | 22,135 | 5 | 29 | 0,4065 |
| Q83E67 | lpdA | 26,777 | 27,472 | 28 | 298 | 0,1915 |
| Q83E68 | pdhC | 25,921 | 26,254 | 19 | 132 | 0,1426 |
| Q83E69 | pdhA | 27,459 | 27,625 | 45 | 441 | 0,3644 |
| Q83E75 | adk | 24,230 | 24,290 | 9 | 37 | 0,8082 |
| Q83E79 | ffh | 22,408 | 22,360 | 6 | 31 | 0,9811 |
| Q83E82 | CBU_0446 | 20,753 | 21,469 | 3 | 30 | 0,4178 |
| Q83E83 | rpsP | 24,698 | 24,270 | 7 | 65 | 0,4816 |
| Q83E85 | rplS | 25,549 | 25,534 | 7 | 82 | 0,9842 |
| Q83E94 | CBU_0433 | 22,390 | 22,814 | 2 | 24 | 0,2832 |
| Q83E96 | CBU_0431 | 23,401 | 24,135 | 7 | 52 | 0,0191 |
| Q83E97 | lysS | 25,555 | 25,378 | 20 | 157 | 0,4783 |
| Q83E98 | CBU_0429 | 21,108 | 22,665 | 4 | 14 | 0,0165 |
| Q83EA2 | panB | 21,576 | 20,421 | 4 | 17 | 0,0637 |
| Q83EA3 | panC | 22,641 | 22,650 | 9 | 43 | 0,8256 |
| Q83EA4 | panD | 20,870 | 21,221 | 5 | 14 | 0,5978 |
| Q83EA7 | CBU_0419 | 21,268 | 20,540 | 6 | 15 | 0,8913 |
| Q83EA8 | CBU_0418 | 22,811 | 23,004 | 7 | 26 | 0,8189 |
| Q83EC9 | ileS | 25,750 | 25,743 | 27 | 167 | 0,9789 |
| Q83ED0 | CBU_0395 | 26,695 | 26,812 | 12 | 150 | 0,7503 |
| Q83ED4 | ribF | 22,135 | 22,638 | 7 | 21 | 0,2597 |
| Q83ED5 | mviN | 20,776 | 20,838 | 2 | 9 | 0,9395 |
| Q83ED6 | rpsT | 23,032 | 20,553 | 1 | 18 | 0,0947 |
| Q83ED8 | obg | 22,966 | 23,258 | 12 | 50 | 0,2200 |
| Q83ED9 | rpmA | 25,049 | 24,176 | 3 | 46 | 0,1608 |
| Q83EE0 | rplU | 24,629 | 24,434 | 4 | 29 | 0,7346 |
| Q83EE1 | tag | 21,375 | 20,926 | 2 | 7 | 0,5569 |
| Q83EE2 | ispB | 21,667 | 21,684 | 5 | 18 | 0,6900 |
| Q83EE5 | ampD | 25,350 | 25,802 | 21 | 122 | 0,2166 |
| Q83EE6 | CBU_0378 | 21,814 | 21,553 | 4 | 16 | 0,2194 |
| Q83EF3 | CBU_0370 | 22,509 | 22,804 | 3 | 29 | 0,9559 |
| Q83EF5 | CBU_0368 | 25,479 | 25,341 | 14 | 119 | 0,6477 |
| Q83EF6 | phoB | 22,312 | 22,433 | 8 | 43 | 0,8153 |
| Q83EF7 | phoR | 23,096 | 23,246 | 8 | 38 | 0,4742 |
| Q83EG9 | CBU_0353 | 25,033 | 25,438 | 12 | 61 | 0,3009 |
| Q83EH1 | CBU_0351 | 23,096 | 23,759 | 8 | 46 | 0,4894 |
| Q83EH2 | gph | 21,184 | 22,245 | 5 | 28 | 0,0166 |
| Q83EH4 | CBU_0347 | 21,238 | 21,718 | 3 | 13 | 0,6890 |
| Q83EH5 | xylB | 21,084 | 21,848 | 8 | 21 | 0,0460 |
| Q83EH9 | pfkA | 22,144 | 23,169 | 4 | 11 | 0,1260 |
| Q83EI2 | pepN | 27,032 | 26,765 | 37 | 336 | 0,1410 |
| Q83EI3 | fis | 24,283 | 23,620 | 4 | 28 | 0,8283 |
| Q83EI4 | purH | 28,522 | 28,224 | 15 | 210 | 0,1754 |
| Q83EJ4 | purD | 27,608 | 28,627 | 21 | 252 | 0,1412 |
| Q83EJ8 | CBU_0322 | 21,108 | 21,064 | 7 | 13 | 0,0881 |
| Q83EJ9 | icmH | 20,865 | 21,733 | 3 | 9 | 0,0248 |
| Q83EK2 | dnaQ | 21,372 | 21,143 | 4 | 20 | 0,5272 |
| Q83EK3 | rnhA | 21,219 | 21,797 | 3 | 18 | 0,1868 |
| Q83EK5 | gloB | 21,312 | 21,794 | 5 | 27 | 0,1504 |
| Q83EK6 | CBU_0313 | 22,372 | 21,528 | 7 | 37 | 0,3693 |
| Q83EK7 | folD | 22,940 | 23,201 | 8 | 40 | 0,6768 |
| Q83EL0 | htpG | 27,154 | 27,674 | 38 | 406 | 0,5470 |
| Q83EL2 | CBU_0307 | 23,431 | 23,673 | 10 | 88 | 0,8814 |
| Q83EL5 | CBU_0304 | 25,839 | 25,953 | 3 | 77 | 0,9294 |
| Q83EL6 | rpoZ | 25,194 | 24,923 | 8 | 75 | 0,5199 |
| Q83EL7 | gmk | 23,560 | 24,075 | 8 | 16 | 0,5277 |
| Q83EL8 | yicC | 20,667 | 20,180 | 6 | 16 | 0,0206 |
| Q83EL9 | rph | 23,831 | 23,956 | 5 | 53 | 0,4716 |
| Q83EM0 | murI | 22,378 | 22,317 | 6 | 23 | 0,7355 |
| Q83EM1 | xth | 24,573 | 24,873 | 10 | 79 | 0,0206 |
| Q83EM3 | CBU_0294 | 22,836 | 22,894 | 9 | 61 | 0,9383 |
| Q83EM4 | rpmB | 22,813 | 22,094 | 4 | 25 | 0,5198 |
| Q83EM5 | rpmG | 23,312 | 23,280 | 3 | 29 | 0,9777 |
| Q83EM7 | coaD | 25,041 | 25,430 | 7 | 99 | 0,2348 |
| Q83EM9 | pcnB | 23,873 | 24,410 | 11 | 53 | 0,0145 |
| Q83EN0 | CBU_0285 | 20,798 | 20,516 | 3 | 18 | 0,5474 |
| Q83EN6 | CBU_0279 | 22,372 | 22,346 | 3 | 22 | 0,9398 |
| Q83EP0 | hemE | 22,027 | 21,927 | 6 | 41 | 0,4729 |
| Q83EP1 | uvrA | 26,017 | 27,603 | 34 | 195 | 0,0185 |
| Q83EP3 | CBU_0272 | 24,401 | 24,605 | 4 | 31 | 0,2585 |
| Q83EP4 | ssb | 27,231 | 27,522 | 10 | 139 | 0,6268 |
| Q83EP5 | CBU_0270 | 26,856 | 26,895 | 26 | 250 | 0,6948 |
| Q83EQ1 | rplQ | 23,932 | 23,991 | 5 | 29 | 0,9716 |
| Q83EQ2 | rpoA | 28,219 | 28,095 | 25 | 396 | 0,7060 |
| Q83EQ3 | rpsD | 26,247 | 26,133 | 16 | 109 | 0,8442 |
| Q83EQ4 | rpsK | 24,553 | 24,302 | 3 | 54 | 0,6724 |
| Q83EQ6 | secY | 23,053 | 22,765 | 4 | 19 | 0,5505 |
| Q83EQ7 | rplO | 23,367 | 22,336 | 5 | 30 | 0,3209 |
| Q83EQ9 | rpsE | 23,405 | 23,287 | 6 | 26 | 0,8874 |
| Q83ER0 | rplR | 24,092 | 24,363 | 5 | 39 | 0,8594 |
| Q83ER1 | rplF | 24,599 | 24,018 | 7 | 68 | 0,1474 |
| Q83ER2 | rpsH | 25,655 | 25,536 | 9 | 47 | 0,8701 |
| Q83ER3 | rpsN | 23,065 | 22,767 | 5 | 37 | 0,6588 |
| Q83ER4 | rplE | 25,684 | 25,399 | 12 | 92 | 0,4967 |
| Q83ER5 | rplX | 24,605 | 24,206 | 10 | 54 | 0,5829 |
| Q83ER6 | rplN | 24,784 | 24,609 | 7 | 67 | 0,7629 |
| Q83ER7 | rpsQ | 22,546 | 22,264 | 3 | 9 | 0,9217 |
| Q83ER8;REV__Q83ER8 | rpmC | 22,213 | 22,203 | 3 | 25 | 0,9194 |
| Q83ER9 | rplP | 24,187 | 24,238 | 6 | 43 | 0,9555 |
| Q83ES1 | rplB | 25,420 | 25,243 | 10 | 92 | 0,7629 |
| Q83ES2 | rplW | 23,742 | 23,297 | 1 | 24 | 0,5615 |
| Q83ES3 | rplD | 26,644 | 26,621 | 11 | 113 | 0,9780 |
| Q83ES4 | rplC | 25,657 | 25,464 | 12 | 131 | 0,7484 |
| Q83ES5 | rpsJ | 25,311 | 25,256 | 9 | 69 | 0,9440 |
| Q83ES6 | tufA | 30,068 | 30,026 | 34 | 699 | 0,9376 |
| Q83ES7 | fusA | 28,182 | 28,171 | 31 | 397 | 0,9677 |
| Q83ES8 | rpsG | 26,548 | 26,496 | 11 | 123 | 0,9427 |
| Q83ES9 | rpsL | 23,972 | 23,652 | 6 | 34 | 0,6094 |
| Q83ET0 | rpoC | 29,451 | 29,070 | 79 | 898 | 0,2015 |
| Q83ET2 | rplJ | 25,328 | 25,236 | 7 | 86 | 0,8892 |
| Q83ET3 | rplA | 26,510 | 26,375 | 11 | 144 | 0,8574 |
| Q83ET4 | rplK | 25,462 | 25,191 | 6 | 84 | 0,5950 |
| Q83ET5 | nusG | 26,045 | 26,041 | 11 | 127 | 0,9966 |
| Q83ET9 | CBU_0221 | 21,538 | 21,653 | 5 | 8 | 0,3874 |
| Q83EU4 | CBU_0215 | 26,638 | 26,922 | 15 | 209 | 0,5323 |
| Q83EV3 | gltX1 | 24,071 | 24,065 | 17 | 82 | 0,9800 |
| Q83EV7 | CBU_0201 | 23,140 | 23,680 | 6 | 35 | 0,5675 |
| Q83EV9 | coaA | 22,796 | 22,681 | 10 | 28 | 0,8647 |
| Q83EW0 | CBU_0198 | 22,399 | 22,937 | 8 | 25 | 0,0557 |
| Q83EW1 | CBU_0197 | 22,804 | 23,631 | 15 | 43 | 0,2828 |
| Q83EW4 | sda | 21,903 | 23,964 | 5 | 30 | 0,0067 |
| Q83EW5 | CBU_0193 | 20,963 | 20,415 | 3 | 6 | 0,3772 |
| Q83EX7 | tyrS | 25,850 | 26,154 | 17 | 174 | 0,2001 |
| Q83EX8 | CBU_0180 | 23,011 | 20,918 | 9 | 20 | 0,0343 |
| Q83EX9 | anmK | 22,144 | 22,653 | 7 | 43 | 0,1103 |
| Q83EY0 | CBU_0178 | 27,009 | 27,831 | 11 | 110 | 0,0477 |
| Q83EY1 | CBU_0177 | 24,926 | 25,687 | 9 | 39 | 0,0461 |
| Q83EY2 | degP.1 | 20,570 | 19,520 | 3 | 6 | 0,6167 |
| Q83F01 | coaE | 23,787 | 23,793 | 9 | 36 | 0,9869 |
| Q83F02 | queF | 22,946 | 22,867 | 7 | 34 | 0,9396 |
| Q83F03 | zapD | 23,446 | 24,287 | 11 | 63 | 0,0528 |
| Q83F06 | secA | 26,216 | 26,367 | 37 | 275 | 0,3398 |
| Q83F12 | ftsZ | 25,965 | 25,553 | 16 | 167 | 0,3666 |
| Q83F13 | ftsA | 25,103 | 25,074 | 18 | 154 | 0,9407 |
| Q83F14 | CBU_0139 | 21,087 | 21,688 | 2 | 27 | 0,0378 |
| Q83F17 | murC | 22,902 | 23,517 | 8 | 45 | 0,0496 |
| Q83F20 | murD | 24,145 | 24,379 | 16 | 102 | 0,4344 |
| Q83F27 | murF | 24,647 | 24,614 | 10 | 90 | 0,9214 |
| Q83F28 | murE | 23,225 | 23,753 | 11 | 48 | 0,0195 |
| Q83F33 | ftsI | 25,020 | 26,060 | 9 | 44 | 0,0479 |
| Q83F35 | rsmH | 22,181 | 22,981 | 7 | 22 | 0,0556 |
| Q83F36 | mraZ | 25,981 | 27,019 | 10 | 133 | 0,0478 |
| Q83F37 | CBU_0114 | 25,413 | 26,038 | 13 | 164 | 0,0864 |
| Q83F39 | tdh | 24,063 | 24,692 | 7 | 45 | 0,0320 |
| Q83F40 | kbl | 25,244 | 25,605 | 11 | 107 | 0,1916 |
| Q83F41 | CBU_0110 | 23,166 | 23,190 | 3 | 22 | 0,9234 |
| Q83F42 | CBU_0109 | 25,158 | 25,441 | 7 | 84 | 0,4887 |
| Q83F46 | CBU_0103 | 26,109 | 26,219 | 17 | 152 | 0,6413 |
| Q83F50 | sixA | 22,551 | 23,012 | 4 | 41 | 0,0529 |
| Q83F55 | clpB | 27,263 | 27,998 | 50 | 519 | 0,1043 |
| Q83F56 | queE | 21,592 | 22,501 | 4 | 24 | 0,3323 |
| Q83F57 | ybgF | 27,106 | 27,746 | 19 | 170 | 0,0501 |
| Q83F58 | CBU_0091 | 26,792 | 27,652 | 9 | 144 | 0,0156 |
| Q83F59 | tolB | 25,528 | 25,955 | 15 | 143 | 0,0502 |
| Q83F61 | CBU_0087 | 22,161 | 21,113 | 15 | 37 | 0,0695 |
| Q83F64 | CBU_0084 | 24,903 | 25,440 | 12 | 123 | 0,0363 |
| Q83F67 | proS | 25,384 | 25,667 | 27 | 158 | 0,2747 |
| Q83F72 | visC | 22,654 | 23,118 | 8 | 33 | 0,0341 |
| Q83F73 | ubiH | 22,467 | 23,124 | 12 | 46 | 0,2964 |
| Q83F75 | CBU_0073 | 26,816 | 26,948 | 25 | 205 | 0,6708 |
| Q83F81 | CBU_0067 | 22,012 | 20,551 | 2 | 7 | 0,1348 |
| Q83F82 | CBU_0066 | 20,399 | 20,419 | 4 | 22 | 0,5224 |
| Q83F83 | CBU_0065 | 25,267 | 25,568 | 8 | 64 | 0,1414 |
| Q83F84 | parE | 24,807 | 25,011 | 23 | 135 | 0,4776 |
| Q83F92 | CBU_0056 | 26,673 | 27,199 | 28 | 264 | 0,0553 |
| Q83F94 | ubiC | 21,945 | 22,940 | 8 | 31 | 0,0783 |
| Q83F97 | CBU_0051 | 22,512 | 22,255 | 7 | 32 | 0,9978 |
| Q83FA0 | CBU_0048 | 22,578 | 23,232 | 8 | 61 | 0,0156 |
| Q83FA1 | CBU_0045 | 27,152 | 27,591 | 14 | 158 | 0,1658 |
| Q83FA2 | CBU_0044 | 25,488 | 25,968 | 11 | 96 | 0,2497 |
| Q83FA3 | CBU_0043 | 22,502 | 22,927 | 5 | 33 | 0,2171 |
| Q83FA6 | prlC | 26,767 | 27,201 | 27 | 276 | 0,0205 |
| Q83FA7 | CBU_0035 | 23,771 | 24,062 | 9 | 60 | 0,6423 |
| Q83FA8 | acpP | 23,008 | 21,510 | 3 | 18 | 0,2544 |
| Q83FB1 | CBU_0031 | 23,931 | 23,037 | 12 | 53 | 0,2005 |
| Q83FB4 | rpiA | 21,136 | 21,300 | 5 | 23 | 0,8089 |
| Q83FB6 | csrA1 | 22,449 | 21,750 | 2 | 26 | 0,4677 |
| Q83FB7 | CBU_0023 | 21,294 | 21,075 | 3 | 12 | 0,2216 |
| Q83FB9 | CBU_0021 | 25,143 | 25,096 | 26 | 160 | 0,9553 |
| Q83FC0 | CBU_0020 | 23,684 | 23,807 | 8 | 35 | 0,8938 |
| Q83FC3 | deoC | 24,503 | 25,274 | 15 | 125 | 0,0187 |
| Q83FC4 | xapA | 24,082 | 24,426 | 13 | 66 | 0,3349 |
| Q83FD0 | aroE | 22,291 | 22,610 | 7 | 36 | 0,0714 |
| Q83FD5 | gyrB | 25,174 | 25,284 | 28 | 177 | 0,7363 |
| Q83FD6 | recF | 21,129 | 20,944 | 4 | 11 | 0,0159 |
| Q83FD7 | dnaN | 26,874 | 27,137 | 17 | 178 | 0,6502 |
| Q83FD8 | dnaA | 21,521 | 21,195 | 4 | 8 | 0,7477 |
| Q9KI19 | rpoS | 21,554 | 21,426 | 8 | 21 | 0,4729 |
| Q9KI21 | surE | 24,401 | 25,163 | 7 | 68 | 0,0140 |
| Q9X5U8 | rpsB | 26,201 | 26,015 | 14 | 139 | 0,6885 |
| Q9X5U9 | tsf | 25,759 | 25,874 | 16 | 163 | 0,9215 |
| Q9ZH99 | icd | 27,202 | 26,915 | 29 | 259 | 0,6493 |

# Table S3: LFQ analysis of all quantifiable proteins depicted as log2 average protein LFQ intensities (including values imputed by Perseus-type value imputation) for each analyses of cell-based cultures.
